# Hydrogen-bond activation enables aziridination of unactivated olefins with simple iminoiodinanes

**DOI:** 10.3762/bjoc.20.197

**Published:** 2024-09-11

**Authors:** Phong Thai, Lauv Patel, Diyasha Manna, David C Powers

**Affiliations:** 1 Department of Chemistry, Texas A&M University, College Station TX, 77843, USA

**Keywords:** aziridination, electrochemistry, H-bond activation, hypervalent iodine, nitrene transfer

## Abstract

Iminoiodinanes comprise a class of hypervalent iodine reagents that is often encountered in nitrogen-group transfer (NGT) catalysis. In general, transition metal catalysts are required to effect efficient NGT to unactivated olefins because iminoiodinanes are insufficiently electrophilic to engage in direct aziridination chemistry. Here, we demonstrate that 1,1,1,3,3,3-hexafluoroisopropanol (HFIP) activates *N*-arylsulfonamide-derived iminoiodinanes for the metal-free aziridination of unactivated olefins. ^1^H NMR and cyclic voltammetry (CV) studies indicate that hydrogen-bonding between HFIP and the iminoiodinane generates an oxidant capable of direct NGT to unactivated olefins. Stereochemical scrambling during aziridination of 1,2-disubstituted olefins is observed and interpreted as evidence that aziridination proceeds via a carbocation intermediate that subsequently cyclizes. These results demonstrate a simple method for activating iminoiodinane reagents, provide analysis of the extent of activation achieved by H-bonding, and indicate the potential for chemical non-innocence of fluorinated alcohol solvents in NGT catalysis.

## Introduction

Hypervalent iodine reagents find widespread application in selective oxidation chemistry due to the combination of synthetically tunable iodine-centered electrophilicity and the diversity of substrate functionalization mechanisms that can be accessed [1–2]. Large families of iodine(III)- and iodine(V)-based reagents have been developed – including iodobenzene diacetate (PhI(OAc)_2_, PIDA), Koser’s reagent (PhI(OH)OTs), Zhdankin’s reagent (C_6_H_4_(*o*-COO)IN_3_, ABX), and Dess–Martin periodinane (DMP) – and find application in an array of synthetically important transformations including olefin difunctionalization, carbonyl desaturation, alcohol oxidation, and C–H functionalization [3–4]. Iminoiodinanes (ArI=NR) are a subclass of hypervalent iodine reagents that function as nitrene equivalents in synthesis [5–6]. The direct reaction of iminoiodinanes with olefins, which could be envisioned to give rise to aziridines directly, is typically not observed and thus families of transition metal catalysts or photochemical procedures have been developed to enable this transformation [7–9].

**Scheme 1:**
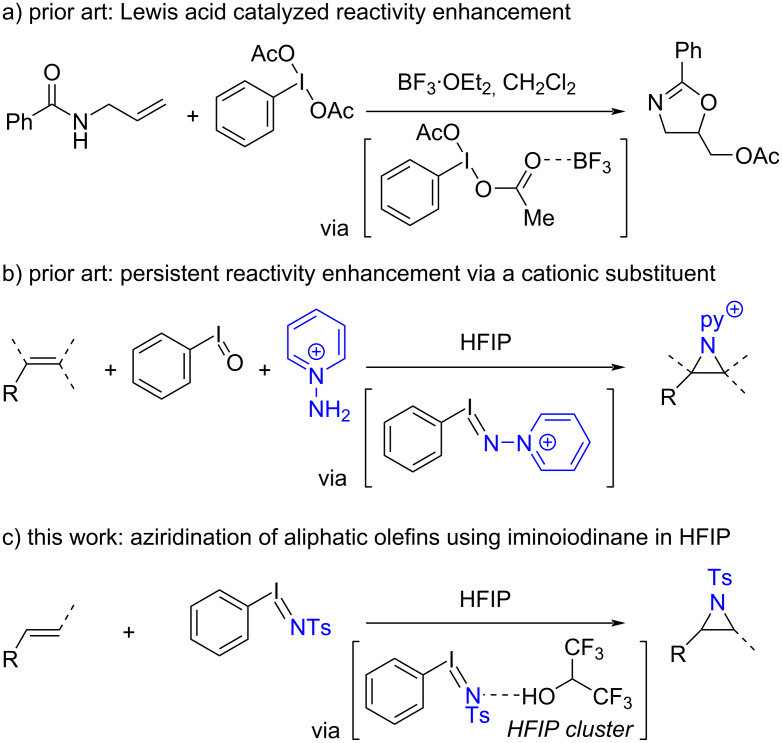
a) Lewis acid activation of hypervalent iodine reagents can enhance the reactivity of these reagents. b) Charge-tagged iminoiodinanes display enhanced reactivity in aziridination reactions with unactivated olefins (ref. [34]). c) Here, we demonstrate that H-bonding between fluorinated alcohol solvents and iminoiodinanes can enable direct metal-free aziridination of unactivated olefins with simple iminoiodinanes.

The reactivity of hypervalent iodine reagents can be enhanced via Lewis acid catalysis [10]. For example, PIDA becomes a stronger oxidant upon coordination of BF_3_·OEt_2_, enabling chemistry that was not available in the absence of Lewis acid activation (Scheme 1a) [11–12]. A variety of Lewis acid activators have been reported [13–22] in an array of group-transfer reactions, including trifluoromethylation, cyanation, and fluorination. Brønsted acid activation has also been described in some group-transfer schemes [23–25], and in particular, fluorinated alcohol solvents, such as 1,1,1,3,3,3-hexafluoroisopropanol (HFIP), have been reported to enhance hypervalent iodine reactivity by providing a H-bonding solvent cluster that enhances the electrophilicity of the iodine center [26–27]. Despite the prevalence of acid-activation in promoting carbon [28], oxygen [29–30], sulfur [31], chlorine [32], and fluorine [33] transfer reactions of hypervalent iodine compounds, these strategies have not been applied to activation of iminoiodinanes for nitrene transfer chemistry.

We recently developed a metal-free aziridination of unactivated olefins via the intermediacy of an *N*-pyridinium iminoiodinane (Scheme 1b) [34]. We rationalized the enhanced reactivity towards olefin aziridination as a result of charge-enhanced iodine-centered electrophilicity arising from the cationic *N*-pyridinium substituent. Based on those observations, we reasoned that similarly enhanced reactivity might be accessed by Lewis acid or H-bond activated iminoiodinanes. Here, we describe the HFIP-promoted aziridination of unactivated olefins with *N-*sulfonyl iminoiodinane reagents, which are among the most frequently encountered iminoiodinanes in NGT catalysis (Scheme 1c). This simple procedure afforded the formal transfer of various nitrogen groups, including those derived from complex amines, and is complementary to other metal-free aziridinations of unactivated olefins [35–39].

## Results and Discussion

Treatment of cyclohexene (**1a**) with a stoichiometric amount of simple iminoiodinane such as PhINTs (**2a**) in CH_2_Cl_2_ resulted in <10% conversion to the corresponding *N*-sulfonylaziridine **3a**, which is consistent with the previously reported need for transition metal catalysts to promote nitrene transfer catalysis (Table 1, entry 1) [40–41]. In contrast, combination of PhINTs (2.0 equiv) with **1a** in HFIP afforded **3a** in 67% NMR yield (Table 1, entry 2). Lowering the loading of iminoiodinane **2a** to 1.5 or 1.0 equivalents decreased the reaction yield to 28% and 22%, respectively (Table 1, entries 3 and 4). Increasing the reaction temperature negatively affected the efficiency of aziridination: Reactions performed at 30 or 50 °C afforded **3a** in 50% and 43% yield, respectively (Table 1, entries 5 and 6). Replacing HFIP with 2,2,2-trifluoroethanol (TFE), which is also a commonly encountered fluorinated alcohol solvent, resulted in a 16% yield of **3a** (Table 1, entry 7). Performing aziridination with 10 equivalents of HFIP in CH_2_Cl_2_ resulted in a 38% yield (Table 1, entry 8). The aziridine product **3a** was not observed when other Lewis or Brønsted acids, such as BF_3_·Et_2_O, TfOH, or Zn(OTf)_2_, were employed in CH_2_Cl_2_ (Table 1, entry 9). Attempts to generate **2a** in situ using 1 equivalent of TsNH_2_ (**4**) in combination with 1 or 2 equivalents of PhIO (**5**) resulted in aziridination yields of 19% and 27%, respectively (Table 1, entries 10 and 11). Finally, exclusion of ambient light had no impact of the aziridination of **1a** with PhINTs (Table 1, entry 12) [42–43].

**Table 1:** Optimization of HFIP-promoted aziridination of cyclohexene (**1a**). Conditions: 0.20 mmol **1a**, 0.40 mmol PhINTs **2a**, 1.0 mL HFIP, N_2_ atmosphere, 20 °C, 16 h. Yield was determined via ^1^H NMR using triethyl 1,3,5-benzenetricarboxylate as internal standard.

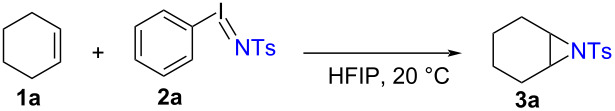		

Entry	Deviation from standard conditions	Yield (%)

**1**	CH_2_Cl_2_	<10%
**2**	none	67
**3**	1.5 equiv PhINTs	28
**4**	1.0 equiv PhINTs	22
**5**	30 °C	50
**6**	50 °C	43
**7**	TFE	16
**8**	10 equiv HFIP in CH_2_Cl_2_	38
**9**	5 equiv BF_3_·Et_2_O, TfOH, or Zn(OTf)_2_ in CH_2_Cl_2_,	0
**10**	1 equiv **4** and 1 equiv **5** instead of **2a**	19
**11**	1 equiv **4** and 2 equiv **5** instead of **2a**	27
**12**	no ambient light	64

**Scheme 2:**
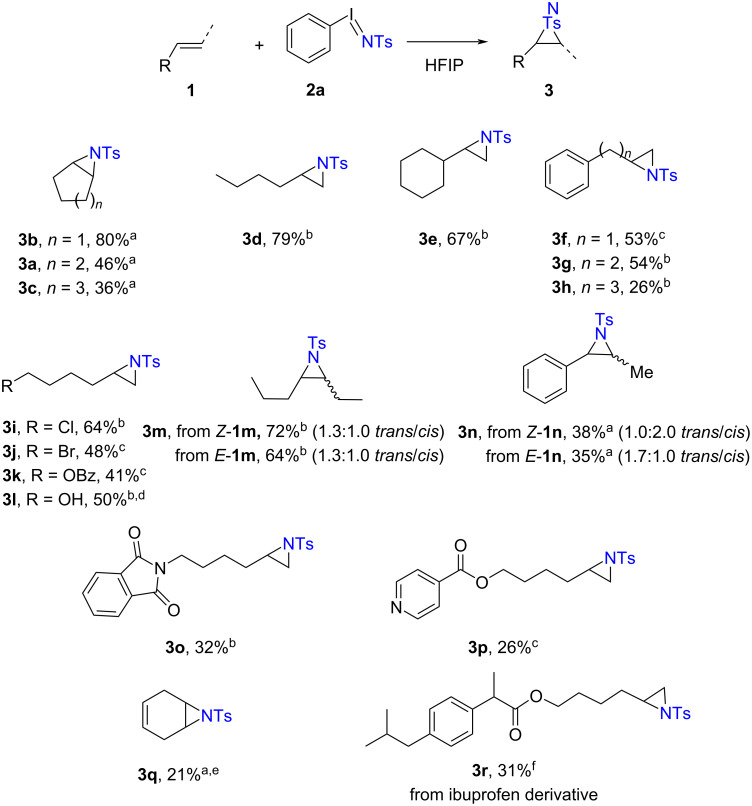
Scope and limitations of HFIP-promoted direct aziridination with iminoiodinane reagents. Conditions: 0.20 mmol **1**, 0.40 mmol **2a**, 1.0 mL HFIP, N_2_ atmosphere. a) 20 °C for 16 h, b) 50 °C for 16 h, c) 50 °C for 48 h, d) NMR yield, e) 1.2 equiv PhINTs was used, and f) 4.0 equiv of PhINTs at 20 °C for 48 h.

With metal-free aziridination conditions in hand, we explored the scope and limitations of the HFIP-promoted aziridination of unactivated olefins (Scheme 2). For cyclic substrates, aziridination of cyclopentene, cyclohexene, and cycloheptene afforded the corresponding aziridines in modest to high isolated yields: **3b** (80%), **3a** (46%), and **3c** (36%), respectively. Acyclic olefin 1-hexene underwent aziridination to **3d** in 79% yield (reaction performed at 50 °C); aziridination of vinylcyclohexane proceeded in 67% yield of **3e**. Allylbenzene engaged in aziridination to deliver **3f** in 53% yield, while homoallylbenzene and pent-4-en-1-ylbenzene underwent aziridination in yields of 54% (**3g**) and 26% (**3h**), respectively. The procedure was compatible with various commonly encountered functional groups, such as chloride (**3i**), bromide (**3j**), and benzoyl (**3k**). Noticeably, an unprotected alcohol is tolerated in our procedure, with product **3l** delivered at 50% NMR yield; **3l** is sensitive to column chromatography, and thus aziridine-opening to a cyclic ether was observed (31% isolated yield) during purification. Aziridination of *cis*- or *trans*-4-octene afforded aziridine **3m** as a 1.3:1.0 *trans*/*cis* mixture in 72% and 64% yield, respectively. While many styrene derivatives polymerize in HFIP [44], 1,2-disubstituted styrene derivatives were sufficiently stable to engage in the developed aziridination reaction, with *cis*- or *trans*-β-methylstyrene **1n** furnishing aziridine **3n** as diastereomeric mixtures with comparable yields of 38% (1.0:2.0 *trans*/*cis*, from *cis*-**1n**) and 35% (1.7:1.0 *trans*/*cis*, from *trans*-**1n**). Olefins containing *N*-heteroaromatics such as phthalimide and pyridine underwent aziridination to give **3o** (32% yield) and **3p** (26% yield). Similarly, 1,4-cyclohexadiene was compatible in this procedure, giving product **3q** in 21% yield (see Supporting Information File 1, Figure S1 for other challenging substrates). Finally, ibuprofen-derived olefin **1r** underwent aziridination to afford **3r** in 31% yield. Overall, these results highlight the efficacy of a simple activation protocol and the generality of H-bond activation of iminoiodinanes for direct aziridination, albeit with modest efficiency for some substrates.

**Scheme 3:**
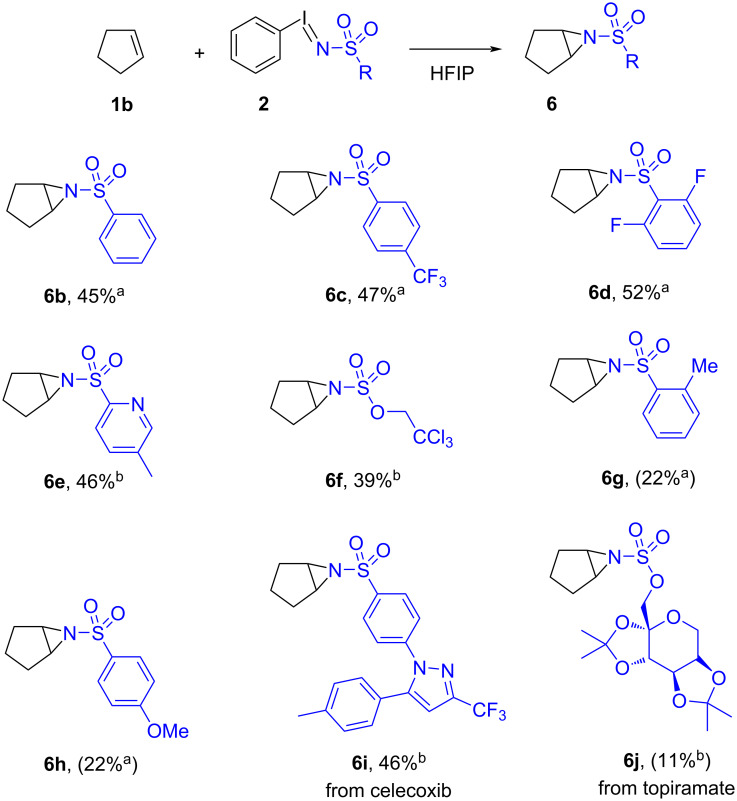
Scope of nitrogen group transfer in the aziridination of aliphatic olefins. Conditions using synthesized iminoiodinane: 0.20 mmol cyclopentene (**1b**), 0.40 mmol iminoiodinane **2**, 1.0 mL HFIP, N_2_ atmosphere. Conditions using in situ-generated iminoiodinane: 0.20 mmol cyclopentene (**1b**), 0.20 mmol sulfonamide, 0.40 mmol iodosylbenzene (PhIO), 1.0 mL HFIP, N_2_ atmosphere. a) 20 °C for 16 h, b) 40 °C for 16 h.

The impact of the iminoiodinane structure on the efficiency of HFIP-promoted direct aziridination was next investigated (Scheme 3). For this purpose, cyclopentene was selected as it underwent efficient aziridination with PhINTs. A family of iminoiodinanes **2** was synthesized from PIDA and the corresponding sulfonamide derivative. Reaction of phenylsulfonamide-derived iminoiodinane with cyclopentene afforded *N*-phenylsulfonylaziridine **6b** in 45% yield, while *N*-(*p*-trifluoromethylsulfonyl)aziridine **6c** was furnished in 47% yield. Similarly, 2,6-difluorosulfonyl-substituted iminoiodinane **2d** afforded aziridine **6d** in 52% yield. The aziridination procedure was tolerant of heterocyclic substituents on the iminoiodinane, *N*-(5-methylpyridin-2-ylsulfonyl)aziridine **6e** could be obtained in 46% yield. The *N*-Tces group (Tces = trichloroethylsulfamate) could also be transferred to afford **6f** in 39% yield. Finally, the iminoiodinane derived from celecoxib (**2i**) could be used to transfer this drug moiety to furnish aziridine **6i** in 46% yield. In general, the efficiency of aziridination correlates with the stability of the relevant iminoiodinane reagent, with higher yields attributed to more electron-rich sulfonamide substitution such as **2a**. Relatively electron-deficient iminoiodinanes are less efficient but are also more prone to decomposition (see Supporting Information File 1, Figure S2 for challenging iminoiodinanes). In situ preparation of the iminoiodinane intermediates is possible, and for those reagents that undergo facile decomposition, aziridination is more efficient using these conditions (yields for in situ-generated iminoiodinanes are in parentheses in Scheme 3, with *N*-*o*-methyl (**6g**) and *N*-*p*-methoxysulfonyl (**6h**) aziridines obtained each in 22% yield; the drug topiramate could also be transferred to furnish aziridine **6j** in 11% yield).

**Scheme 4:**
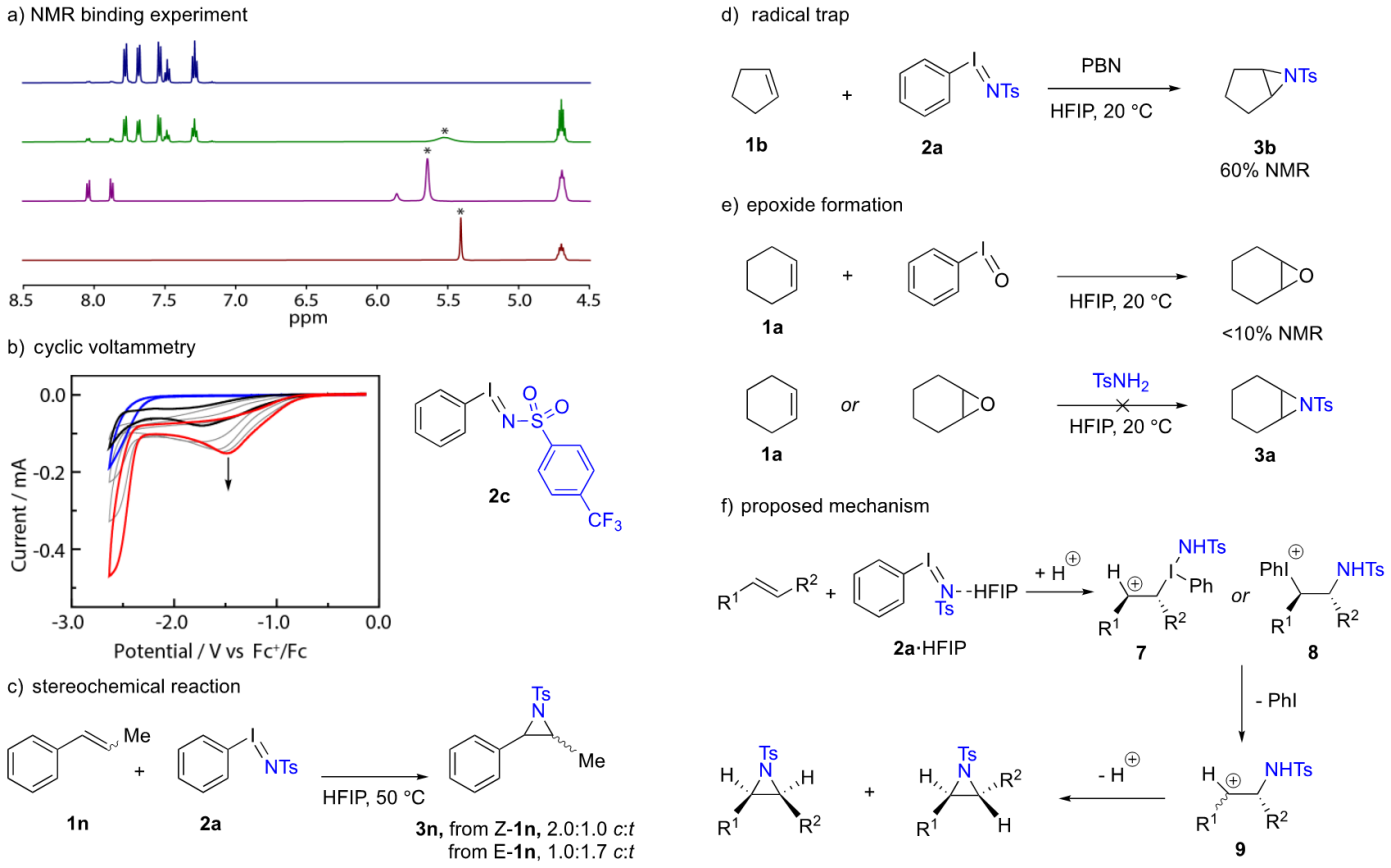
a) The broadening of the hydroxide proton (denoted by asterisk *) of HFIP in the presence of iminoiodinane **2c** suggesting hydrogen bonding observed in ^1^H NMR spectra (CD_3_CN) of: 8.0 mM **2c** with no HFIP (blue line), 8.0 mM **2c** with 32 mM HFIP (green line), 4.0 mM of 4-(trifluoromethyl)benzenesulfonamide with 32 mM HFIP (purple line), only 32 mM HFIP (red line). b) Cyclic voltammogram of iminoiodinane **2c** (8.0 mM) with varying amounts of HFIP in 5.0 mL solution of MeCN (0.10 M TBABF_4_) under N_2_ atmosphere: **2c** with no HFIP (black line); **2c** with 5, 10, 15 µL HFIP (grey line); **2c** with 25 µL HFIP (red line); only 25 µL (blue line). c) Diastereomeric mixtures of aziridines are obtained from aziridination reactions of *cis-* or *trans*-β-methylstyrene, suggesting aziridine formation likely to operate via a step-wise pathway. d) Aziridination is not impacted by the presence of potential radical traps. e) PhIO, potentially generated by PhINTs hydrolysis, can give rise to epoxidation products. Epoxides are not on-path to the observed aziridines. f) Proposed reaction mechanism.

We carried out a series of experiments to clarify the origin of the observed reactivity enhancement of *N*-arylsulfonyliminoiodinanes in the presence of HFIP (Scheme 4). First, ^1^H NMR was employed to examine the interaction between HFIP and iminoiodinane **2c** in CD_3_CN (compound **2c** was chosen over **2a** due to its increased solubility in nonprotic solvents). In a sample of **2c** with 4 equivalents of HFIP, a broad signal for O–H proton of HFIP was observed at 5.52 ppm with a FWHM = 56.6 Hz (Scheme 4a). This resonance was broader and more downfield than that of free HFIP in CD_3_CN (5.41 ppm with FWHM = 5.0 Hz), suggesting a hydrogen bonding interaction between HFIP and **2c**, and similar observations were also reported for the hydrogen bonding between HFIP and PIDA [30,33]. During this experiment, a small amount of hydrolysis product 4-(trifluoromethyl)benzenesulfonamide was also observed (1.2 mM, signals at 8.0, 7.9, and 5.86 ppm), but this compound did not greatly contribute to the broadening of O–H proton signal of HFIP as a separate 4.0 mM sample of the sulfonamide resulted in O–H proton signal of HFIP being at 5.64 ppm with FWHM = 11.3 Hz. Second, to evaluate the impact of HFIP on the redox chemistry of PhINTs, we collected cyclic voltammograms (CVs) of iminoiodinane **2c** in MeCN in the presence of varying HFIP increments (Scheme 4b). The CV of 25 µL HFIP in MeCN showed no electrochemical events between −2.0–0 V. The CV of **2c** in the absence of HFIP showed a reductive current (*i*_p_ = −0.80 mA) at peak potential (*E*_pr_) of –1.72 V vs Fc^+^/Fc. Upon addition of 5.0 µL of HFIP (1.2 equiv with respect to **2c**), the current increased to −1.22 mA, signaling the binding of HFIP to **2c** enhanced the electron transfer kinetics between the hypervalent iodine reagent and the electrode [45]. Further additions of HFIP further increased the current response and shifted the peak potential, with 10 µL and 15 µL of HFIP showing responses with *E*_pr_ at −1.55 V and −1.52 V, respectively. The titration showed a saturation point at 25 µL of HFIP (6.0 equiv with respect to **2c**), at which the CV of **2c** showed an *E*_pr_ = –1.47 V and –1.52 mA current. Overall, the addition of HFIP results in a 250 mV shift in the reduction of **2c**. The increased facility of reduction is consistent with H-bonding between HFIP and **2c**, which results in a more potent oxidant and gives rise to the observed HFIP-promoted olefin aziridination chemistry.

A number of observations are relevant to the mechanism by which unactivated olefin aziridination is accomplished by the HFIP-activated iminoiodinanes: First, the reaction of PhINTs with either *cis*- or *trans*-β-methylstyrene (**1n**) in HFIP afforded aziridine **3n** as a mixture of 2.0:1.0 *cis*/*trans* (from *cis*-**1n**) and 1.0:1.7 *cis*/*trans* (from *trans*-**1n**) (Scheme 4c). The formation of diastereomeric mixtures suggests that aziridination proceeds in a stepwise fashion. The dissimilarity of the diastereomeric ratios from *cis*- and *trans*- starting materials indicates that the potential intermediate is too short lived for complete ablation of the starting material stereochemistry. Second, the aziridination of cyclopentene by PhINTs in the presence of a radical trap *N*-*tert*-butyl-α-phenylnitrone (PBN) afforded the aziridine product **3b** in 60% NMR yield (Scheme 4d), suggesting a radical pathway was unlikely to be operative.

An ^1^H NMR experiment was carried out to probe the speciation of **2a** in HFIP, and we observed that **2a** underwent reversible ligand exchange with alcohol solvent to afford ArI(OR)_2_ and TsNH_2_ (Supporting Information File 1, Figure S3); similar solvolysis of PhIO in HFIP has been reported [10]. Reaction between cyclohexene and PhIO (2 equiv) in HFIP delivered <10% of cyclohexene oxide; meanwhile, both cyclohexene and cyclohexene oxide were shown to be unreactive towards sulfonamide (Scheme 4e), suggesting that epoxidation is not on path to the observed aziridines. For discussion of side-products and reaction mass balance, see Figure S4 (Supporting Information File 1). Based on these observations, we favor a mechanism in which H-bond activated iminoiodinane reacts directly with the olefin to generate a short-lived alkyl-bound iodinane **7** or iodonium species **8** (Scheme 4f). Ligand coupling from **7** or extrusion of iodobenzene from **8** would furnish a carbocation intermediate **9** which could undergo C–C bond rotation prior to ring closure to form the aziridine product. Such a process would account for the simultaneous stereochemical scrambling observed and the lack of radical trapping noted.

## Conclusion

In conclusion, we describe the activation of simple iminoiodinane reagents by fluorinated alcohols, such as HFIP. While most iminoiodinane reagents do not engage aliphatic olefins in the absence of transition metal catalysts, the addition of HFIP enables direct aziridination to be observed. The enhanced reactivity is rationalized as resulting from H-bonding between HFIP and the nitrogen center of the iminoiodinane reagents. ^1^H NMR data are consistent with such an association and electrochemical data collected in the presence of increasing HFIP concentrations are consistent with H-bonding affording an increasingly strong oxidant. These results demonstrate a simple method for activating iminoiodinane reagents and indicate the potential for chemical non-innocence of fluorinated alcohol solvents in NGT catalysis.

## Supporting Information

File 1 Experimental procedures and characterization data, original spectra of new compounds, and optimization details.

## Data Availability

All data that supports the findings of this study is available in the published article and/or the supporting information to this article.
