# Supplementary material for: Hydrogen-bond activation enables aziridination of unactivated olefins with simple iminoiodinanes
Source: Beilstein J Org Chem. 2024 Sep 11;20:2305–12. doi: 10.3762/bjoc.20.197 (PMC11406056; doi:10.3762/bjoc.20.197)
Supplement: File 1 — Experimental procedures and characterization data, original spectra of new compounds, and optimization details. [file Beilstein_J_Org_Chem-20-2305-s001.pdf]

BEILSTEIN JOURNAL OF ORGANIC CHEMISTRY

Supporting Information

for

Hydrogen-bond activation enables aziridination of unactivated olefins with simple iminoiodinanes

Phong Thai, Lauv Patel, Diyasha Manna and David C. Powers

Beilstein J. Org. Chem. **2024,** *20,* 2305–2312. doi:10.3762/bjoc.20.197

Experimental procedures and characterization data, original spectra of new compounds, and optimization details

License and Terms: This is a supporting information file under the terms of the Creative Commons Attribution License (https://creativecommons.org/licenses/by/4.0). Please note that the reuse, redistribution and reproduction in particular requires that the author(s) and source are credited and that individual graphics may be subject to special legal provisions.
The license is subject to the *Beilstein Journal of Organic Chemistry* terms and conditions: (https://www.beilstein-journals.org/bjoc/terms)

Supporting Information

Table of Contents

A. General considerations **S3**
A.1 Materials S3
A.2 Characterization details S3
A.3 Electrochemical experiment details. S4
B. Synthesis and characterization **S5**
B.1 Iminoiodinane reagents S5
B.1.1 Synthesis of arylsulfonamide precursors (**S1**) S5
B.1.2 Synthesis of iminoiodinane derivatives (**2**) S7
B.2 Aliphatic olefins S11
B.3 Aziridination of aliphatic olefins using PhINTs S13
B.4 Aziridination of cyclopentene using iminoiodinane derivatives S19
B.5 Aziridination of cyclopentene using in situ iminoiodinane derivatives S22
C. Reaction optimization studies **S24**
C.1 Optimization of aliphatic olefin aziridination S24
C.2 Challenging reaction partners S32
D. Evaluation of aziridination mechanism **S34**
E. NMR Spectra for new compounds **S40**
F. Supplementary references **S69**

A. General considerations

A.1 Materials All chemicals and solvents were obtained as ACS reagent grade and used as received. Potassium carbonate, vinylcyclohexane (**1e**), *trans*-4-octene ((*E*)-**1m**), and *cis*-4-octene ((*Z*)-**1m**) were purchased from BTC. 2,3,4,5,6-Pentafluorobenzenesulfonyl chloride was purchased from Matrix. Cyclohexene (**1a**), allylbenzene (**1f**), but-3-en-1-ylbenzene (**1g**), *trans*-β-methylstyrene ((*E*)-**1n**), 4-methylbenzenesulfonamide (**4**), isonicotinic acid, 1,3,5-trimethoxybenzene, triethyl benzene-1,3,5-tricarboxylate, boron trifluoride diethyl etherate (BF_3·OEt_2), methanol, ether, hexanes, ethyl acetate, and tetrahydrofuran were purchased from Sigma-Aldrich. Cyclopentene (**1b**), cycloheptene (**1c**), benzenesulfonyl chloride, and 4-nitrobenzenesulfonyl chloride were purchased from TCI. *N*-*tert*-Butyl-α-phenylnitrone and silver nitrate were purchased from Alfa Aesar. *N*,*N*-Dimethylformamide, concentrated hydrochloric acid, and aqueous ammonia (33%) were purchased from VWR. *cis*-β-Methylstyrene ((*Z*)-**1n**), 3,3,3-trichloroethyl sulfamate, 4-methoxybenzenesulfonamide, 5-methylpyridine-2-sulfonamide, and zinc trifluoromethanesulfonate ($Zn(OTf)_2$) were purchased from Ambeed. 1-Hexene (**1d**), 6-chloro-1-hexene (**1i**), 6-bromo-1-hexene (**1j**), hex-5-en-1-ol (**1l**), cyclohexa-1,4-diene (**1q**), phenyl-λ^3-iodanediyl diacetate, trifluoromethanesulfonamide, 2,6-difluorobenzenesulfonamide, trifluoromethanesulfonic acid (TfOH), 2-methylbenzenesulfonamide, celecoxib, benzoic acid, and ibuprofen were purchased from Oakwood. 1,1,1,3,3,3-Hexafluoro-2-propanol (HFIP) and 2,2,2-trifluoroethanol (TFE) were obtained from Oakwood, then refluxed for 12 h under N_2 over 4 Å molecular sieves, distilled, and stored in a Straus flask [1]. Dry dichloromethane and acetonitrile (purchased from Fisher scientific, HPLC grade) were obtained from a drying column and stored over activated 4 Å molecular sieves [2]. NMR solvents were purchased from Cambridge Isotope Laboratories and were used as received. All reactions were carried out under an ambient atmosphere unless otherwise noted. Iodosylbenzene (PhIO, **5**) [3], 4-methoxy-*N*-(phenyl-λ^3-iodaneylidene)benzenesulfonamide (**S2h**) [4], pent-4-en-1-ylbenzene (**1h**) [5], and 2-(hex-5-en-1-yl)isoindoline-1,3-dione (**1o**) [6] were synthesized according to literature methods.

A.2 Characterization details ^{1}H, ^{19}F, and ^{13}C NMR spectra were recorded on an Acsend™ 400 NMR (Bruker), an Avance III NMR (Bruker), or an Avance Neo 500 NMR (Bruker) and were referenced against residual proteo solvent signals: $CDCl_3$ (7.26 ppm, ^{1}H; 77.16 ppm, ^{13}C), $(CD_3)_2SO$ (2.50 ppm, ^{1}H; 39.52 ppm, ^{13}C), CD_3OD (3.31 ppm, ^{1}H, 49.00 ppm, ^{13}C), and CD_3CN (1.94 ppm, ^{1}H; 1.32 ppm, ^{13}C) [7]. ^{1}H NMR data are reported as follows: chemical shift (δ, ppm), (multiplicity: s (singlet), d (doublet), t (triplet), m (multiplet), br (broad), integration). ^{19}F NMR data are reported as follows: chemical shift (δ, ppm). ^{13}C NMR data are reported as follows: chemical shift (δ, ppm), d (doublet), t (triplet), q (quartet), m (multiplet). Mass spectrometry data were recorded on either Orbitrap Fusion™ Tribrid™ Mass Spectrometer or Q Exactive™ Focus Hybrid Quadrupole-Orbitrap™ Mass Spectrometer from ThermoFisher Scientific.

A.3 Electrochemical experiment details. Cyclic voltammetry (CV) experiments were carried out using CH Instruments Electrochemical Analyzer (Model CHI620A or CHI620E) in an undivided three-electrode cell under an N_2 atmosphere. A glassy carbon working electrode (circular surface, d = 3.0 mm), Pt counter electrode, and Ag reference electrode were used (obtained from CH Instruments). Reference electrode was prepared using 0.10 M solution of [TBA]PF_6 in MeCN with 1.0 mM $AgNO_3$, and IR compensation was not applied. The working electrode was polished using 0.5 µm alumina in a figure eight motion prior to each measurement, and the distance between the working and counter electrode was 11.0 mm. CV experiments are conducted at 20 °C, and scans start at 0.0 V vs Ag^+/Ag (–0.133 V vs Fc^+/Fc) and proceeds cathodically at a scan rate of 0.10 V/s.

B. Synthesis and characterization

B.1 Iminoiodinane reagents

B.1.1 Synthesis of arylsulfonamide precursors (S1)

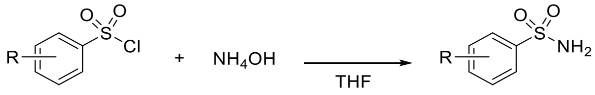

General Procedure Arylsulfonamide precursors (**S1**) were prepared according to the following modification of literature methods [8]. A 40 mL vial was charged with a suitable arylsulfonyl chloride (10.0 mmol, 1.00 equiv) and THF (5.0 mL). The vial was cooled to 0 °C using an ice bath and 33% aqueous ammonia (5.0 mL, 90 mmol, 9.0 equiv) was added. The solution was warmed to 20 °C and allowed to stir 16 h. The reaction mixture was then transferred to a separatory funnel and acidified with 4 M HCl until pH < 7. The aqueous solution was then extracted with EtOAc (3 × 30 mL). The combined organic fractions were washed with brine (30 mL), dried over Na_2SO_4, and concentrated under reduced pressure. The resulting solid was dried in vacuo and used without further purification.

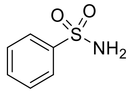

S1b

*Benzenesulfonamide (**S1b**)*. Prepared via general procedure from benzenesulfonyl chloride (1.76 g, 10.0 mmol) and obtained as a white solid (1.38 g, 88%). ^{1}H NMR (400 MHz, $CDCl_3$) δ 7.94 (d, *J* = 7.6 Hz, 2H), 7.60 (t, *J* = 7.2 Hz, 1H), 7.53 (t, *J* = 8.1 Hz, 2H), 4.73 (bs, 2H). The spectral data are well-matched to those reported in the literature [9].

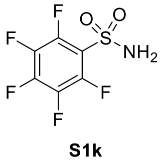

S1k

*2,3,4,5,6-Pentafluorobenzenesulfonamide (**S1k**)*. Prepared via general procedure from 2,3,4,5,6-pentafluorobenzenesulfonyl chloride (1.48 ml, 10.0 mmol) and obtained as an off white solid (1.38 g, 56%). ^{1}H NMR (400 MHz, $CDCl_3$) δ 5.42 (bs, 2H). ^{19}F NMR (377 MHz, $CDCl_3$) δ –137.1 (m), –145.9 (tt, *J* = 20.7, 6.4 Hz), –158.4 (m). The spectral data are well-matched to those reported in the literature [10].

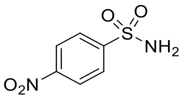

S1m

4-Nitrobenzenesulfonamide ***(S1m)***. Prepared via general procedure from 4-nitrobenzenesulfonyl chloride (2.22 g, 10.0 mmol) and obtained as a white solid (1.81 g, 90%). ^{1}H NMR (400 MHz, $(CD_3)_2SO$) δ 8.41 (d, *J* = 8.8 Hz, 2H), 8.06 (d, *J* = 8.9 Hz, 2), 7.72 (bs, 2H). The spectral data are well-matched to those reported in the literature [9].

B.1.2 Synthesis of iminoiodinane derivatives (2)

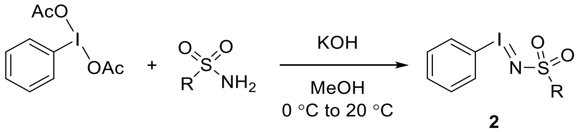

2

General Procedure Iminoiodinane derivatives (**2**) were prepared according to the modification of literature method [11]. A 40 mL vial was charged with an appropriate sulfonamide (2.50 mmol, 1.00 equiv), KOH (351 mg, 6.25 mmol, 2.50 equiv), and MeOH (10 mL). The vial was cooled to 0 °C, and phenyl-λ^3-iodanediyl diacetate (807 mg, 2.50 mmol, 1.00 equiv) was added in one portion. The reaction mixture was stirred at 0 °C for 30 min and then was allowed to warm to 23 °C at which temperature it was stirred for 3 h. The mixture was cooled to 0 °C, and ice-cold water (30 mL) was added. The reaction mixture was stirred at 0 °C until precipitation was observed. Solids were collected, washed with water (10 mL), ice-cold $CHCl_3$/Hex (10% v/v, 5 mL), and ether (10 mL), and dried *in vacuo.*

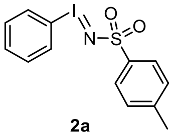

2a

*4-Methyl-N-(phenyl-λ^3-iodaneylidene)benzenesulfonamide (PhINTs, **2a**).* Prepared via the general procedure from *p*-toluenesulfonamide (2.57 g, 15.0 mmol), recrystallized from methanol, and obtained as an off white solid (4.03 g, 72%). ^{1}H NMR (400 MHz, $(CD_3)_2SO$) δ 7.69 (d, *J* = 8.4 Hz, 2H), 7.48–7.42 (m, 3H), 7.29 (t, *J* = 7.8 Hz, 2H), 7.06 (d, *J* = 7.8 Hz, 2H), 2.27 (s, 3H). These spectral data are well-matched to those reported in the literature [12].

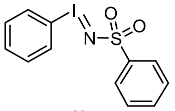

2b

*N-(Phenyl-λ^3-iodaneylidene)benzenesulfonamide (**2b**).* Prepared via the general procedure from benzenesulfonamide (**S1b**, 505 mg, 3.22 mmol) and obtained as a white solid (696 mg, 61%). ^{1}H NMR (400 MHz, $(CD_3)_2SO$) δ 7.72 (d, *J* = 7.6 Hz, 2H), 7.56 (d, *J* = 7.3 Hz, 2H), 7.45 (t, *J* = 7.4 Hz, 1H), 7.35 (t, *J* = 7.3 Hz, 1H), 7.31–7.27 (m, 4H). ^{13}C NMR (126 MHz, CD_3OD) δ 145.1, 133.3, 133.2, 132.2, 132.0, 130.0, 127.1, 122.6. HRMS-ESI$^+$ (m/z): $[M+H]^+$ calcd. for $C_{12}H_{10}INO_2S$, 359.9550; found, 359.9542.

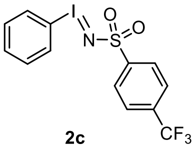

2c

N-(Phenyl-λ³-iodaneylidene)-4-(trifluoromethyl)benzenesulfonamide (***2c***). Prepared via general procedure from 4-(trifluoromethyl)benzenesulfonamide (563 mg, 2.50 mmol) and obtained as a white solid (725 mg, 68%). ^{1}H NMR (400 MHz, $(CD_3)_2SO$) δ 7.68 (t, *J* = 7.6 Hz, 4H), 7.56 (d, *J* = 8.1 Hz, 2H), 7.40 (t, *J* = 7.6 Hz, 1H), 7.24 (t, *J* = 7.9 Hz, 2H). ^{19}F NMR (377 MHz, $(CD_3)_2SO$) δ −61.5. ^{13}C NMR (126 MHz, CD_3OD) δ 138.6, 133.3, 132.2, 132.0, 131.4, 128.0, 127.1, 125.0 (q, *J* = 272.0 Hz), 122.6. HRMS-ESI^{+} (m/z): $[M+H]^{+}$ calcd. for $C_{13}H_9F_3INO_2S$, 427.9424; found, 427.9417.

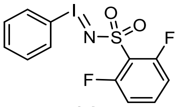

2d

2,6-Difluoro-N-(phenyl-λ³-iodaneylidene)benzenesulfonamide (***2d***). Prepared via general procedure from 2,6-difluorobenzenesulfonamide (483 mg, 2.50 mmol) and obtained as a white solid (710 mg, 72%). ^{1}H NMR (400 MHz, $(CD_3)_2SO$) δ 7.76 (d, *J* = 8.4 Hz, 2H), 7.43 (t, *J* = 7.3 Hz, 1H), 7.36–7.28 (m, 3H), 6.88 (t, *J* = 8.6 Hz, 2H). The spectral data are well-matched to those reported in the literature [11].

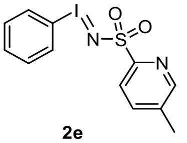

2e

5-Methyl-N-(phenyl-λ³-iodaneylidene)pyridine-2-sulfonamide (***2e***). Prepared via general procedure from 5-methylpyridine-2-sulfonamide (430 mg, 2.50 mmol) and obtained as a white solid (910 mg, 97%). ^{1}H NMR (400 MHz, $(CD_3)_2SO$) δ 8.35 (d, *J* = 2.0 Hz, 1H), 7.93 (d, *J* = 8.2 Hz, 2H), 7.71 (dd, *J* = 8.0, 2.1 Hz, 1H), 7.64 (d, *J* = 8.1 Hz, 1H), 7.55 (tt, *J* = 7.4, 2.1 Hz, 1H), 7.46 (tt, *J* = 7.4, 2.2 Hz, 2H), 2.32 (s, 3H). The spectral data are well-matched to those reported in the literature [13].

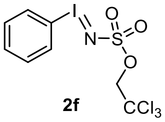

2f

2,2,2-Trichloroethyl (phenyl-λ³-iodaneylidene)sulfamate (PhINTces, ***2f****)*. Prepared via general procedure from 2,2,2-trichloroethyl sulfamate (360 mg, 1.58 mmol) and obtained as a white solid (552 mg, 82%). ^{1}H NMR (400 MHz, $(CD_3)_2SO$) δ 8.10 (d, *J* = 8.4 Hz, 2H), 7.62 (t, *J* = 7.6 Hz, 1H), 7.51 (t, *J* = 7.8 Hz, 2H), 4.22 (s, 2H). The spectral data are well-matched to those reported in the literature [12].

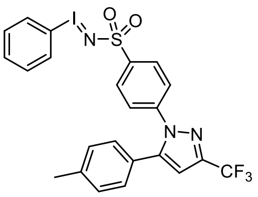

2i

*N-(Phenyl-λ³-iodaneylidene)-4-(5-(p-tolyl)-3-(trifluoromethyl)-1H-pyrazol-1-yl)benzenesulfonamide (**2i**)*. Prepared via general procedure from celecoxib (953 mg, 2.50 mmol) with an additional purification via trituration in diethyl ether (20 mL) and obtained as a white solid with 30% celecoxib impurity (909 mg, 49%). ^{1}H NMR (400 MHz, $CDCl_3$) δ 7.82 (d, *J* = 8.5 Hz, 2H), 7.76 (d, *J* = 7.9 Hz, 2H), 7.57 (t, *J* = 7.5 Hz, 1H), 7.39 (t, *J* = 7.9 Hz, 2H), 7.27–7.25 (m, 2H), 7.14 (d, *J* = 7.7 Hz, 2H), 7.07 (d, *J* = 7.9 Hz, 2H), 6.72 (s, 1H), 2.36 (s, 3H). ^{19}F NMR (377 MHz, $CDCl_3$) δ –60.82. ^{13}C NMR (126 MHz, $(CD_3)OD$) δ 147.1, 145.1, 144.8 (q, *J* = 38.2 Hz), 143.3, 141.0, 133.3, 132.2, 132.0, 130.6, 130.1, 128.3, 127.2, 127.0, 122. 7 (q, *J* = 268.0 Hz), 122.6, 106.9, 21.3. HRMS-ESI^{+} (m/z): $[M+H]^{+}$ calcd. for $C_{23}H_{17}F_3IN_3O_2S$, 584.0111; found, 584.0100.

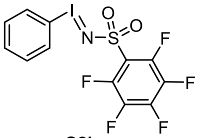

S2k

*2,3,4,5,6-Pentafluoro-N-(phenyl-λ³-iodaneylidene)benzenesulfonamide (**S2k**).* Prepared via general procedure from 2,3,4,5,6-pentafluorobenzenesulfonamide (**S1k**, 618 mg, 2.50 mmol) and obtained as a brown solid (619 mg, 55%). ^{1}H NMR (400 MHz, $(CD_3)_2SO$) δ 7.80 (d, *J* = 7.9 Hz, 2H), 7.50 (t, *J* = 7.3 Hz, 1H), 7.36 (t, *J* = 7.6 Hz, 2H). The spectral data are well-matched to those reported in the literature [4].

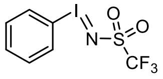

S2l

*1,1,1-Trifluoro-N-(phenyl-λ³-iodaneylidene)methanesulfonamide (PhINTf, **S2l**)*. Prepared via general procedure from trifluoromethanesulfonamide (373 mg, 2.50 mmol) and obtained as a white solid (273 mg, 31%). ^{1}H NMR (400 MHz, CD_3CN) δ 8.10 (d, *J* = 8.0 Hz, 2H), 7.70 (t, *J* = 7.5 Hz, 1H), 7.56 (t, *J* = 8.2 Hz, 2H). The spectral data are well-matched to those reported in the literature [14].

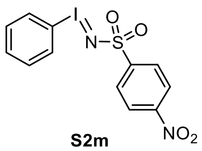

S2m

4-Nitro-N-(phenyl-λ³-iodaneylidene)benzenesulfonamide (PhINNs, ***S2m****)*. Prepared via general procedure from 4-nitrobenzenesulfonamide (**S1m**, 505 mg, 2.50 mmol) and obtained as a white solid (711 mg, 70%). ^{1}H NMR (400 MHz, $(CD_3)_2SO$) δ 8.03 (d, *J* = 8.7 Hz, 2), 7.72 (t, *J* = 8.9 Hz, 4H), 7.41 (t, *J* = 7.3 Hz, 1H), 7.25 (t, *J* = 7.8 Hz, 2H). The spectral data are well-matched to those reported in the literature [12].

B.2 Aliphatic olefins

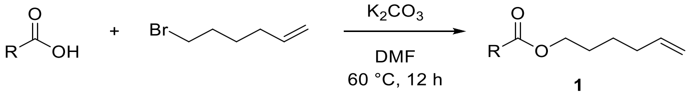

General Procedure A 20 mL scintillation vial was charged with the appropriate carboxylic acid (3.00 mmol, 1.00 equiv), K_2CO_3 (622 mg, 4.50 mmol, 1.50 equiv), and DMF (20.0 mL). To this vial, 6-bromohex-1-ene (440 µL mL, 3.30 mmol, 1.10 equiv) was added and the reaction mixture was stirred for 12 h at 60 °C. The solution was then cooled to 23 °C and water (15 mL) was added. The mixture transferred to a separatory funnel and extracted using EtOAc (3 × 10 mL). The combined organic fractions were washed with H_2O (4 × 10 mL), brine (30 mL), dried over Na_2SO_4, and concentrated in vacuo. The residue was purified by silica gel flash chromatography to give the corresponding olefin product (**1**).

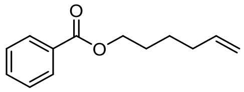

1k

*Hex-5-en-1-yl benzoate (**1k**).* Prepared via general procedure from benzoic acid (366 mg, 3.00 mmol), and obtained as a colorless oil (398 mg, 65%). ^{1}H NMR (400 MHz, $CDCl_3$) δ 8.05 (d, *J* = 8.3 Hz, 2H), 7.55 (t, *J* = 7.4 Hz, 1H), 7.44 (t, *J* = 7.7 Hz, 2H), 5.82 (ddt, *J* = 16.9, 10.1, 6.7 Hz, 1H), 5.09–4.92 (m, 2H), 4.33 (t, *J* = 6.6 Hz, 2H), 2.14 (q, *J* = 7.2 Hz, 2H), 1.82–1.75 (m, 2H), 1.59–1.53 (m, 2H). These spectral data are well-matched to those reported in the literature [15].

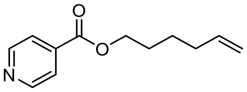

1p

*Hex-5-en-1-yl isonicotinate (**1p**).* Prepared via general procedure from isonicotinic acid (369 mg, 3.00 mmol) and obtained as a light brown oil (326 mg, 53%). ^{1}H NMR (400 MHz, $CDCl_3$) δ 8.77 (d, *J* = 5.9 Hz, 2H), 7.84 (d, *J* = 5.9 Hz, 2H), 5.80 (ddt, *J* = 16.9, 10.2, 6.7 Hz, 1H), 5.08–4.94 (m, 2H), 4.36 (t, *J* = 6.6 Hz, 2H), 2.13 (q, *J* = 7.1 Hz, 2H), 1.79 (dt, *J* = 15.1, 6.7 Hz, 2H), 1.54 (tt, *J* = 10.0, 6.4 Hz, 2H). These spectral data are well-matched to those reported in the literature [16].

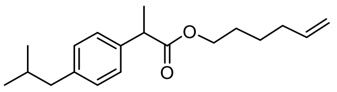

1r

*Hex-5-en-1-yl 2-(4-isobutylphenyl)propanoate (**1r**)*. Prepared via the general procedure from ibuprofen (618 mg, 3.00 mmol) and obtained as a yellow oil (253 mg, 44%). ^{1}H NMR (400 MHz, $CDCl_3$) δ 7.22 (d, *J* = 8.0 Hz, 2H), 7.11 (d, *J* = 8.0 Hz, 2H), 5.75 (ddt, *J* = 17.1, 10.3, 6.7 Hz, 1H), 5.09–4.92 (m, 2H), 4.12 (t, *J* = 6.7 Hz, 2H), 3.72–3.65 (m, 1H), 2.47 (d, *J* = 7.1 Hz, 2H), 2.07–1.99 (m, 2H), 1.87 (dd, *J* = 12.5, 5.6 Hz, 1H), 1.66–1.54 (m, 2H), 1.50 (d, J = 7.3 Hz, 3H), 1.36 (d, J = 9.7, 6.5 Hz, 2H), 0.90 (d, *J* = 6.7 Hz, 6H). These spectral data are well-matched to those reported in the literature [17].

B.3 Aziridination of aliphatic olefins using PhINTs

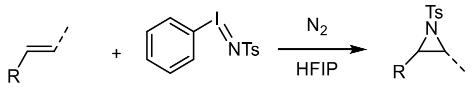

1 **2a** **3**

General Procedure A 20 mL scintillation vial was charged with the appropriate olefin (**1**, 0.200 mmol, 1.00 equiv) and dry HFIP (1.0 mL) under N_2 atmosphere. To this vial was added PhINTs (**2a**, 149 mg. 0.400 mmol, 2.00 equiv) as a single portion. The reaction was then stirred at 20 °C for 16 h (Procedure A), 50 °C for 16 h (Procedure B), or 50 °C for 48 h (Procedure C). The reaction was then concentrated in vacuo, and the residue was purified by silica gel flash chromatography using EtOAc:hexanes as eluent to afford the corresponding aziridine product (**3**).

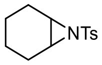

3a

*7-Tosyl-7-azabicyclo[4.1.0]heptane (**3a**)*. Prepared via Procedure A from cyclohexene (**1a**, 20.2 µL, 0.200 mmol) and obtained as a white solid (23.1 mg, 46%). ^{1}H NMR (400 MHz, $CDCl_3$) δ 7.81 (d, J = 8.3 Hz, 2H), 7.31 (d, J = 8.3 Hz, 2H), 3.02–2.96 (m, 2H), 2.43 (s, 3H), 1.84–1.73 (m, 4H), 1.46–1.35 (m, 2H), 1.28–1.17 (m, 2H). These spectral data are well-matched to those reported in the literature [18].

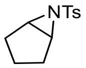

3b

*6-Tosyl-6-azabicyclo[3.1.0]hexane (**3b**)*. Prepared via Procedure A from cyclopentene (**1b**, 17.8 µL, 0.200 mmol) and obtained as a white solid (38.0 mg, 80%). ^{1}H NMR (400 MHz, $CDCl_3$) δ 7.82 (d, J = 8.3 Hz, 2H), 7.32 (d, J = 8.0 Hz, 2H), 3.33 (d, J = 1.6 Hz, 2H), 2.46–2.41 (s, 3H), 1.99–1.87 (m, 2H), 1.68–1.51 (m, 3H), 1.49–1.32 (m, 1H). These spectral data are well-matched to those reported in the literature [19].

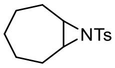

3c

*8-Tosyl-8-azabicyclo[5.1.0]octane (**3c**)*. Prepared via Procedure A from cycloheptene (**1c**, 23.3 µL, 0.200 mmol) and obtained as a white solid (19.1 mg, 36%). ^{1}H NMR (400 MHz, $CDCl_3$) δ 7.81 (d, J = 8.4 Hz, 2H), 7.32 (d, J = 8.3 Hz, 2H), 2.98–2.91 (m, 2H), 2.43 (s, 3H), 1.89–1.74 (m, 3H), 1.61–1.40 (m, 5H), 1.22–1.10 (m, 1H). These spectral data are well-matched to those reported in the literature [18].

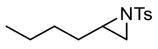

3d

*2-Butyl-1-tosylaziridine (**3d**)*. Prepared via Procedure B from 1-hexene (**1d**, 25.0 μL, 0.200 mmol) and obtained as a yellow oil (37.7 mg, 79%). ^{1}H NMR (400 MHz, $CDCl_3$) δ 7.82 (d, *J* = 8.3 Hz, 2H), 7.33 (d, *J* = 8.1 Hz, 2H), 2.71 (tt, *J* = 7.3, 4.8 Hz, 1H), 2.62 (d, *J* = 7.0 Hz, 1H), 2.44 (s, 3H), 2.05 (d, *J* = 4.5 Hz, 1H), 1.58–1.50 (m, 1H), 1.38–1.29 (m, 1H), 1.28–1.16 (m, 4H), 0.81 (t, *J* = 6.9 Hz, 3H). These spectral data are well-matched to those reported in the literature [20].

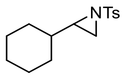

3e

*2-Cyclohexyl-1-tosylaziridine (**3e**)*. Prepared via Procedure B from vinylcyclohexane (**1e**, 27.3 μL, 0.200 mmol) and obtained as a yellow oil (37.3 mg, 67%). ^{1}H NMR (400 MHz, $CDCl_3$) δ 7.82 (d, *J* = 8.4 Hz, 2H), 7.33 (d, *J* = 8.1 Hz, 2H), 2.59 (d, *J* = 7.0 Hz, 1H), 2.53 (td, *J* = 7.1, 4.6 Hz, 1H), 2.44 (s, 3H), 2.09 (d, *J* = 4.6 Hz, 1H), 1.74–1.54 (m, 4H), 1.54–1.45 (m, 1H), 1.22–0.83 (m, 6H). These spectral data are well-matched to those reported in the literature [21].

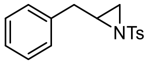

3f

*2-Benzyl-1-tosylaziridine (**3f**)*. Prepared via Procedure C from allylbenzene (**1f**, 26.4 μL, 0.200 mmol) and obtained as a white solid (30.6 mg, 53%). ^{1}H NMR (400 MHz, $CDCl_3$) δ 7.69 (d, *J* = 8.2 Hz, 2H), 7.21 (d, *J* = 8.2 Hz, 2H), 7.18–7.13 (m, 3H), 7.06–7.02 (m, 2H), 2.98–2.92 (m, 1H), 2.84–2.79 (dd, *J* = 14.5, 5.2 Hz, 1H), 2.71–2.65 (m, 2H), 2.42 (s, 3H), 2.16 (d, *J* =4.5 Hz ,1H). These spectral data are well-matched to those reported in the literature [20].

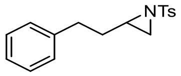

3g

*2-Phenethyl-1-tosylaziridine (**3g**)*. Prepared via Procedure B from but-3-en-1-ylbenzene (**1g**, 30.0 μL, 0.200 mmol) and obtained as a colorless oil (32.5 mg, 54%). ^{1}H NMR (400 MHz, $CDCl_3$) δ 7.83(d, *J* = 8.4 Hz, 2H), 7.34 (d, *J* = 8.0 Hz, 2H), 7.30–7.23 (m, 2H), 7.21–7.16 (m, 1H), 7.15–7.08 (m, 2H), 2.77 (tt, *J* = 7.6, 4.8 Hz, 1H), 2.66–2.53 (m, 3H), 2.45 (s, 3H), 2.05 (d, *J* = 4.6 Hz, 1H), 1.88 (dddd, J = 14.3, 9.3, 7.5, 5.0 Hz, 1H), 1.74–1.62 (m, 1H). These spectral data are well-matched to those reported in the literature [22].

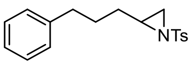

3h

*2-(3-Phenylpropyl)-1-tosylaziridine (**3h**)*. Prepared via Procedure B from pent-4-en-1-ylbenzene (**1h**, 29.2 mg, 0.200 mmol) and obtained as a colorless oil (16.4 mg, 26%). ^{1}H NMR (400 MHz, $CDCl_3$) δ 7.82 (d, *J* = 8.2 Hz, 2H), 7.31 (d, *J* = 8.0 Hz, 2H), 7.29–7.22 (m, 3H), 7.19–7.14 (m, 1H), 7.07 (d, *J* = 7.4 Hz, 2H), 2.74 (ddd, *J* = 9.0, 7.4, 4.5 Hz, 1H), 2.64 (d, *J* = 7.0 Hz, 1H), 2.74 (ddd, *J* = 9.0, 7.4, 4.5 Hz, 1H), 2.64 (d, *J* = 7.0 Hz, 1H), 2.55 (t, *J* = 7.5 Hz, 2H), 2.41 (s, 3H), 2.05 (d, *J* = 4.6 Hz, 1H), 1.67–1.51 (m, 3H), 1.39–1.29 (m, 1H). These spectral data are well-matched to those reported in the literature [23].

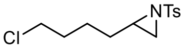

3i

*2-(4-Chlorobutyl)-1-tosylaziridine (**3i**)*. Prepared via Procedure B from 6-chlorohex-1-ene (**1i**, 26.2 μL, 0.200 mmol) and obtained as a colorless oil (36.8 mg, 64%). ^{1}H NMR (400 MHz, $CDCl_3$) δ 7.82 (d, *J* = 8.4 Hz, 2H), 7.34 (d, *J* = 8.0 Hz, 2H), 3.42 (td, *J* = 6.6, 1.0 Hz, 2H), 2.71 (tt, *J* = 7.0, 4.5 Hz, 1H), 2.64 (d, *J* = 7.0 Hz, 1H), 2.44 (s, 3H), 2.07 (d, *J* = 4.5 Hz, 1H), 1.75–1.57 (m, 3H), 1.44–123 (m, 3H). These spectral data are well-matched to those reported in the literature [22].

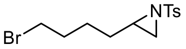

3j

*2-(4-Bromobutyl)-1-tosylaziridine (**3j**)*. Prepared via Procedure C from 6-bromohex-1-ene (**1j**, 26.7 μL, 0.200 mmol) and obtained as a colorless oil (31.5 mg, 48%). ^{1}H NMR (400 MHz, $CDCl_3$) δ 7.83 (d, *J* = 8.0 Hz, 2H), 7.35 (d, *J* = 8.2 Hz, 2H), 3.29 (td, *J* = 6.7, 1.5 Hz, 2H), 2.71 (tt, *J* = 7.2, 4.5 Hz, 1H), 2.64 (d, *J* = 7.0 Hz, 1H), 2.45 (s, 3H), 2.07 (d, *J* = 4.5 Hz, 1H), 1.84–1.73 (m, 2H), 1.68–1.57 (m, 1H), 1.43–1.29 (m, 3H). These spectral data are well-matched to those reported in the literature [24].

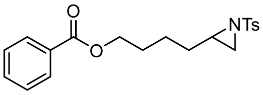

3k

*4-(1-Tosylaziridin-2-yl)butyl benzoate (**3k**)*. Prepared via Procedure C from hex-5-en-1-yl benzoate (**1k**, 40.9 mg, 0.200 mmol) and obtained as a yellow oil (30.7 mg, 41%). ^{1}H NMR (400 MHz, $CDCl_3$) δ 8.02 (d, *J* = 7.3 Hz, 2H), 7.82 (d, *J* = 8.0 Hz, 2H), 7.57 (t, *J* = 7.5 Hz, 1H), 7.45 (t, *J* = 7.6 Hz, 2H), 7.30 (d, *J* = 8.0 Hz, 2H), 4.21 (t, *J* = 6.5 Hz, 2H), 2.77–2.72 (m, 1H), 2.65 (d, *J* = 7.0 Hz, 1H), 2.40 (s, 3H), 2.08 (d, *J* = 4.5 Hz, 1H), 1.81–1.63 (m, 3H), 1.44–1.33 (m, 3H). These spectral data are well-matched to those reported in the literature [25].

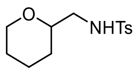

S3l

4-Methyl-N-((tetrahydro-2H-pyran-2-yl)methyl)benzenesulfonamide ***(S3l)***. Prepared via Procedure B from hex-5-en-1-ol (**1l**, 24.0 µL, 0.200 mmol) and obtained as a white solid (16.7 mg, 31%) (the uncyclized product 4-(1-tosylaziridin-2-yl)butan-1-ol (**3l**) was obtained in 50% NMR yield from the crude mixture). ^{1}H NMR (400 MHz, $CDCl_3$) δ 7.73 (d, *J* = 8.3 Hz, 2H), 7.30 (d, *J* = 7.9 Hz, 2H), 4.83 (dd, *J* = 8.5, 3.6 Hz, 1H), 3.93–3.86 (m, 1H), 3.38–3.28 (m, 2H), 3.07 (ddd, *J* = 12.0, 8.4, 3.2 Hz, 1H), 2.77 (ddd, *J* = 12.1, 8.3, 3.6 Hz, 1H), 2.42 (s, 3H), 1.84–1.76 (m, 1H),1.52–1.43 (m, 4H), 1.30–1.21 (m, 1H). These spectral data are well-matched to those reported in the literature [26].

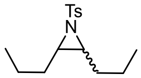

3m

2,3-Dipropyl-1-tosylaziridine ***(3m)***. Prepared via Procedure B from *cis*-oct-4-ene ((*Z*)-**1m**, 31.3 µL, 0.200 mmol), and obtained as a colorless oil (40.4 mg, 72%, 1.3 : 1.0 *trans*:*cis* mixture), or prepared via Procedure B from *trans*-oct-4-ene ((*E*)-**1m**, 31.3 µL, 0.200 mmol) and obtained as a colorless oil (36.0 mg, 64%, 1.3 : 1.0 *trans*:*cis* mixture). From (*Z*)-**1m**: ^{1}H NMR (400 MHz, $CDCl_3$) δ 7.86–7.78 (m, 3.56H, major + minor), 7.33–7.27 (m, 3.71H, major + minor), 2.78 (ddt, *J* = 8.8, 7.3, 3.6 Hz, 1.42H, minor), 2.68–2.59 (m, 2H, major), 2.45–2.37 (m, 5.23H, major + minor), 1.83–1.57 (m, 3.77H, major+ minor), 1.52–1.22 (m, 8.58H, major+ minor), 0.94–0.83 (m, 10H, major+ minor). From (*E*)-**1m**: ^{1}H NMR (400 MHz, $CDCl_3$) δ 7.86–7.78 (m, 3.73H, major + minor), 7.34–7.26 (m, 3.76H, major + minor), 2.78 (ddt, *J* = 8.8, 7.3, 3.6 Hz, 1.45H, minor), 2.67–2.60 (m, 2H, major), 2.46–2.39 (m, 5.44H, major + minor), 1.83–1.58 (m, 3.45H, major+ minor), 1.51–1.22 (m, 7,84H, major+ minor), 0.94–0.84 (m, 10.81H, major+ minor). These spectral data are well-matched to those reported in the literature [27].

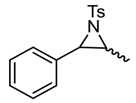

3n

2-Methyl-3-phenyl-1-tosylaziridine ***(3n)***. Prepared via Procedure A from *cis*-β-methylstyrene ((*Z*)-**1n**, 26.0 µL, 0.200 mmol), and obtained as a white solid (21.8 mg, 38%, 1.0 : 2.0 *trans*:*cis* mixture) or prepared via Procedure A from *trans*-β-methylstyrene ((*E*)-**1n**, 26.0 µL, 0.200 mmol) and obtained as a white solid (20.1 mg, 35%, 1.7 : 1.0 *trans*:*cis* mixture). From (*Z*)-**1n** ^{1}H NMR (400 MHz, $CDCl_3$) δ 7.90 (d, J = 8.4 Hz, 2H, major), 7.83 (d, J = 8.4 Hz, 1H, minor), 7.36–7.30 (m, 3.16H, major + minor), 7.29–7.23 (m, 6H, major + minor), 7.22–7.17 (m, 3.14H, major + minor), 7.16–7.11 (m, 2H, major + minor), 3.92 (d, *J* = 7.4 Hz, 1H, major), 3.79 (d, *J* = 4.4 Hz, 0.44H, minor), 3.19 (m, 1H, major), 2.91 (m, 0.45H, minor), 2.44 (s, 3H, major), 2.39 (s, 1.02H, minor), 1.85 (d, J = 6.0 Hz, 1.22H, minor), 1.04 (d, J = 5.8 Hz, 3H, major). From (*E*)-**1n** ^{1}H NMR (400 MHz, $CDCl_3$) δ 7.89 (d, J = 8.3 Hz, 1.12H, minor), 7.82 (d, J = 8.3 Hz, 2H, major), 7.36–7.30 (m, 2.24H, major + minor), 7.30–7.24 (m, 6H, major + minor), 7.21–7.19

(m, 1.38H, major + minor), 7.15–7.13 (m, 2H, major + minor), 3.93 (d, *J* = 7.3 Hz, 0.56H, minor), 3.79 (d, *J* = 4.3 Hz, 1H, major), 3.19 (dq, *J* = 7.1, 5.8 Hz, 0.51H, minor), 2.91 (qd, *J* = 6.0, 4.4 Hz, 1H, major), 2.44 (s, 1.92H, minor), 2.39 (s, 3H, major), 1.84 (d, J = 6.0 Hz, 3H, major), 1.02 (d, J = 5.8 Hz, 1.48H, minor). These spectral data are well-matched to those reported in the literature [28].

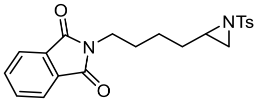

3o

*2-(4-(1-Tosylaziridin-2-yl)butyl)isoindoline-1,3-dione (**3o**)*. Prepared via Procedure B from 2-(hex-5-en-1-yl)isoindoline-1,3-dione (**1o**, 45.9 mg, 0.200 mmol) and obtained as a white solid (25.7 mg, 32%). ^{1}H NMR (400 MHz, CDCl$_3$) δ 7.90–7.77 (m, 4H), 7.77–7.65 (m, 2H), 7.34 (d, *J* = 8.0 Hz, 2H), 3.59 (t, *J* = 7.3 Hz, 2H), 2.71 (tt, *J* = 7.2, 4.8 Hz, 1H), 2.63 (d, *J* = 7.0 Hz, 1H), 2.44 (s, 3H), 2.07 (d, *J* = 4.5 Hz, 1H), 1.74–1.58 (m, 2H), 1.47–1.14 (m, 4H). These spectral data are well-matched to those reported in the literature [23].

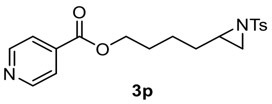

3p

*4-(1-Tosylaziridin-2-yl)butyl isonicotinate (**3p**)*. Prepared via Procedure C from hex-5-en-1-yl isonicotinate (**1p**, 41.1 mg, 0.200 mmol) and obtained as a brown oil (17.6 mg, 26%). ^{1}H NMR (400 MHz, CDCl$_3$) δ 8.78 (dd, *J* = 4.5, 1.5 Hz, 2H), 7.84–7.81 (m, 4H), 7.32 (d, *J* = 8.2Hz, 2H), 4.27 (t, *J* = 6.5 Hz, 2H), 2.80–2.76 (m, 1H), 2.63 (d, *J* = 7.2 Hz, 1H), 2.43 (s, 3H), 2.07 (d, *J* = 4.6 Hz, 1H), 1.79–1.67 (m, 3H) , 1.49–1.43 (m, 2H), 1.41–1.35 (m, 1H). ^{13}C NMR (126 MHz, CDCl$_3$) δ 165.3, 150.8, 144.7, 137.6, 135.3, 129.8, 128.2, 123.0, 65.5, 39.9, 34.1, 31.0, 28.1, 23.6, 21.8. HRMS-ESI$^+$ (m/z): [M+H]$^+$ calcd. for $C_{19}H_{23}N_2O_4S$, 375.1373; found, 375.1362.

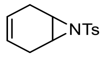

3q

*7-Tosyl-7-azabicyclo[4.1.0]hept-3-ene (**3q**)*. Prepared via Procedure A from cyclohexa-1,4-diene (**1q**, 18.9 µL, 0.200 mmol) using 1.2 equivalents of PhINTs (**2a**) and obtained as an off-white solid (10.5 mg, 21%). ^{1}H NMR (400 MHz, CDCl$_3$) 7.83 (d, J = 8.0Hz, 2H), 7.32 (d, *J* = 8.0 Hz, 2H), 7.26 (s, 1H), 5.44 (s, 2H), 3.11 (s, 2H), 2.44 (s, 3H), 2.37 (s, 4H). These spectral data are well-matched to those reported in the literature [29].

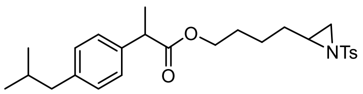

3r

*4-(1-Tosylaziridin-2-yl)butyl 2-(4-isobutylphenyl)propanoate (**3r**)*. Prepared via general procedure from hex-5-en-1-yl 2-(4-isobutylphenyl)propanoate (**1r**, 57.7 mg, 0.200 mmol) using 4.00 equiv of PhINTs (**2a**) at 20 °C for 48 h and obtained as a light yellow solid (28.1 mg, 31%). ^{1}H NMR (400 MHz, $CDCl_3$) δ 7.80 (d, *J* = 8.0 Hz, 2H), 7.30 (dd, *J* = 7.7, 5.3 Hz, 2H), 7.18 (d, *J* = 8.0 Hz, 2H), 7.08 (d, *J* = 8.0 Hz, 2H), 3.99–3.00 (m, 2H), 3.66 (qd, *J* = 7.2, 2.0 Hz, 1H), 2.69–2.64 (m, 1H), 2.60 (dd, *J* = 7.0, 2.8 Hz, 1H), 2.45–2.43 (m, 5H), 2.00 (t, *J* = 4.1 Hz, 1H), 1.89–1.79 (m, 1H), 1.55–1.47 (m, 6H), 1.32–1.18 (m, 3H), 0.89 (d, *J* = 6.8 Hz, 6H). ^{13}C NMR (126 MHz, $CDCl_3$) δ 174.8, 144.7, 140.7, 138.0, 135.4, 129.8, 129.4, 128.1, 127.3, 64.3, 45.3, 45.2, 40.1, 33.8, 33.8, 30.9, 30.3, 28.0, 23.3, 22.5, 21.7, 18.6. HRMS-ESI^{+} (m/z): $[M+H]^{+}$ calcd. for $C_{26}H_{36}NO_4S$, 458.2360; found, 458.2338.

B.4 Aziridination of cyclopentene using iminoiodinane derivatives

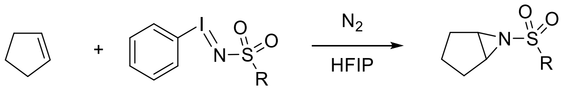

1b **2** **6**

General Procedure

A 20-mL scintillation vial was charged with cyclopentene (**1b**, 17.6 µL, 0.200 mmol, 1.00 equiv) and dry HFIP (1.0 mL) under N_2 atmosphere. To this vial was added the appropriate iminoiodinane (**2**, 0.400 mmol, 2.00 equiv) in a single portion. The reaction was then stirred for 16 h at 20 °C (Procedure D) or 40 °C (Procedure E). The mixture was then concentrated in vacuo, and the residue was purified by silica gel flash chromatography using EtOAc:Hexanes as eluent to afford the corresponding aziridine (**6**).

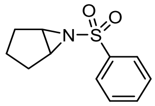

6b

6-(Phenylsulfonyl)-6-azabicyclo[3.1.0]hexane ***(6b)***. Prepared via Procedure D from *N*-(phenyl-λ^3-iodaneylidene)benzenesulfonamide (**2b**, 144 mg, 0.400 mmol) and obtained as a colorless oil (20.2 mg, 45%). ^{1}H NMR (400 MHz, $CDCl_3$) δ 7.93 (d, *J* = 7.3 Hz, 2H), 7.65–7.58 (m, 1H), 7.53 (dd, *J* = 8.5, 7.1 Hz, 2H), 3.36 (d, 2H), 1.99–1.89 (m, 2H), 1.68–1.53 (m, 3H), 1.46–1.33 (m, 1H). These spectral data are well-matched to those reported in the literature [30].

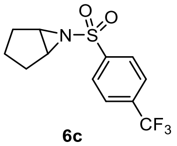

6c

6-((4-(Trifluoromethyl)phenyl)sulfonyl)-6-azabicyclo[3.1.0]hexane ***(6c)***. Prepared via Procedure D from *N*-(phenyl-λ^3-iodaneylidene)-4-(trifluoromethyl)benzenesulfonamide (**2c**, 171 mg, 0.400 mmol) and obtained as a colorless oil (27.1 mg, 47%). ^{1}H NMR (400 MHz, $CDCl_3$) δ 8.07 (d, *J* = 8.2 Hz, 2H), 7.80 (d, *J* = 8.2 Hz, 2H), 3.43 (s, 2H), 1.96 (dd, *J* = 13.9, 8.1 Hz, 2H), 1.70–1.59 (m, 3H), 1.43–1.34 (m, 1H). ^{19}F NMR (377 MHz, $CDCl_3$) δ –63.2. ^{13}C NMR (126 MHz, $CDCl_3$) δ 142.9, 135.0 (*J* = 33.1 Hz), 128.2, 126.3 (*J* = 3.5 Hz), 123.4 (*J* = 272.9 Hz), 47.6, 27.1, 19.5. HRMS-ESI$^+$ (m/z): $[M+H]^+$ calcd. for $C_{12}H_{13}F_3NO_2S$, 292.0614; found, 292.0606.

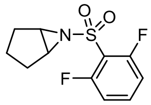

6d

*6-((2,6-Difluorophenyl)sulfonyl)-6-azabicyclo[3.1.0]hexane (**6d**).* Prepared via Procedure D from 2,6-difluoro-*N*-(phenyl-λ3-iodaneylidene)benzenesulfonamide (**2d**, 158 mg, 0.400 mmol) and obtained as a colorless oil (26.9 mg, 52%). ^{1}H NMR (400 MHz, $CDCl_3$) δ 7.54 (tt, *J* = 8.5, 5.9 Hz, 1H), 7.03 (t, *J* = 8.7 Hz, 2H), 3.59 (s, 2H), 2.04 (dd, *J* = 13.9, 7.9 Hz, 2H), 1.72–1.59 (m, 3H), 1.45–1.35 (m, 1H). ^{19}F NMR (377 MHz, $CDCl_3$) δ –105.5 (dd, *J* = 9.0, 6.2 Hz). ^{13}C NMR (126 MHz, $CDCl_3$) δ 159.9 (dd, *J* = 260.7, 3.8 Hz), 135.1 (t, *J* = 10.9 Hz), 117.6 (t, *J* = 15.8 Hz), 113.3 (dd, *J* = 23.4, 3.7 Hz), 48.1, 27.3, 19.4. HRMS-ESI+ (m/z): [M+H]+ calcd. for $C_{11}H_{12}F_2NO_2S$, 260.0551; found, 260.0544.

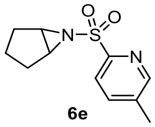

6e

*6-((5-Methylpyridin-2-yl)sulfonyl)-6-azabicyclo[3.1.0]hexane (**6e**).* Prepared via Procedure E from 5-methyl-*N*-(phenyl-λ3-iodaneylidene)pyridine-2-sulfonamide (**2e**, 150 mg, 0.400 mmol) and obtained as a white solid (21.9 mg, 46%). ^{1}H NMR (400 MHz, $CDCl_3$) δ 8.55 (s, 1H), 7.95 (d, *J* = 8.1 Hz, 1H), 7.70 (d, *J* = 8.2 Hz, 1H), 3.55 (s, 2H), 2.44 (s, 3H), 1.98 (dd, *J* = 13.8, 7.9 Hz, 2H), 1.69–1.65 (m, 2H), 1.60–1.54 (m, 1H), 1.44–1.33 (m, 1H). ^{13}C NMR (126 MHz, $CDCl_3$) δ 154.1, 150.8, 138.1, 137.9, 122.5, 47.4, 27.1, 19.5, 18.7. HRMS-ESI+ (m/z): [M+H]+ calcd. for $C_{11}H_{15}N_2O_2S$, 239.0849; found, 239.0844.

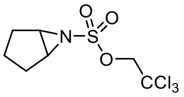

6f

*2,2,2-Trichloroethyl 6-azabicyclo[3.1.0]hexane-6-sulfonate (**6f**).* Prepared via Procedure E from 2,2,2-trichloroethyl (phenyl-λ3-iodaneylidene)sulfamate (**2f**, 172 mg, 0.400 mmol) and obtained as a white solid (23.0 mg, 39%). ^{1}H NMR (400 MHz, $CDCl_3$) δ 4.78 (s, 2H), 3.46 (s, 2H), 2.10 (dd, *J* = 13.3, 8.0 Hz, 2H), 1.78–1.60 (m, 3H), 1.50–1.35 (m, 1H). These spectral data are well-matched to those reported in the literature [31].

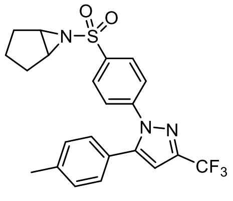

6i

*6-((4-(5-(p-Tolyl)-3-(trifluoromethyl)-1H-pyrazol-1-yl)phenyl)sulfonyl)-6-azabicyclo[3.1.0]hexane (**6i**).* Prepared via Procedure E from *N*-(phenyl-*λ*3-iodaneylidene)-

4-(5-(*p*-Tolyl)-3-(trifluoromethyl)-1*H*-pyrazol-1-yl)benzenesulfonamide (**2i**, 70 mol% pure as a mixture with celecoxib, 299 mg, 0.400 mmol) and obtained as a white solid (41.1 mg, 46%). ^{1}H NMR (400 MHz, $CDCl_3$) δ 7.92 (d, *J* = 8.7 Hz, 2H), 7.49 (d, *J* = 8.7 Hz, 2H), 7.18 (d, *J* = 8.0 Hz, 2H), 7.11 (d, *J* = 8.1 Hz, 2H), 6.75 (s, 1H), 3.37 (s, 2H), 2.38 (s, 3H), 1.94 (dd, *J* = 13.7, 8.0 Hz, 2H), 1.68–1.57 (m, 3H), 1.42–1.32 (m, 1H). ^{19}F NMR (377 MHz, $CDCl_3$) δ –62.5. ^{13}C NMR (126 MHz, $CDCl_3$) δ 145.5, 144.4 (q, *J* = 38.7 Hz), 143.1, 140.0, 138.8, 129.9, 128.9, 128.8, 125.8, 125.6, 121.2 (q, *J* = 269.3 Hz), 106.5, 47.4, 27.1, 21.5, 19.6. HRMS-ESI^{+} (m/z): $[M+H]^{+}$ calcd. for $C_{22}H_{21}F_3N_3O_2S$, 448.1301; found, 448.1290.

B.5 Aziridination of Cyclopentene using *in situ* Iminoiodinane derivatives

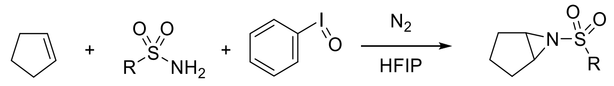

1b 6

General Procedure

A 20-mL scintillation vial was charged with cyclopentene (**1b**, 17.6 µL, 0.200 mmol, 1.00 equiv), the appropriate sulfonamide (0.200 mmol, 1.00 equiv), and dry HFIP (1.0 mL) under N_2 atmosphere. To this vial was added iodosylbenzene (PhIO, 88.0 mg, 0.400 mmol, 2.00 equiv) in a single portion. The reaction was then stirred for 16 h at 20 °C (Procedure F) or 40 °C (Procedure G). The mixture was then concentrated in vacuo, and the residue was purified by silica gel flash chromatography using EtOAc:hexanes as eluent to afford the corresponding aziridine (**6**).

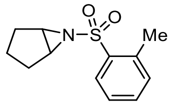

6g

6-(o-Tolylsulfonyl)-6-azabicyclo[3.1.0]hexane (***6g***). Prepared via Procedure F from 2-methylbenzenesulfonamide (34.2 mg, 0.200 mmol) and obtained as a white solid (10.4 mg, 22%). ^{1}H NMR (400 MHz, $CDCl_3$) 7.93 (dd, *J* = 7.9, 1.5 Hz, 1H), 7.48 (td, *J* = 7.6, 1.5 Hz, 1H), 7.37–7.28 (m, 2H), 3.39 (s, 2H), 2.76 (s, 3H), 1.98–1.93 (m, 2H), 1.66–1.58 (m, 3H), 1.42–1.36 (m, 1H). ^{13}C NMR (126 MHz, $CDCl_3$) δ 138.8, 137.8, 133.2, 132.6, 128.9, 126.0, 46.9, 27.2, 20.7, 19.7. HRMS-ESI$^+$ (m/z): [M+H]$^+$ calcd. for $C_{12}H_{15}NO_2S$, 238.0896; found, 238.0893.

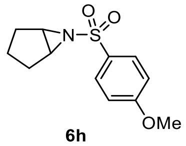

6h

6-((4-Methoxyphenyl)sulfonyl)-6-azabicyclo[3.1.0]hexane (***6h***). Prepared via Procedure F from 4-methoxybenzenesulfonamide (37.4 mg, 0.200 mmol) and obtained as a white solid (11.2 mg, 22%). ^{1}H NMR (400 MHz, $CDCl_3$) 7.86 (d, *J* = 9.0 Hz, 1H), 6.99 (d, *J* = 9.0 Hz, 1H), 3.88 (s, 3H), 3.31 (s, 2H), 1.97–1.90 (m, 2H), 1.65–1.57 (m, 3H), 1.44–1.37 (m, 1H). ^{13}C NMR (126 MHz, $CDCl_3$) δ 163.5, 130.9, 129.9, 114.3, 55.8, 46.8, 27.1, 19.7. HRMS-ESI$^+$ (m/z): [M+H]$^+$ calcd. for $C_{12}H_{15}NO_3S$, 253.0845; found, 254.0842.

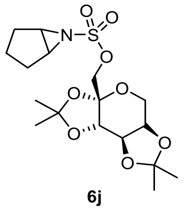

6j

((3aS,5aS,8aR,8bS)-2,2,7,7-Tetramethyltetrahydro-3aH-bis([1,3]dioxolo)[4,5-b:4',5'-d]pyran-3a-yl)methyl 6-azabicyclo[3.1.0]hexane-6-sulfonate ***(6j)***. Prepared via Procedure G from topiramate (67.8 mg, 0.200 mmol) and obtained as a white solid (8.6 mg, 11%). ^{1}H NMR (400 MHz, $CDCl_3$) 4.62 (dt, *J* = 8.0, 2.4 Hz, 1H), 4.41–4.36 (m, 2H), 4.32 (dd, *J* = 10.3, 2.2 Hz, 1H), 4.24 (dd, *J* = 8.2, 1.7 Hz, 1H), 3.92 (dt, *J* = 13.0, 2.1 Hz, 1H), 3.78 (dd, *J* = 13.0, 2.3 Hz, 1H), 3.35 (s, 2H), 2.14–2.07 (m, 2H), 1.71–1.60 (m, 3H), 1.55 (s, 3H), 1.50–1.39 (m, 7H), 1.34 (s, 3H). ^{13}C NMR (126 MHz, $CDCl_3$) δ 109.4, 109.3, 100.9, 71.2, 70.8, 70.2, 70.2, 61.5, 48.0, 47.9, 27.0, 27.0, 26.7, 26.0, 25.4, 24.2, 19.5. HRMS-ESI$^+$ (m/z): $[M+H]^+$ calcd. for $C_{17}H_{27}NO_8S$, 406.1530; found, 406.1523.

C. Reaction optimization studies

C.1 Optimization of aliphatic olefin aziridination

Table S1. Evaluation of the impact of HFIP loadings on aziridination. Reactions were carried out using 0.050 mmol of **1a** following Procedure A in dry CH_2Cl_2 (1.0 mL). Yields were determined by ^1H NMR analysis of the reaction mixture with triethyl 1,3,5-benzenetricarboxylate as the internal standard. The optimal condition is highlighted in red.

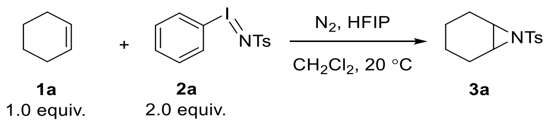

Entry	HFIP loading	NMR yield
1	0.0 equiv	10%
2	2.0 equiv	24%
3	10.0 equiv	38%
4	as solvent	63%

Table S2. Evaluation of the impact of temperature on aziridination. Reactions were carried out using 0.050 mmol of **1a** following Procedure A in dry HFIP (1.0 mL). Yields were determined by ^{1}H NMR analysis of the reaction mixture with triethyl 1,3,5-benzenetricarboxylate as the internal standard. The optimal condition is highlighted in red.

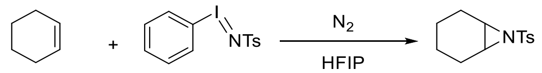

1a 1.0 equiv. **2a** 2.0 equiv. **3a**

Entry	Temperature	NMR yield
1	20 °C	63%
2	30 °C	50%
3	40 °C	48%
4	50 °C	43%

Table S3. Evaluation of the impact of concentration and scale on aziridination. Reactions were carried out using **1a** as substrate following Procedure A in dry HFIP. Yields were determined by ^{1}H NMR analysis of the reaction mixture with triethyl 1,3,5-benzenetricarboxylate as the internal standard. The optimal condition is highlighted in red.

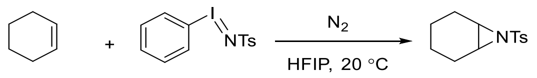

1a 1.0 equiv. **2a** 2.0 equiv. **3a**

Entry	1a loading	1a concentration	NMR yield
1	0.050 mmol	0.050 M	63%
2	0.200 mmol	0.200 M	67%

Table S4. Evaluation of the impact of iminoiodinane loading on aziridination. Reactions were carried out using 0.200 mmol of **1a** following Procedure A in dry HFIP (1.0 mL). Yields were determined by ^{1}H NMR analysis of the reaction mixture with triethyl 1,3,5-benzenetricarboxylate as the internal standard. The optimal condition is highlighted in red.

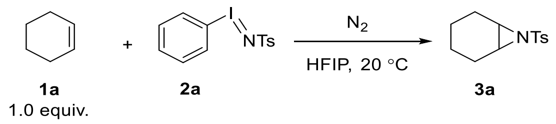

Entry	2a loading	NMR yield
1	1.0 equiv	22%
2	1.5 equiv	28%
3	2.0 equiv	67%

Table S5. Evaluation of the impact of fluorinated solvent on aziridination. Reactions were carried out using 0.200 mmol of **1a** following Procedure A in dry solvent (1.0 mL). Yields were determined by ^{1}H NMR analysis of the reaction mixture with triethyl 1,3,5-benzenetricarboxylate as the internal standard. The optimal condition is highlighted in red.

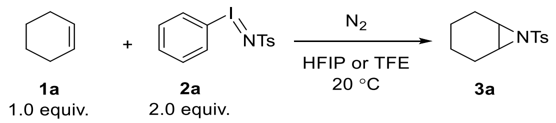

Entry	Solvent	NMR yield
1	TFE	16%
2	HFIP	67%

Table S6. Evaluation of alternative Lewis acids on aziridination. Reactions were carried out using 0.200 mmol of **1a** following Procedure A with 1.0 mmol of additives in dry CH_2Cl_2 (1.0 mL). Yields were determined by ^1H NMR analysis of the reaction mixture with triethyl 1,3,5-benzenetricarboxylate as the internal standard.

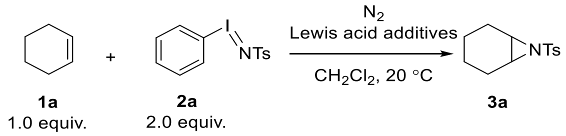

Entry	Additive	NMR yield
1	$BF_3{\cdot}OEt_2$	0%
2	TfOH	0%
3	$Zn(OTf)_2$	0%

Table S7. Evaluation of the impact of generating 2a *in situ* on aziridination. Reactions were carried out using 0.200 mmol of **1a** following Procedure A varying loading of **5** in dry HFIP (1.0 mL). Yields were determined by ^{1}H NMR analysis of the reaction mixture with triethyl 1,3,5-benzenetricarboxylate as the internal standard.

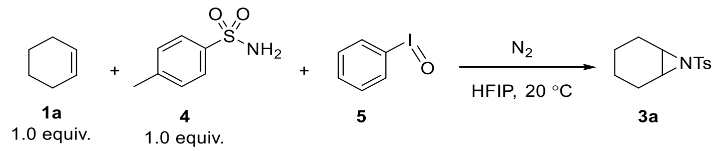

Entry	Loading of 5	NMR yield
1	1.0 equiv	19%
2	2.0 equiv	27%

Table S8. Evaluation of the impact of light on aziridination. Reactions were carried out using 0.200 mmol of **1a** following Procedure A in dry HFIP (1.0 mL). The reactions are carried out in the dark or under ambient lighting. Yields were determined by ^{1}H NMR analysis of the reaction mixture with triethyl 1,3,5-benzenetricarboxylate as the internal standard.

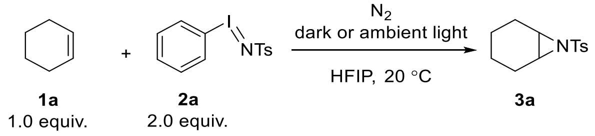

Entry	Condition	NMR yield
1	dark	64%
2	ambient light	67%

C.2 Challenging reaction partners

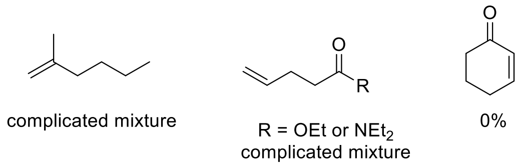

Figure S1. 1,1-Disubstituted olefins, those containing α-carbonyl C–H bonds, and electron-poor olefins suffer from decomposition or low yields during aziridination.

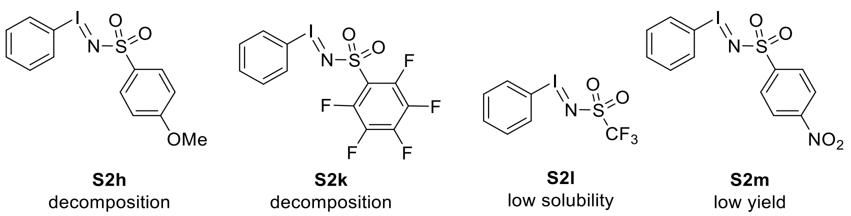

Figure S2. Aziridination utilizing unstable or very electron poor iminoiodinanes suffers from decomposition, low solubility in HFIP, or low reactivity during aziridination.

D. Evaluation of aziridination mechanism

Speciation of PhINTs in HFIP

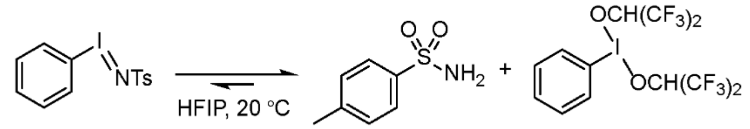

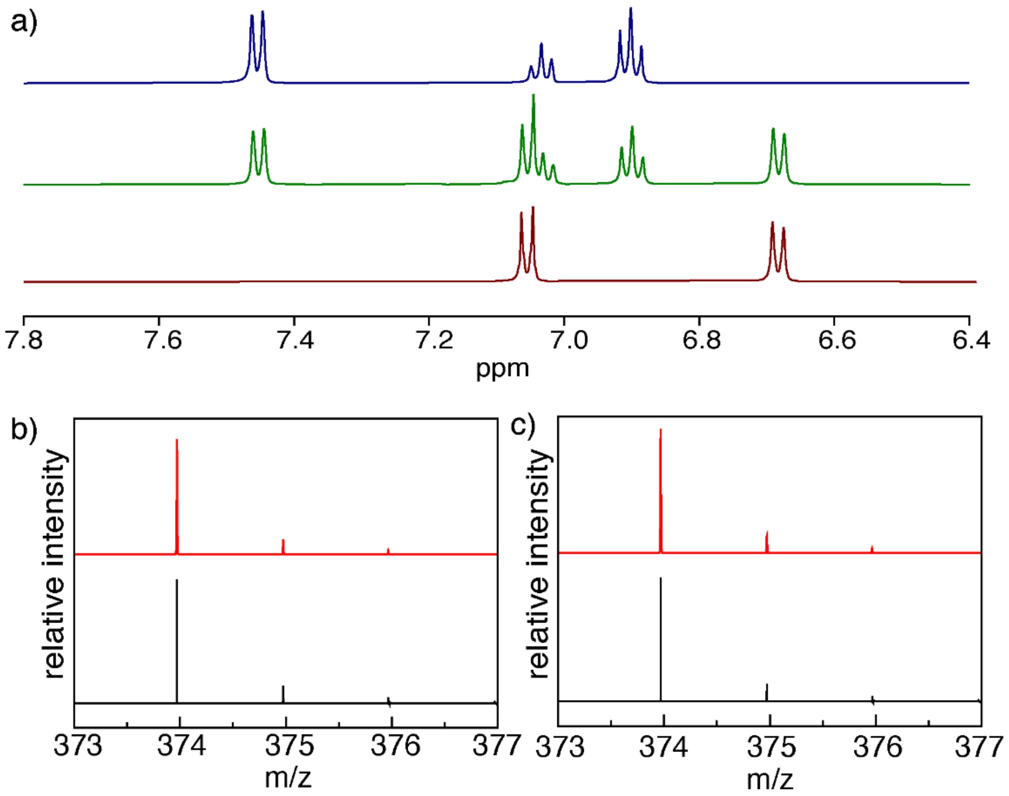

Figure S3. NMR and mass spectroscopic experiments demonstrating PhINTs (2a) undergoing reaction with HFIP to reversibly afford ArI(OCH(CF_3)$_2$)$_2$ and $TsNH_2$. a) ^1H NMR spectra (externally referenced to CD_3CN via a capillary) of PhIO in HFIP (blue line —), PhINTs in HFIP (green line —), and $TsNH_2$ in HFIP (red line —), b) Mass spectrometry analysis of a sample of 50 mM PhINTs in HFIP showing the presence of PhINTs (HRMS-ESI$^+$ (m/z): [M+H]$^+$ calcd. for $C_{13}H_{13}INO_2S^+$, 373.9706; found, 373.9701), and c) Mass spectrometry analysis of a sample of 50 mM PhIO and 50 mM $TsNH_2$ in HFIP showing the presence of PhINTs (HRMS-ESI$^+$ (m/z): [M+H]$^+$ calcd. for $C_{13}H_{13}INO_2S^+$, 373.9706; found, 373.9701).

Mass Balance in the Aziridination of Cyclohexene

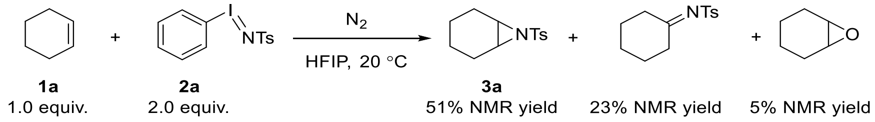

Figure S4. Mass balance in the aziridination of cyclohexene. Under an N_2 atmosphere, a 20-mL scintillation vial was charged with cyclohexene (**1a**, 20.2 μL, 0.200 mmol, 1.00 equiv) and dry HFIP (1 mL). To this vial was added PhINTs (149 mg, 0.400 mmol, 2.00 equiv), and the reaction was stirred for 16 h at 20 °C. Triethyl 1,3,5-benzenetricarboxylate was then added as an internal standard; an aliquot of the reaction mixture was then taken, diluted with CD_3CN, and subjected to ^1H NMR analysis. The reaction afforded 51% yield of aziridine product **3a** in, 23% imine, 5% cyclohexene oxide, and 3% unreacted cyclohexene.

Control Reactions Using PhIO to Test for Epoxidation

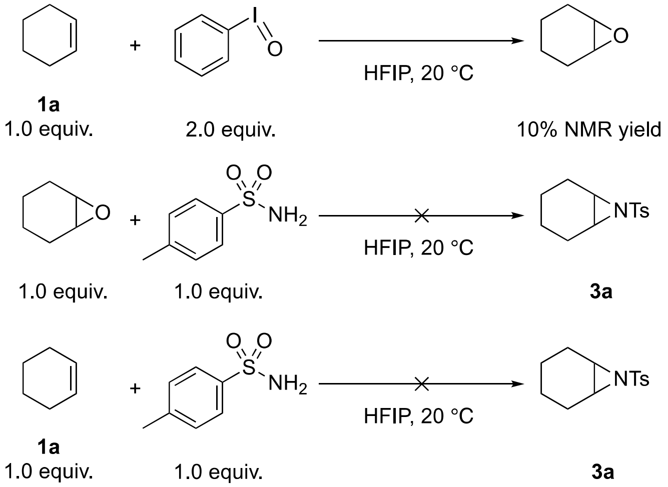

Figure S5. Control reactions using PhIO to test for epoxidation. a) Under an N_2 atmosphere, a 20-mL scintillation vial was charged with cyclohexene (**1a**, 20.2 µL, 0.200 mmol, 1.00 equiv) and dry HFIP (1 mL). To this vial was added PhIO (44.0 mg, 0.400 mmol, 2.00 equiv), and the reaction was stirred for 16 h at 20 °C. An aliquot was then taken, diluted with $CDCl_3$, and subjected to ^1H NMR analysis. Cyclohexene oxide was formed in <10% NMR yield using triethyl 1,3,5-benzenetricarboxylate as the internal standard. b) Under an N_2 atmosphere, a 20-mL scintillation vial was charged with cyclohexene (**1a**, 20.2 µL, 0.200 mmol, 1.00 equiv) and dry HFIP (1 mL). To this vial was added $TsNH_2$ (34.2 mg, 0.200 mmol, 1.00 equiv), and the reaction was stirred for 16 h at 20 °C. An aliquot was then taken, diluted with $CDCl_3$, and subjected to ^1H NMR analysis. The aziridine product **3a** was not observed. c) Under an N_2 atmosphere, a 20-mL scintillation vial was charged with cyclohexene oxide (24.2 µL, 0.200 mmol, 1.00 equiv) and dry HFIP (1 mL). To this vial was added $TsNH_2$ (34.2 mg, 0.200 mmol, 1.00 equiv), and the reaction was stirred for 16 h at 20 °C. An aliquot was then taken, diluted with $CDCl_3$, and subjected to ^1H NMR analysis. The aziridine product **3a** was not observed.

Aziridination of Cyclopentene in the Presence of PBN

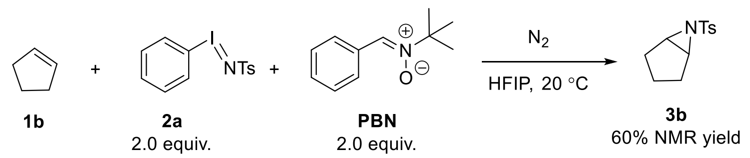

Figure S6. Aziridination of Cyclopentene in the Presence of PBN. Under an N_2 atmosphere, a 20-mL scintillation vial was charged with cyclopentene (**1b**, 17.7 µL, 0.200 mmol, 1.00 equiv), PBN (70.8 mg, 0.400 mmol, 2.00 equiv), and dry HFIP (1 mL). To this vial was added PhINTs (**2a**, 149 mg, 0.400 mmol, 2.00 equiv), and the reaction was stirred for 16 h at 20 °C. An aliquot was then taken, diluted with $CDCl_3$, and subjected to ^1H NMR analysis. The aziridine product **3b** was formed in 60% NMR yield; unreacted PBN was observed (quantitative).

Aziridination of cis- and trans-β-methylstyrene

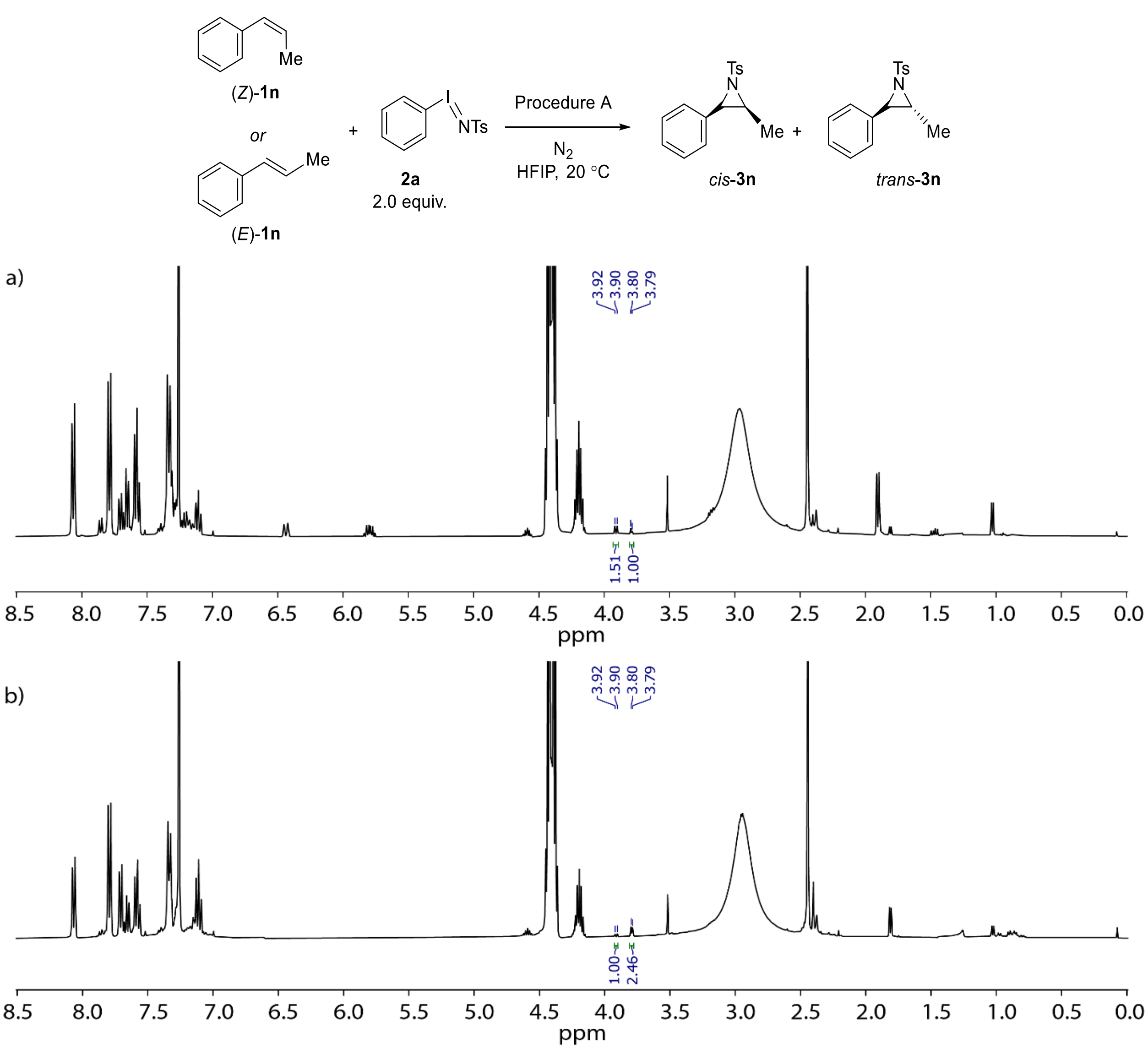

Figure S7. Determination of diastereomeric distribution in the product in the aziridination of *cis*- or *trans*-β-methylstyrene. The reactions were carried out according to Procedure A using 0.200 mmol of styrene. An aliquot was then taken, diluted with $CDCl_3$, and subjected to ^1H NMR analysis to determine the ratio of the *cis*- and *trans*-aziridine product **3n** via the integrations at 3.91 ppm (d, *J* = 7.4 Hz, 1H, *cis*-**3n**) and 3.79 ppm (d, *J* = 4.8 Hz, 1H, *trans*-**3n**), respectively. a) Reaction using *cis*-β-methylstyrene afforded aziridine product **3n** as a diastereomeric mixture of 1.5:1.0 *c*:*t*, and b) Reaction using *trans*-β-methylstyrene afforded aziridine product **3n** as a diastereomeric mixture of 1.0:2.5 *c*:*t*.

Control reaction of cis- and trans-β-methylstyrene in the absence of PhINTs

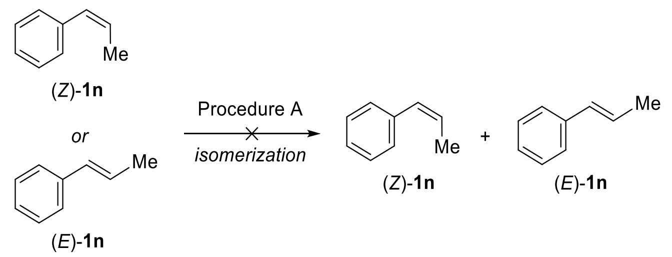

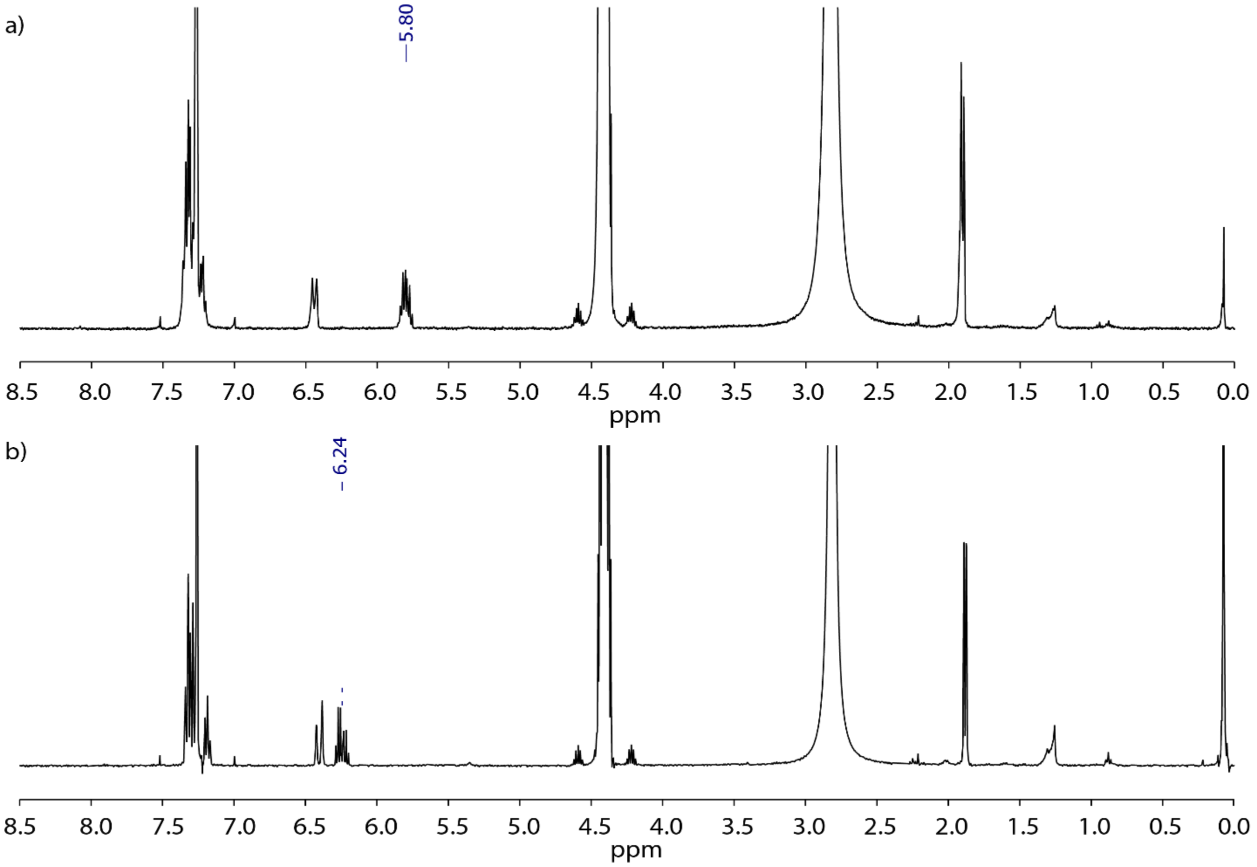

Figure S8. Control reaction to check for isomerization of *cis-* and *trans-β-*methylstyrene in HFIP. The reactions were carried out according to Procedure A using 0.200 mmol of styrene in the absence of PhINTs (**2a**) to check for substrate isomerization in HFIP. After the reaction, an aliquot was then taken, diluted with $CDCl_3$, and subjected to ^1H NMR analysis to determine the amount of the *cis-* or *trans-* β -methylstyrene by analyzing the signals at 5.80 ppm (m, 1H, *cis*-β-methylstyrene) and 6.24 ppm (m, 1H, *trans*-β-methylstyrene). In both the a) reaction with *cis*-β-methylstyrene and b) reaction with *trans*-β-methylstyrene, no isomerization was observed.

E. NMR Spectra for new compounds

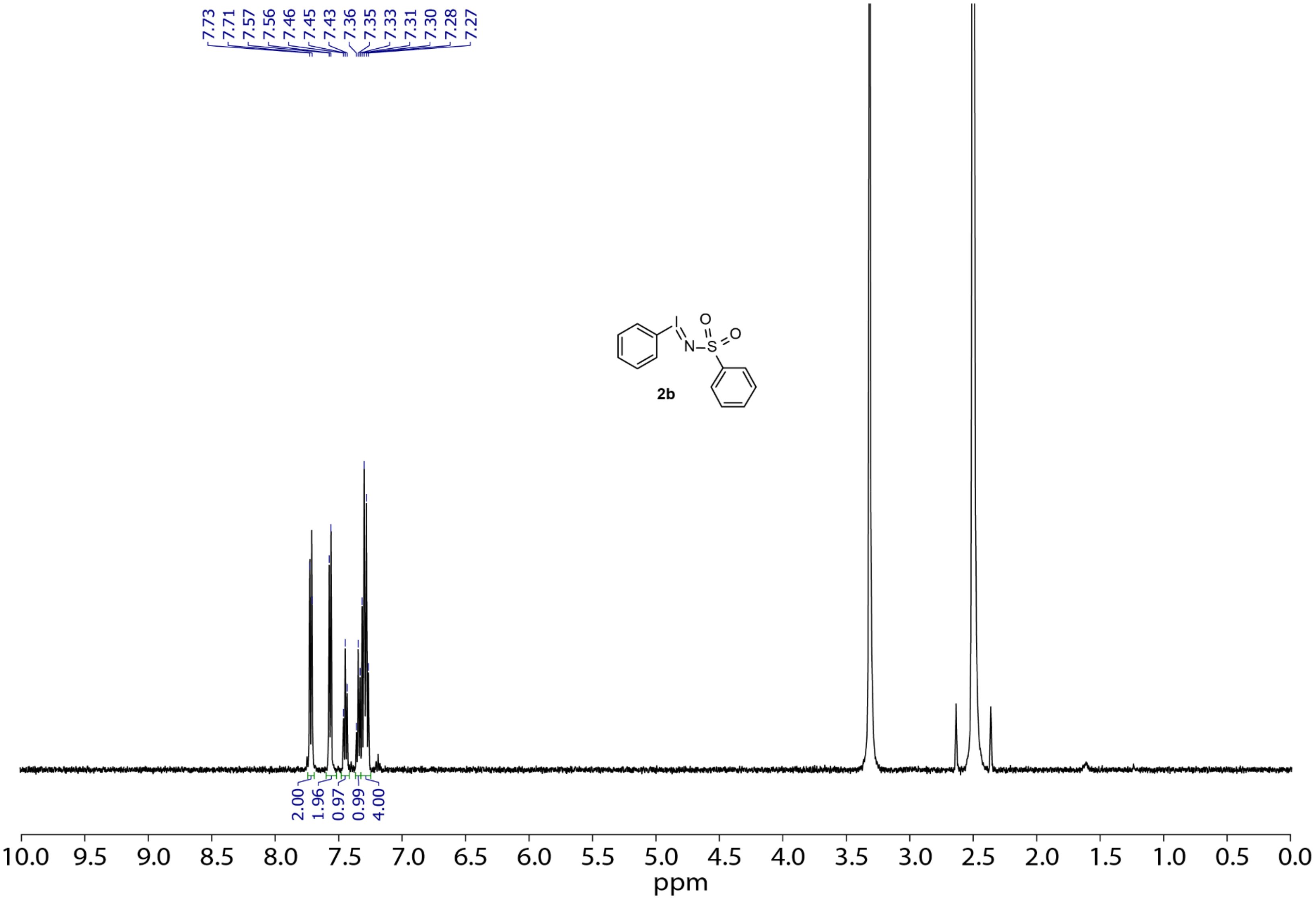

Figure S9. ^{1}H NMR spectrum of *N*-(phenyl-λ^3-iodaneylidene)benzenesulfonamide (**2b**) in $(CD_3)_2SO$ (400 MHz) at 23 °C.

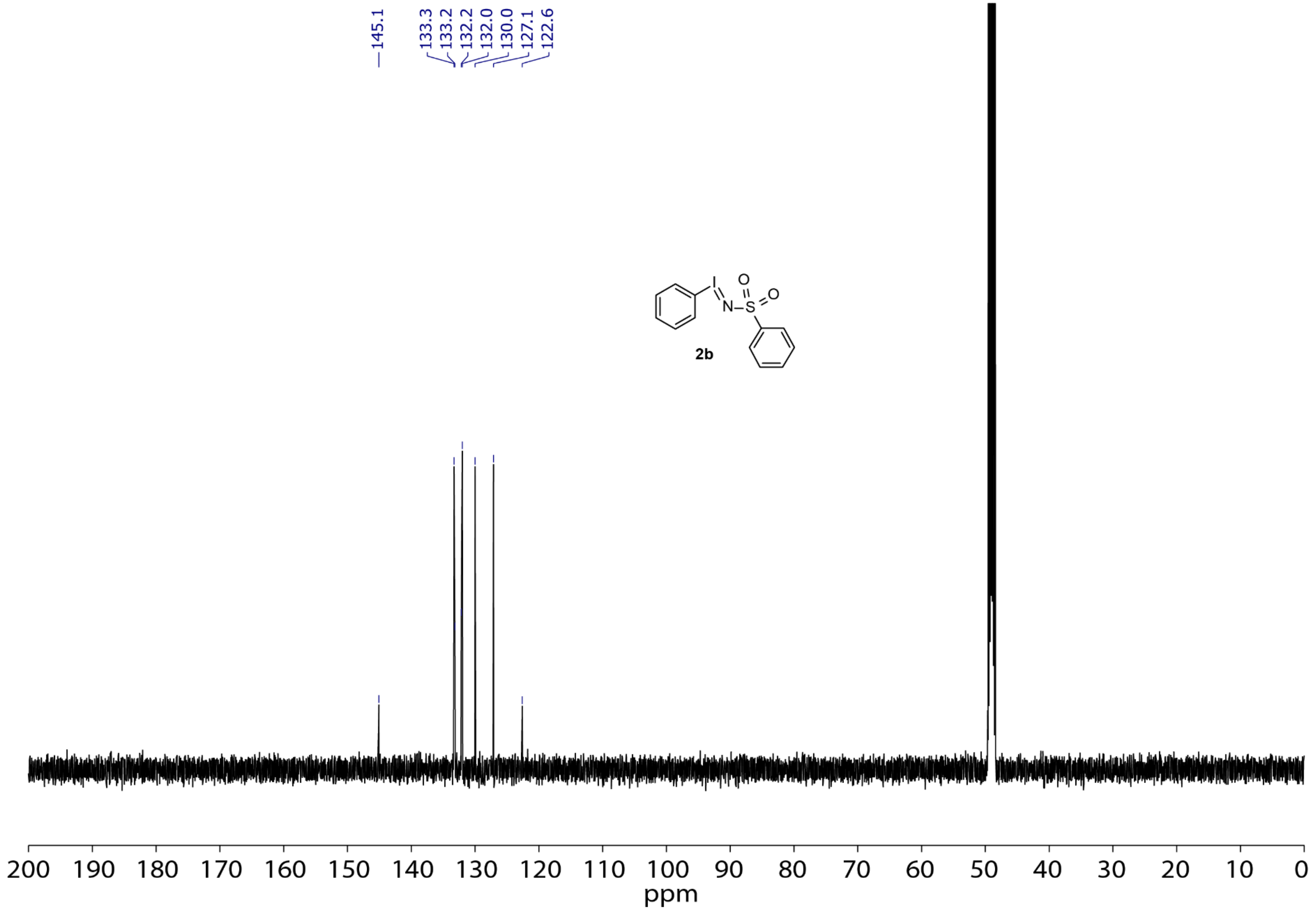

Figure S10. ^{13}C NMR spectrum of *N*-(phenyl-λ^3-iodaneylidene)benzenesulfonamide (**2b**) in CD_3OD (126 MHz) at 23 °C.

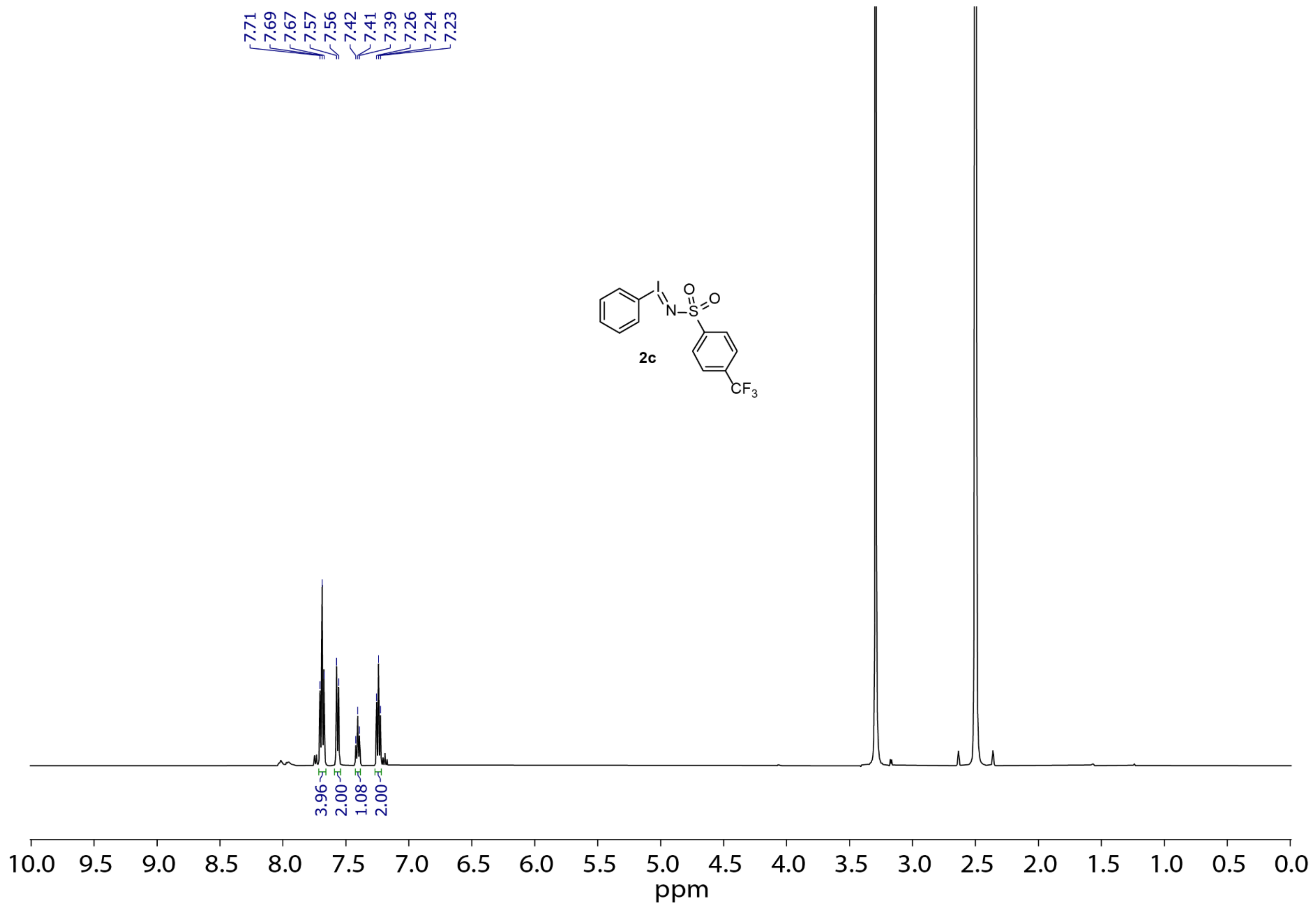

Figure S11. ^{1}H NMR spectrum of *N*-(phenyl-λ^{3}-iodaneylidene)-4-(trifluoromethyl)benzenesulfonamide (**2c**) in $(CD_3)_2SO$ (400 MHz) at 23 °C.

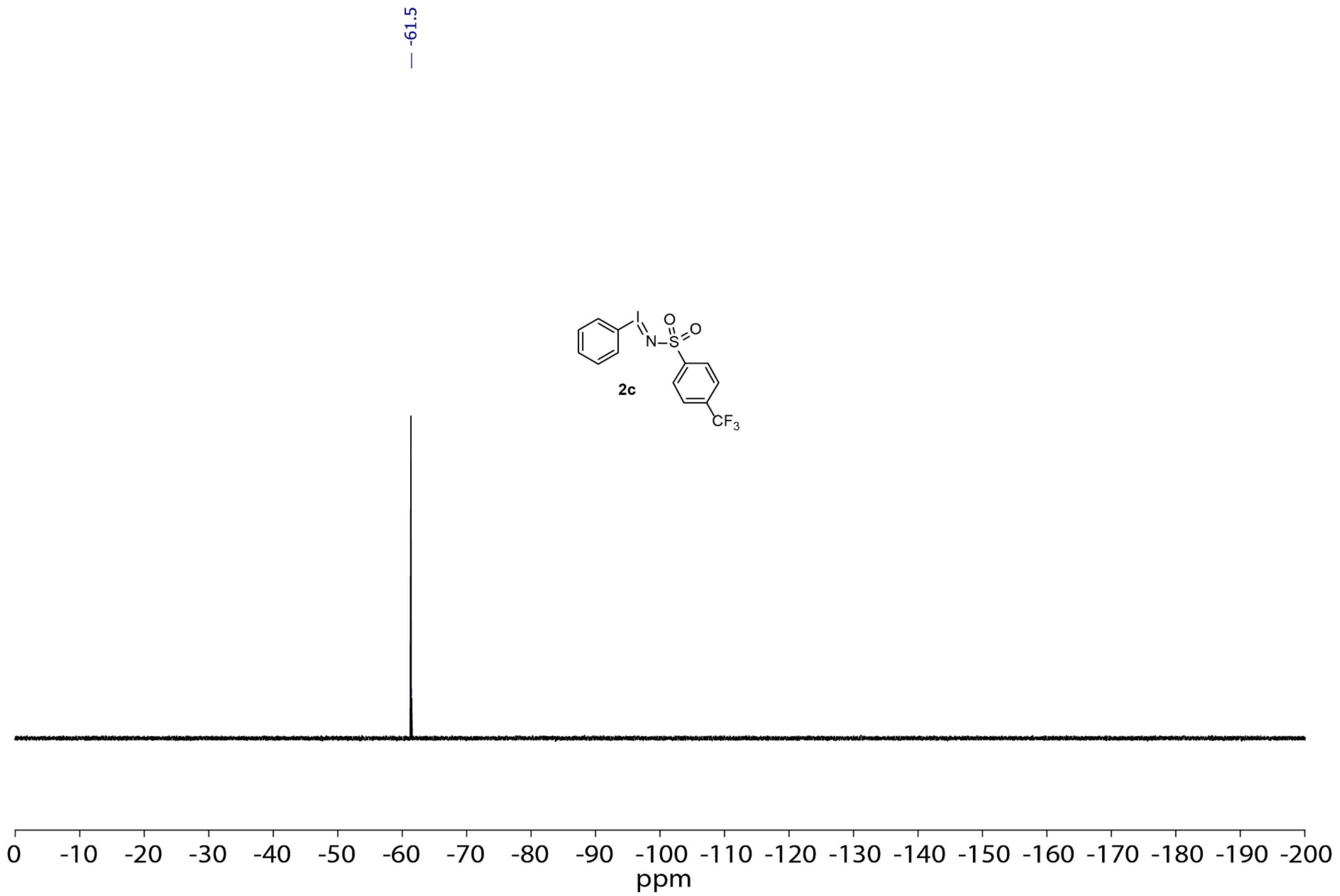

Figure S12. ^{19}F NMR spectrum of *N*-(phenyl-λ^3-iodaneylidene)-4-(trifluoromethyl)benzenesulfonamide (**2c**) in $(CD_3)_2SO$ (377 MHz) at 23 °C.

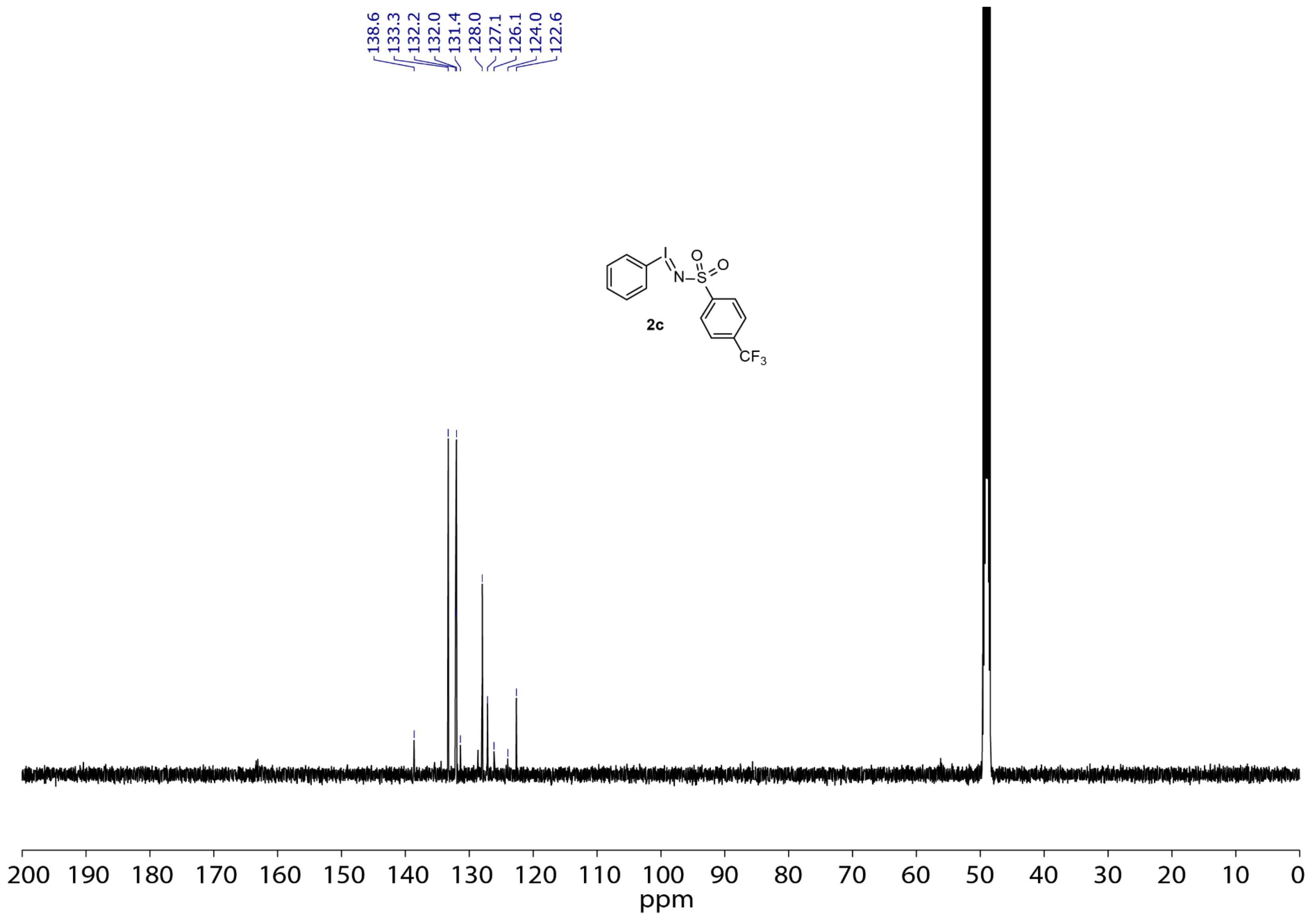

Figure S13. ^{13}C NMR spectrum of *N*-(phenyl-λ^3-iodaneylidene)-4-(trifluoromethyl)benzenesulfonamide (**2c**) in CD_3OD (126 MHz) at 23 °C.

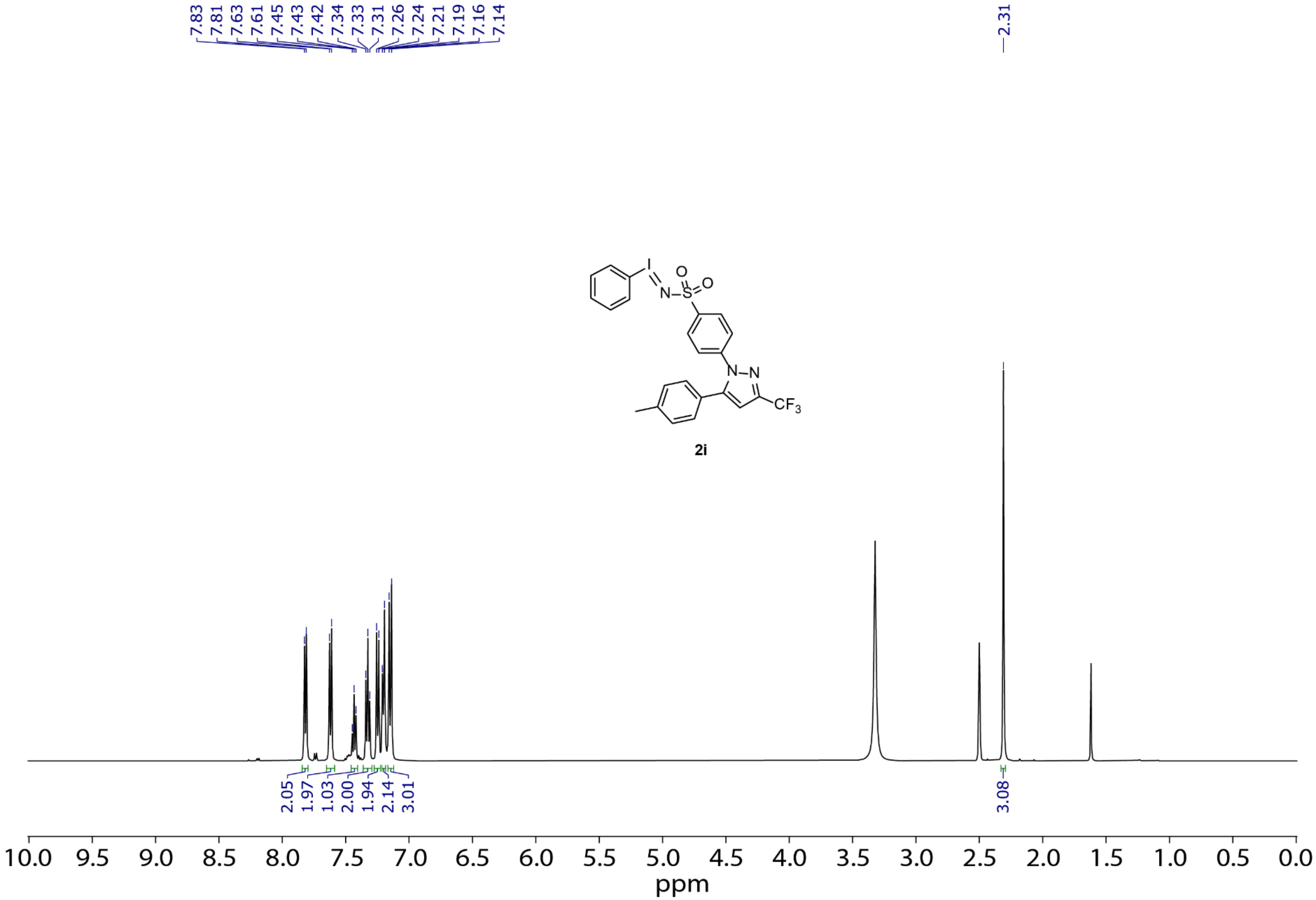

Figure S14. ^{1}H NMR spectrum of *N*-(phenyl-λ^{3}-iodaneylidene)-4-(5-(*p*-tolyl)-3-(trifluoromethyl)-1*H*-pyrazol-1-yl)benzenesulfonamide (**2i**) in $CDCl_3$ (400 MHz) at 23 °C.

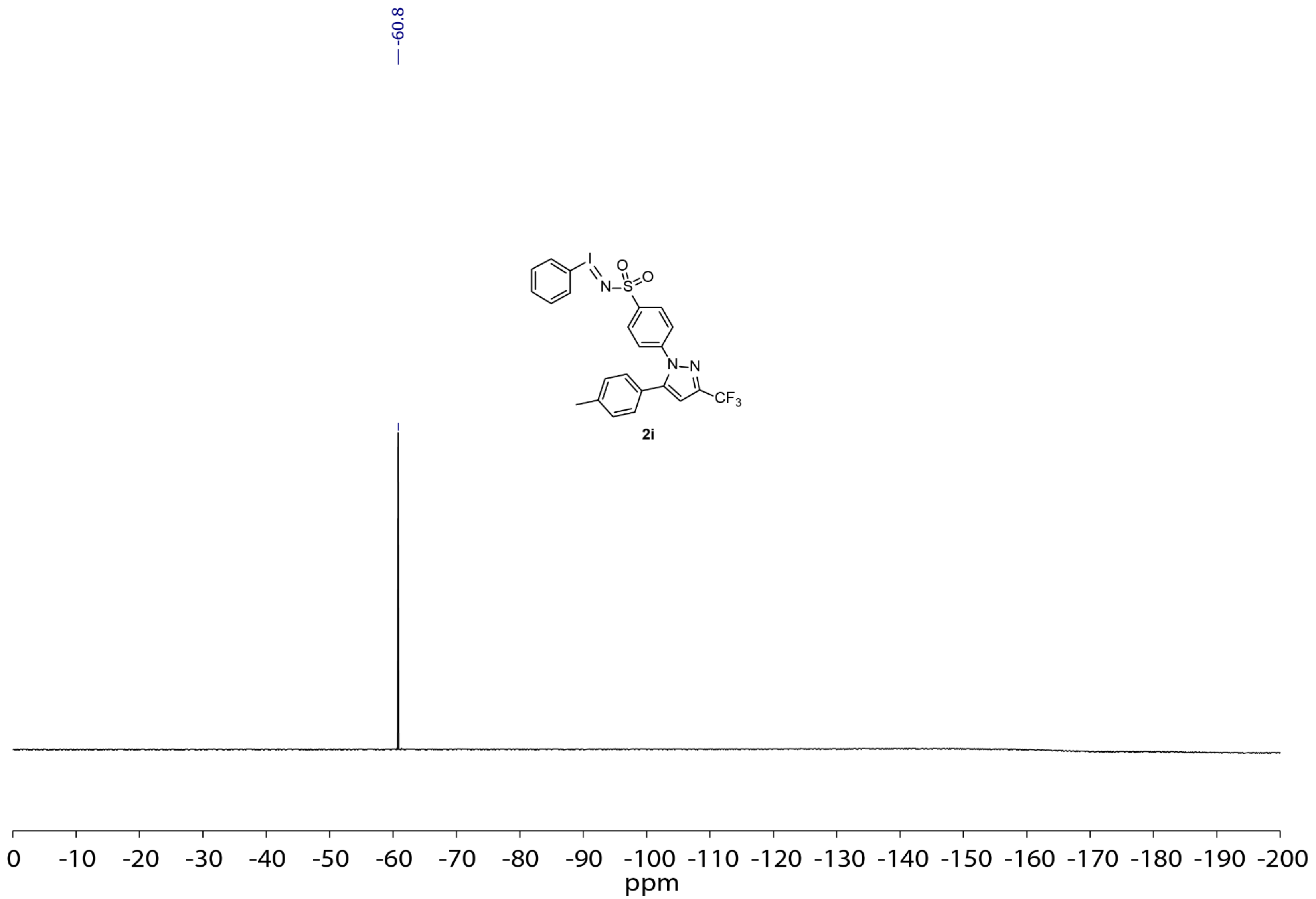

Figure S15. ^{19}F NMR spectrum of *N*-(phenyl-λ^3-iodaneylidene)-4-(5-(*p*-tolyl)-3-(trifluoromethyl)-1*H*-pyrazol-1-yl)benzenesulfonamide (**2i**) in $CDCl_3$ (377 MHz) at 23 °C.

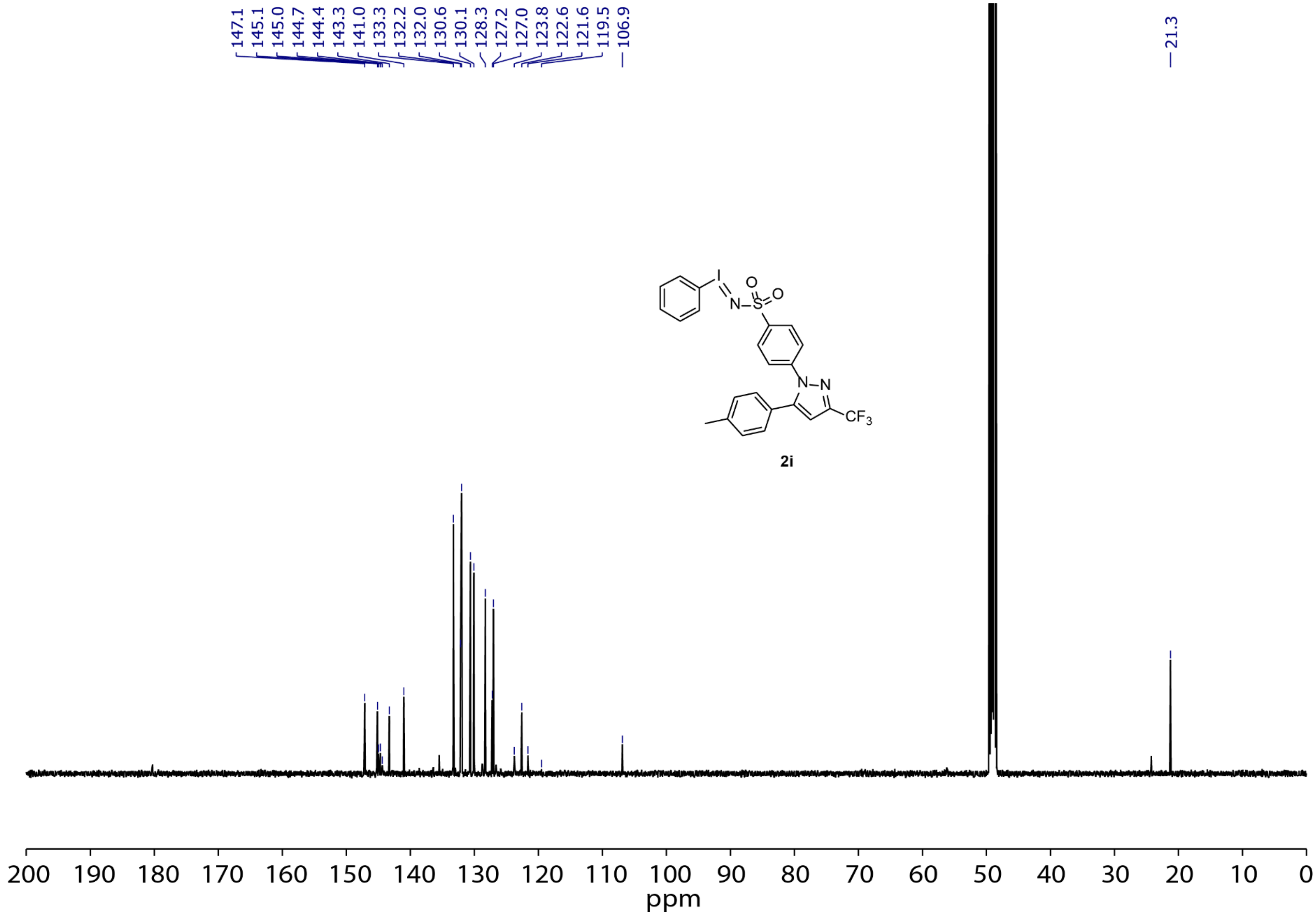

Figure S16. ^{13}C NMR spectrum of *N*-(phenyl-λ^3-iodaneylidene)-4-(5-(*p*-tolyl)-3-(trifluoromethyl)-1*H*-pyrazol-1-yl)benzenesulfonamide (**2i**) in CD_3OD (126 MHz) at 23 °C. Extra peaks are attributed to celecoxib.

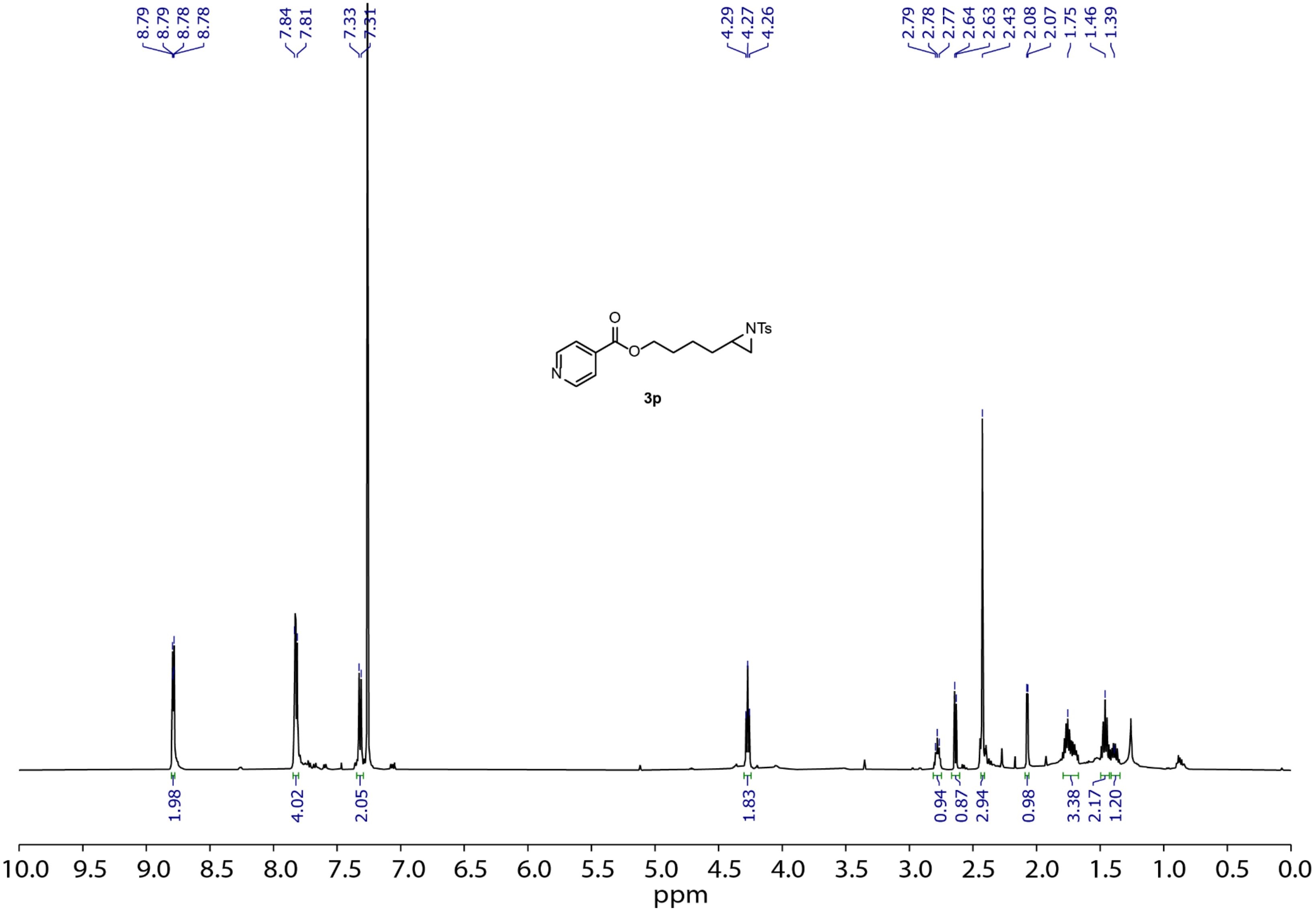

Figure S17. ^{1}H NMR spectrum of 4-(1-tosylaziridin-2-yl)butyl isonicotinate (**3p**) in $CDCl_3$ (400 MHz) at 23 °C.

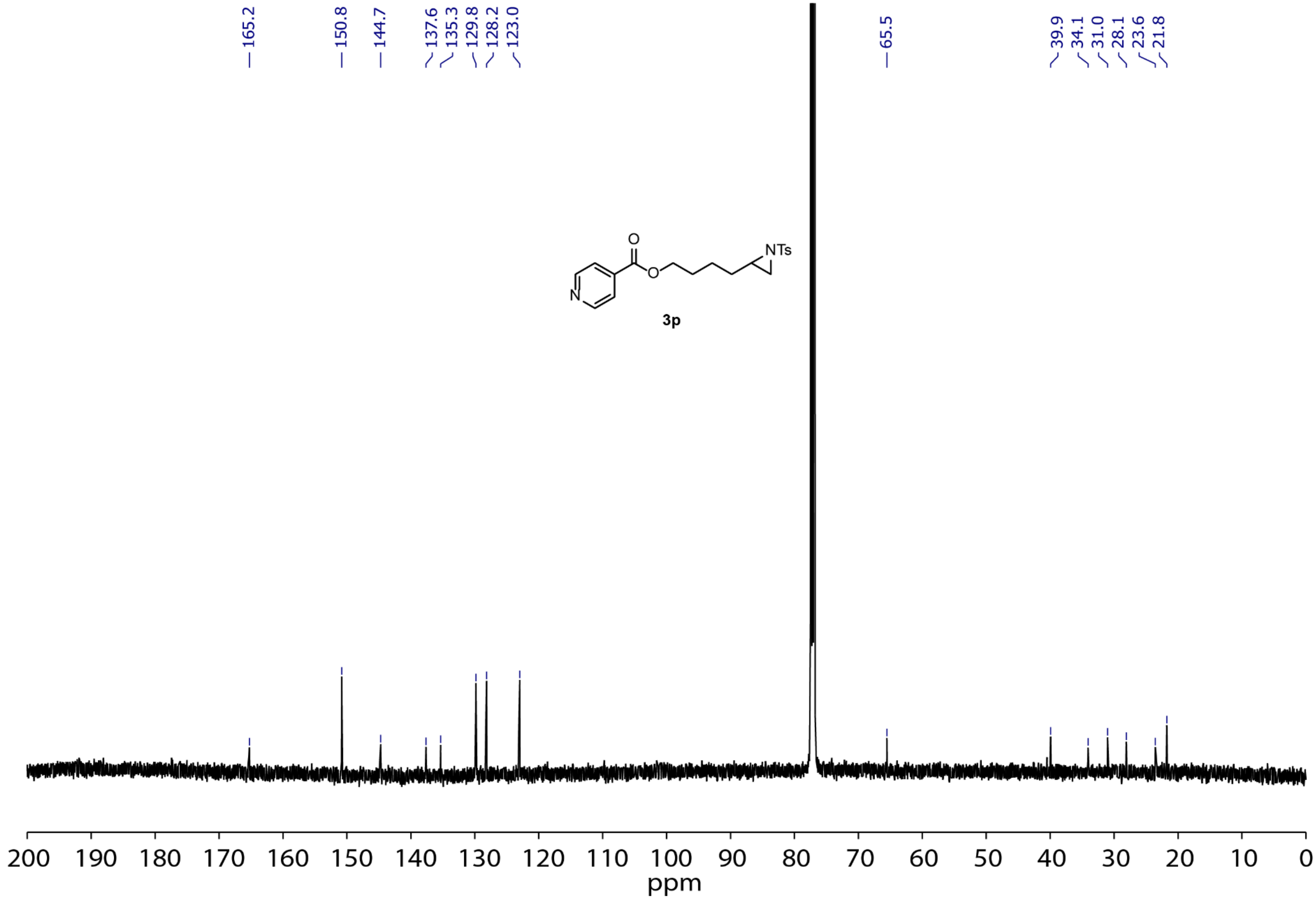

Figure S18. ^{13}C NMR spectrum of 4-(1-tosylaziridin-2-yl)butyl isonicotinate (**3p**) in $CDCl_3$ (126 MHz) at 23 °C.

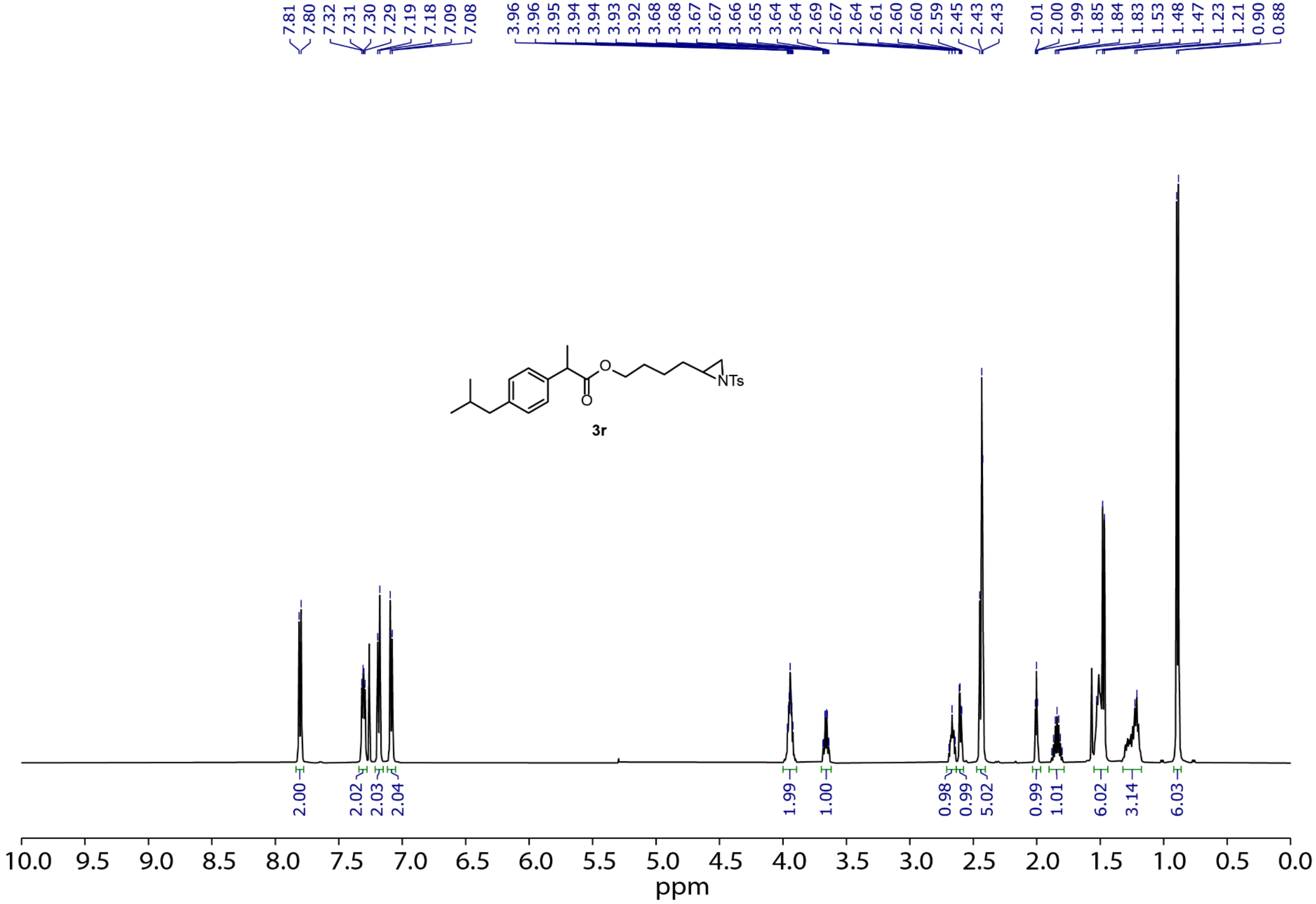

Figure S19. ^{1}H NMR spectrum of 4-(1-tosylaziridin-2-yl)butyl 2-(4-isobutylphenyl)propanoate (**3r**) in $CDCl_3$ (400 MHz) at 23 °C.

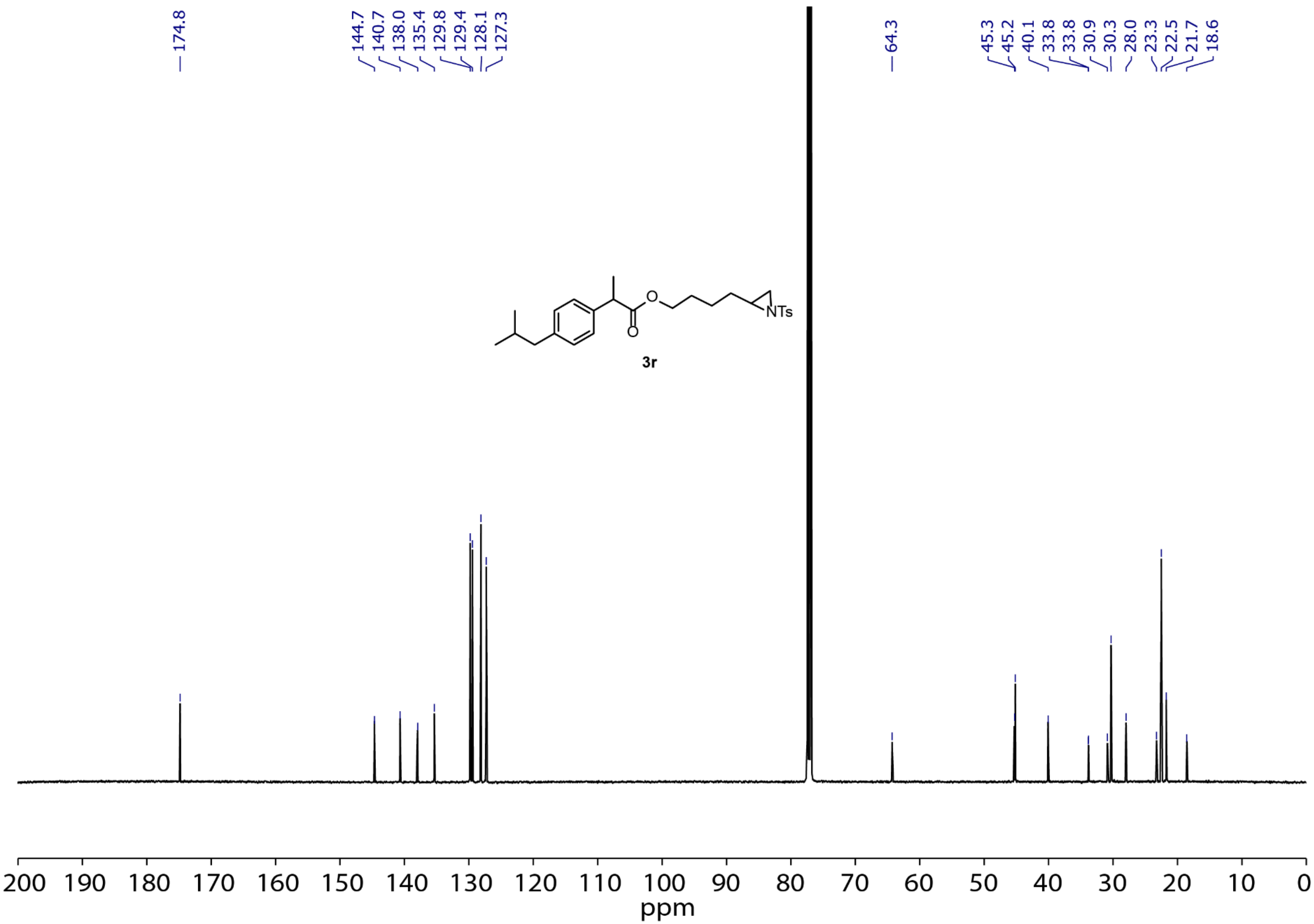

Figure S20. ^{13}C NMR spectrum of 4-(1-tosylaziridin-2-yl)butyl 2-(4-isobutylphenyl)propanoate (**3r**) in $CDCl_3$ (126 MHz) at 23 °C.

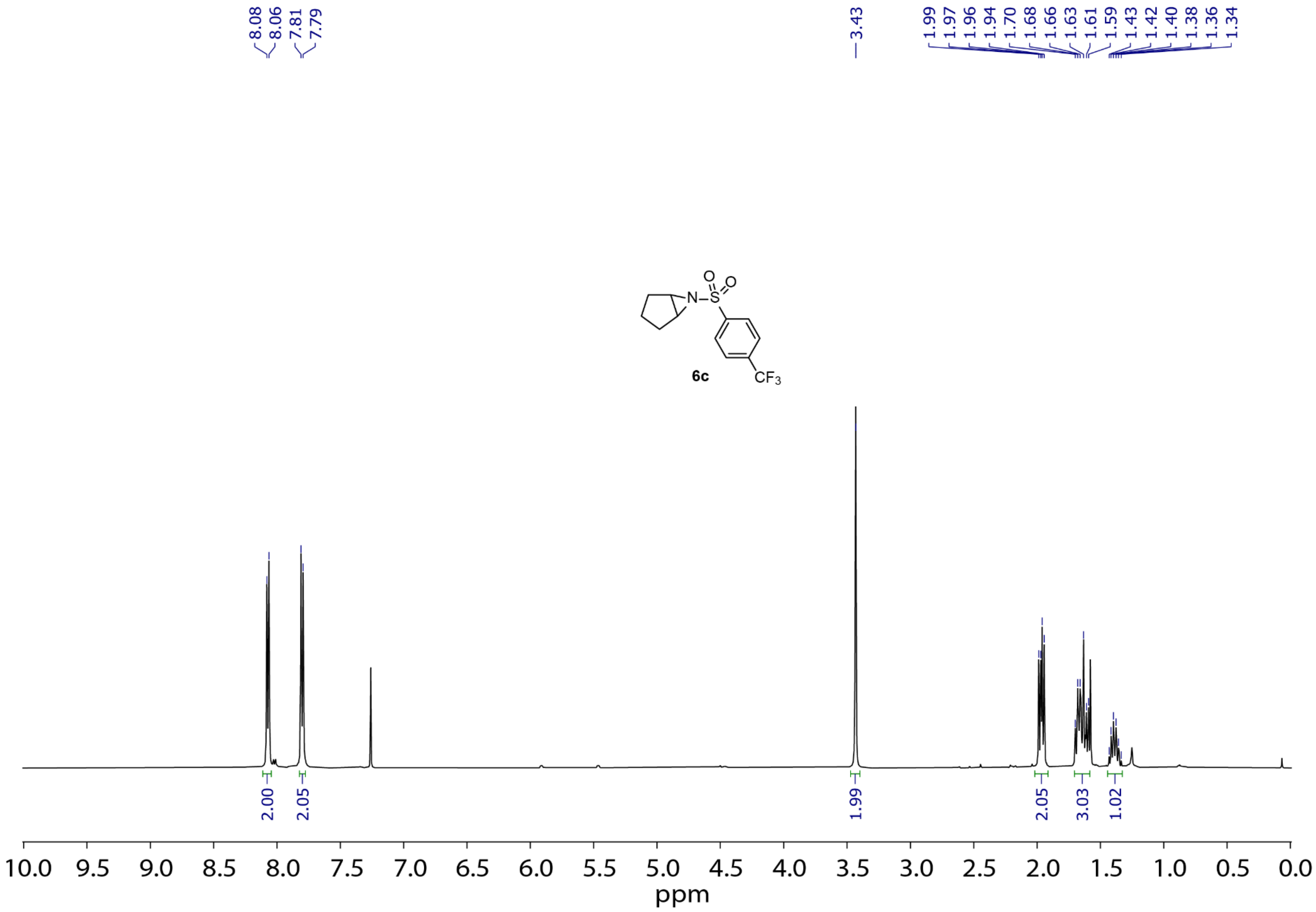

Figure S21. ^{1}H NMR spectrum of 6-((4-(trifluoromethyl)phenyl)sulfonyl)-6-azabicyclo[3.1.0]hexane (**6c**) in $CDCl_3$ (400 MHz) at 23 °C.

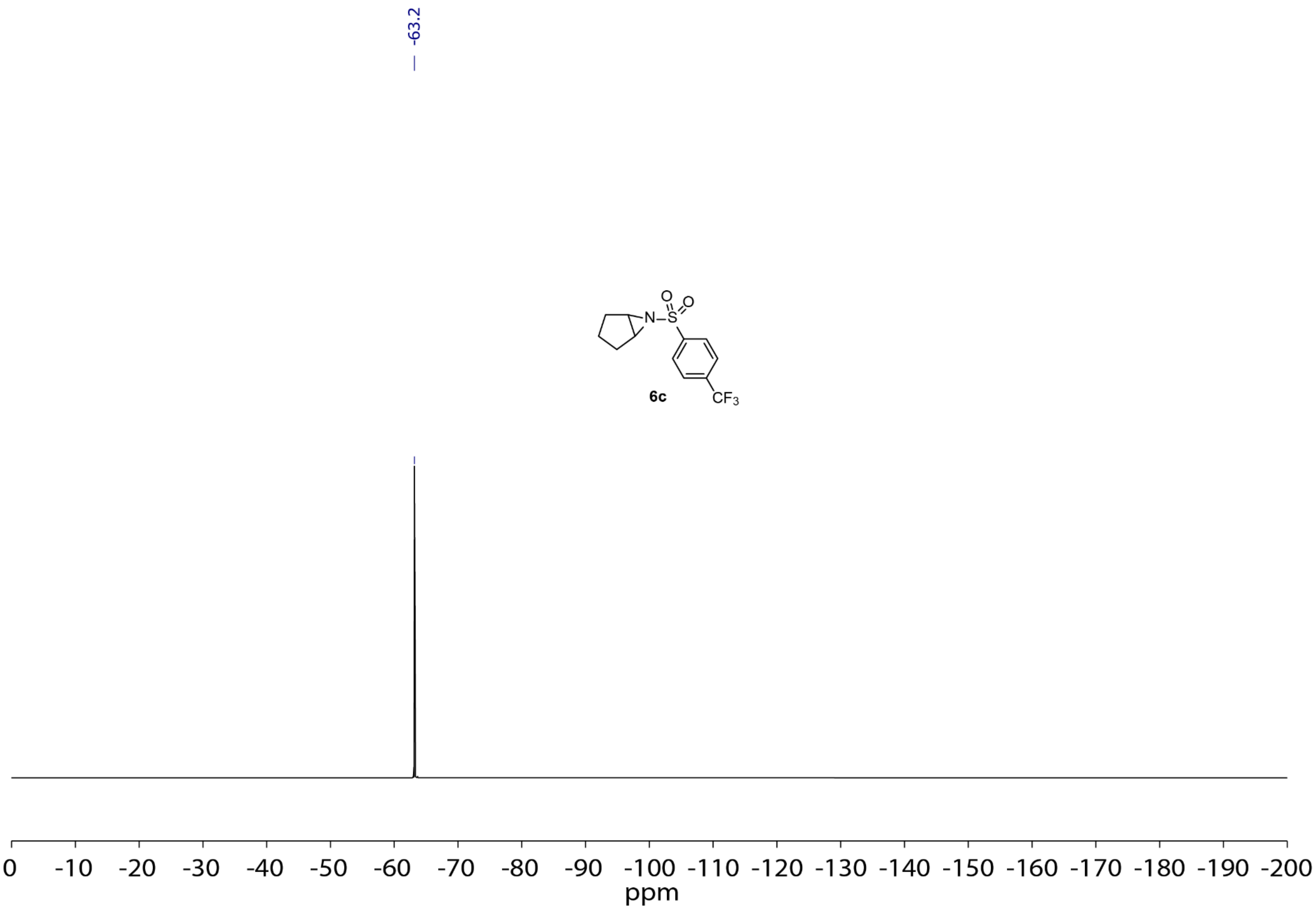

Figure S22. ^{19}F NMR spectrum of 6-((4-(trifluoromethyl)phenyl)sulfonyl)-6-azabicyclo[3.1.0]hexane (**6c**) in $CDCl_3$ (377 MHz) at 23 °C.

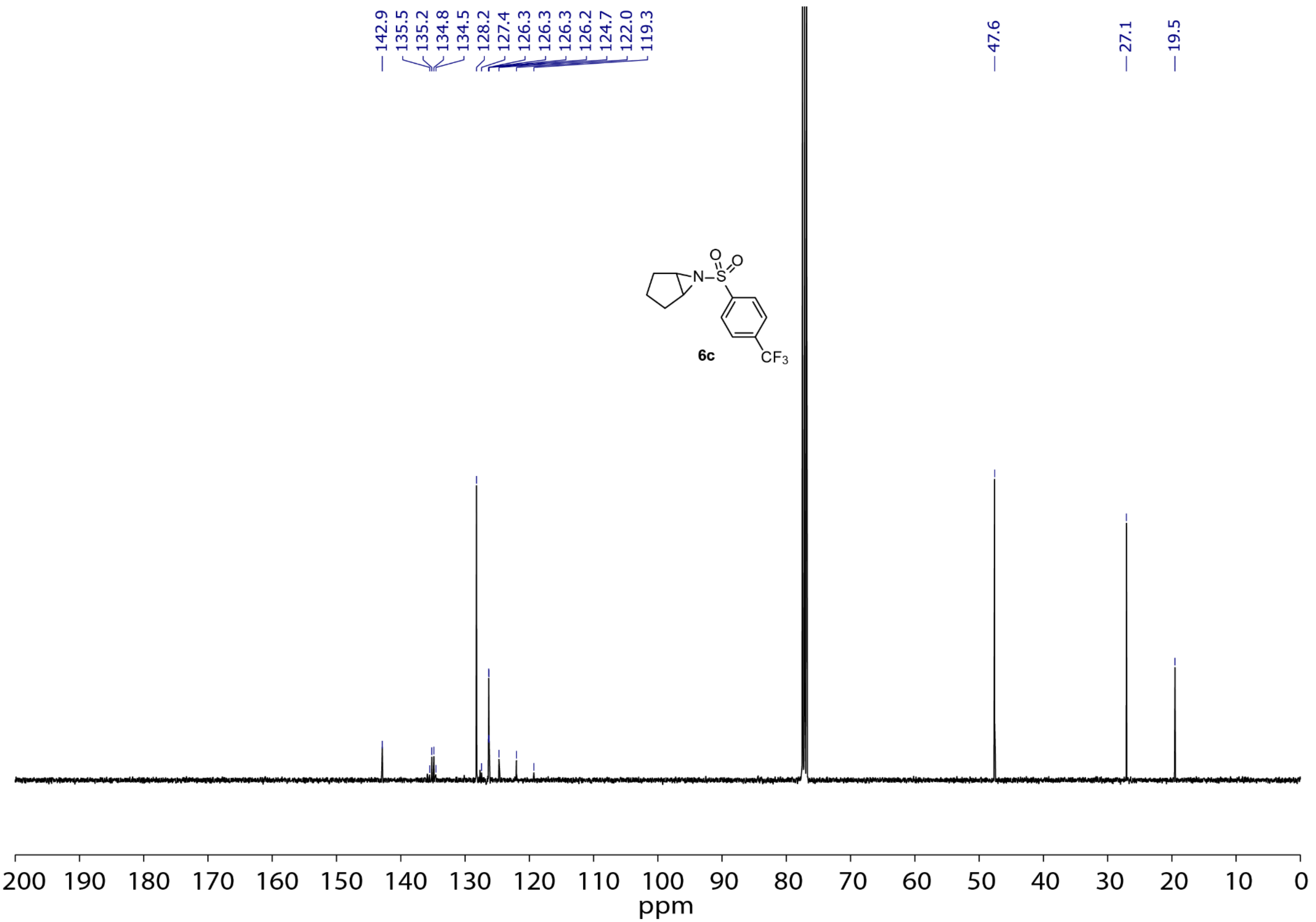

Figure S23. ^{13}C NMR spectrum of 6-((4-(trifluoromethyl)phenyl)sulfonyl)-6-azabicyclo[3.1.0]hexane (**6c**) in $CDCl_3$ (126 MHz) at 23 °C.

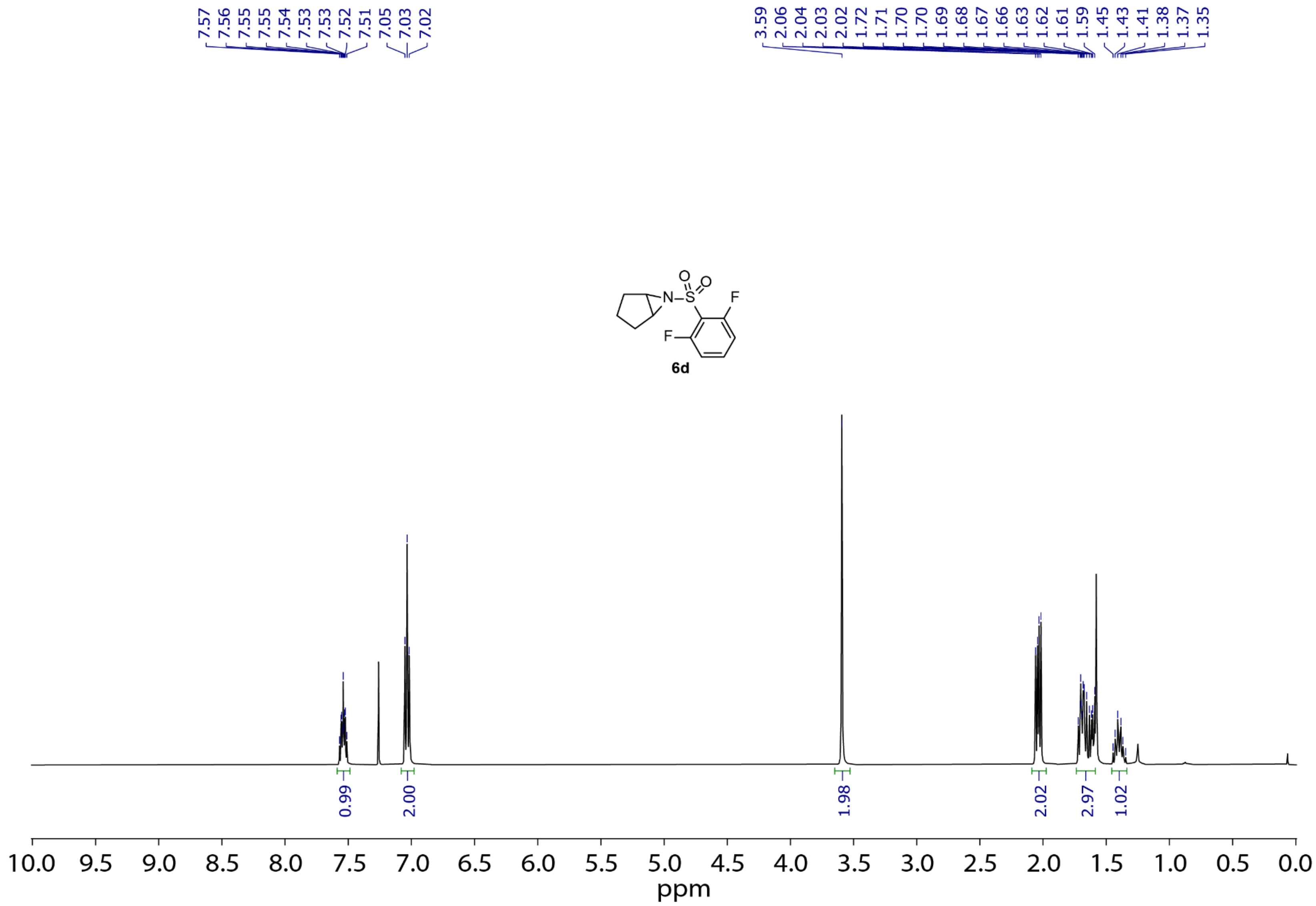

Figure S24. ^{1}H NMR spectrum of 6-((2,6-difluorophenyl)sulfonyl)-6-azabicyclo[3.1.0]hexane (**6d**) in $CDCl_3$ (400 MHz) at 23 °C.

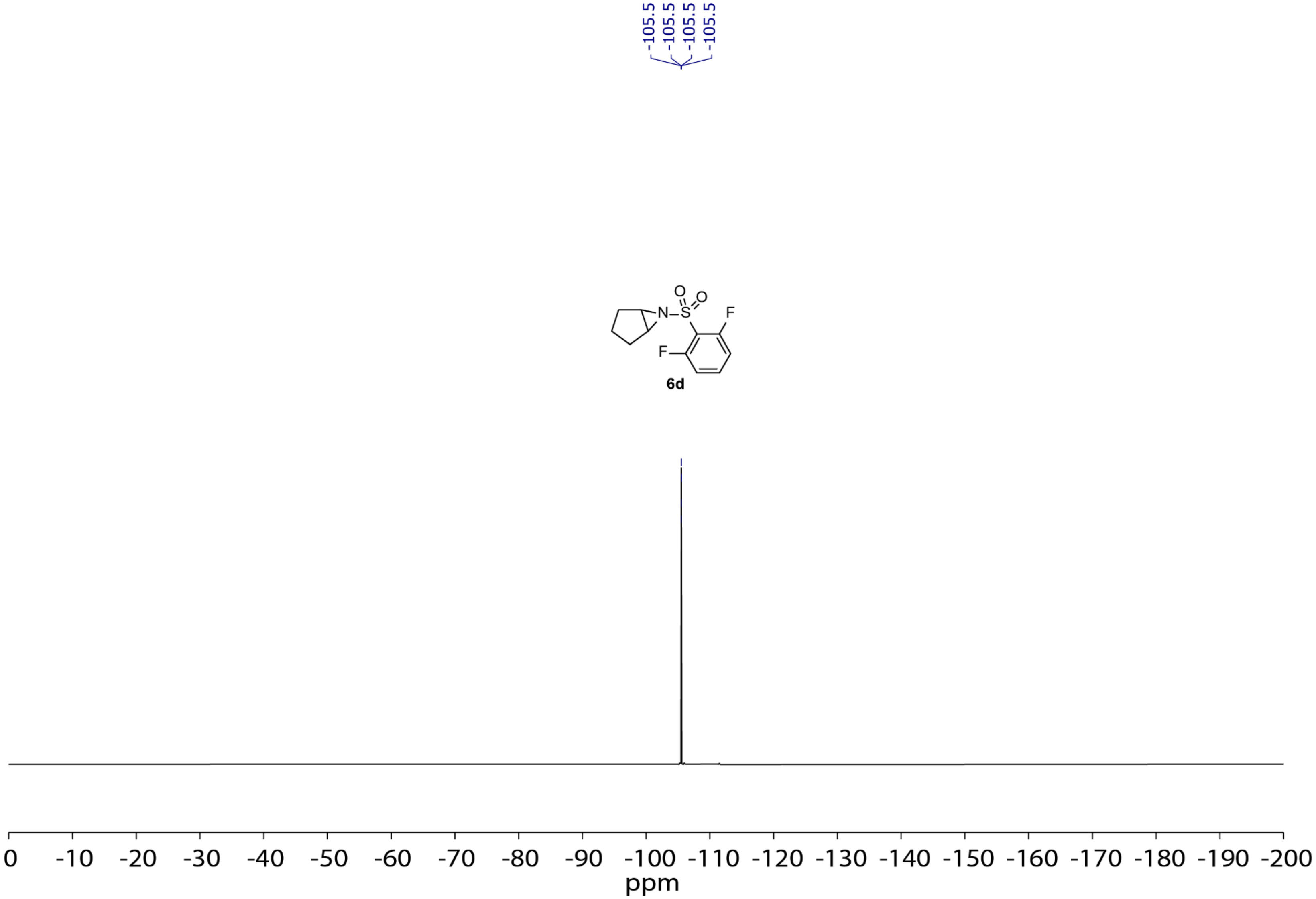

Figure S25. ^{19}F NMR spectrum of 6-((2,6-difluorophenyl)sulfonyl)-6-azabicyclo[3.1.0]hexane (**6d**) in $CDCl_3$ (377 MHz) at 23 °C.

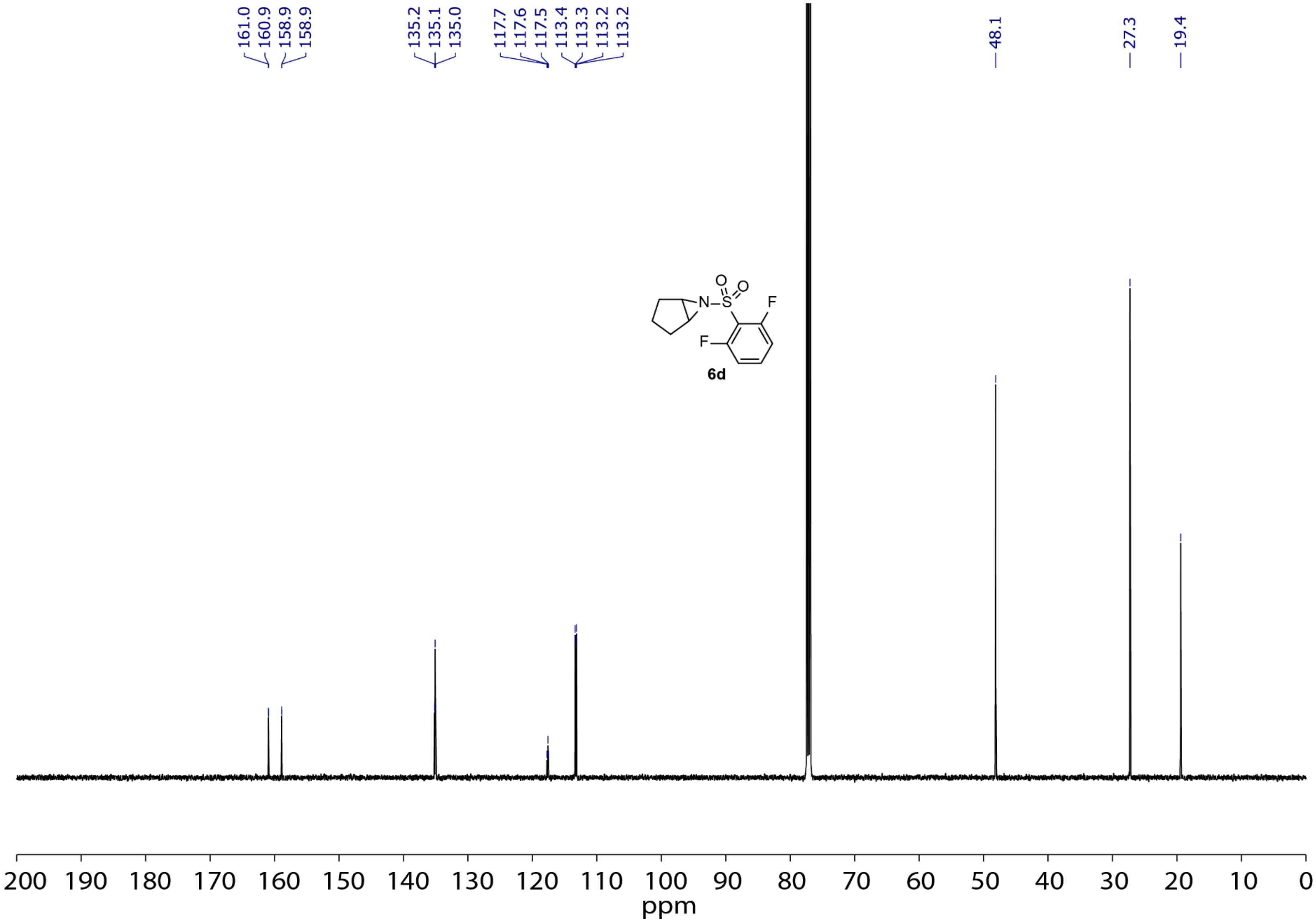

Figure S26. ^{13}C NMR spectrum of 6-((2,6-difluorophenyl)sulfonyl)-6-azabicyclo[3.1.0]hexane (**6d**) in $CDCl_3$ (126 MHz) at 23 °C.

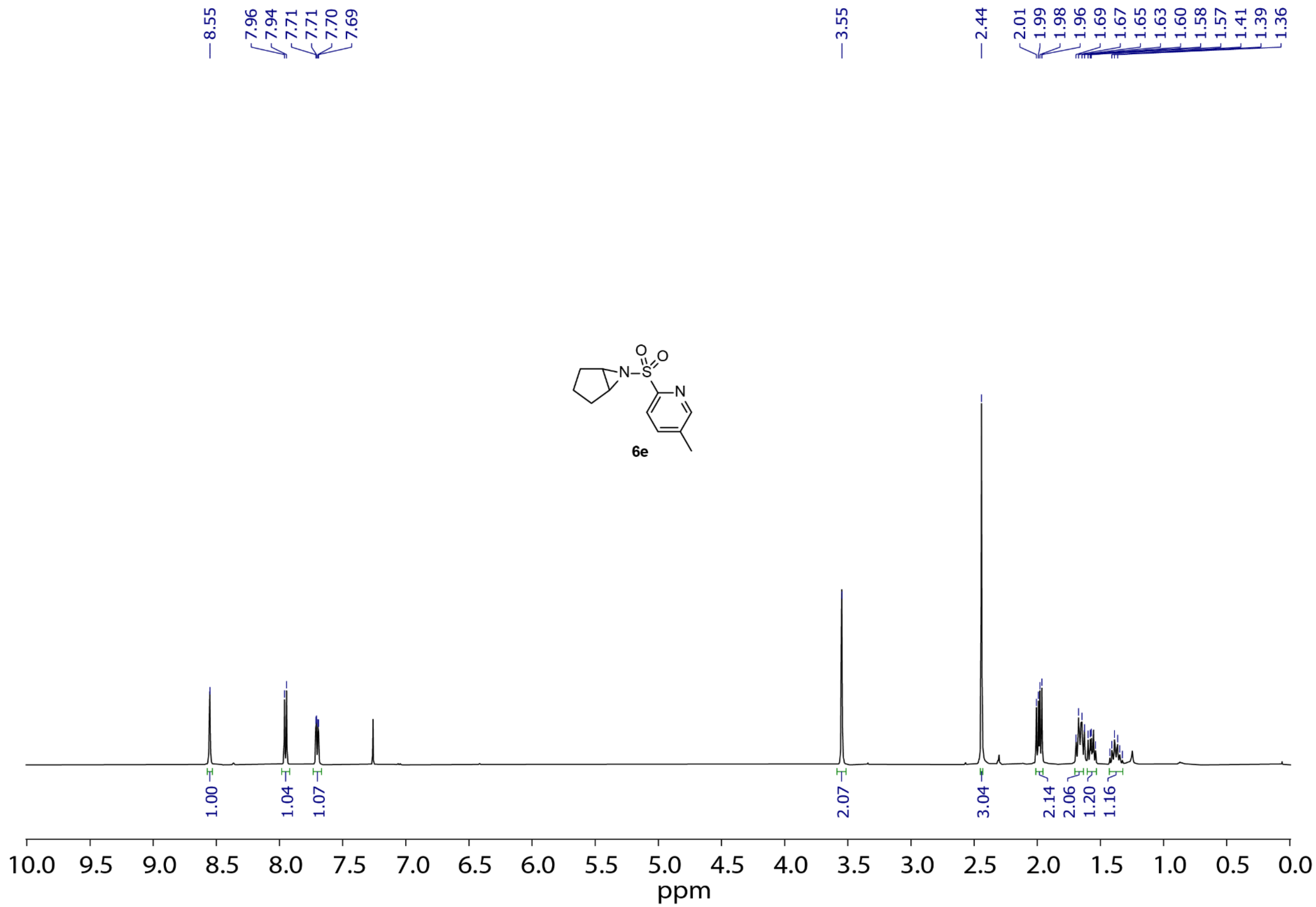

Figure S27. ^{1}H NMR spectrum of 6-((5-methylpyridin-2-yl)sulfonyl)-6-azabicyclo[3.1.0]hexane (**6e**) in $CDCl_3$ (400 MHz) at 23 °C.

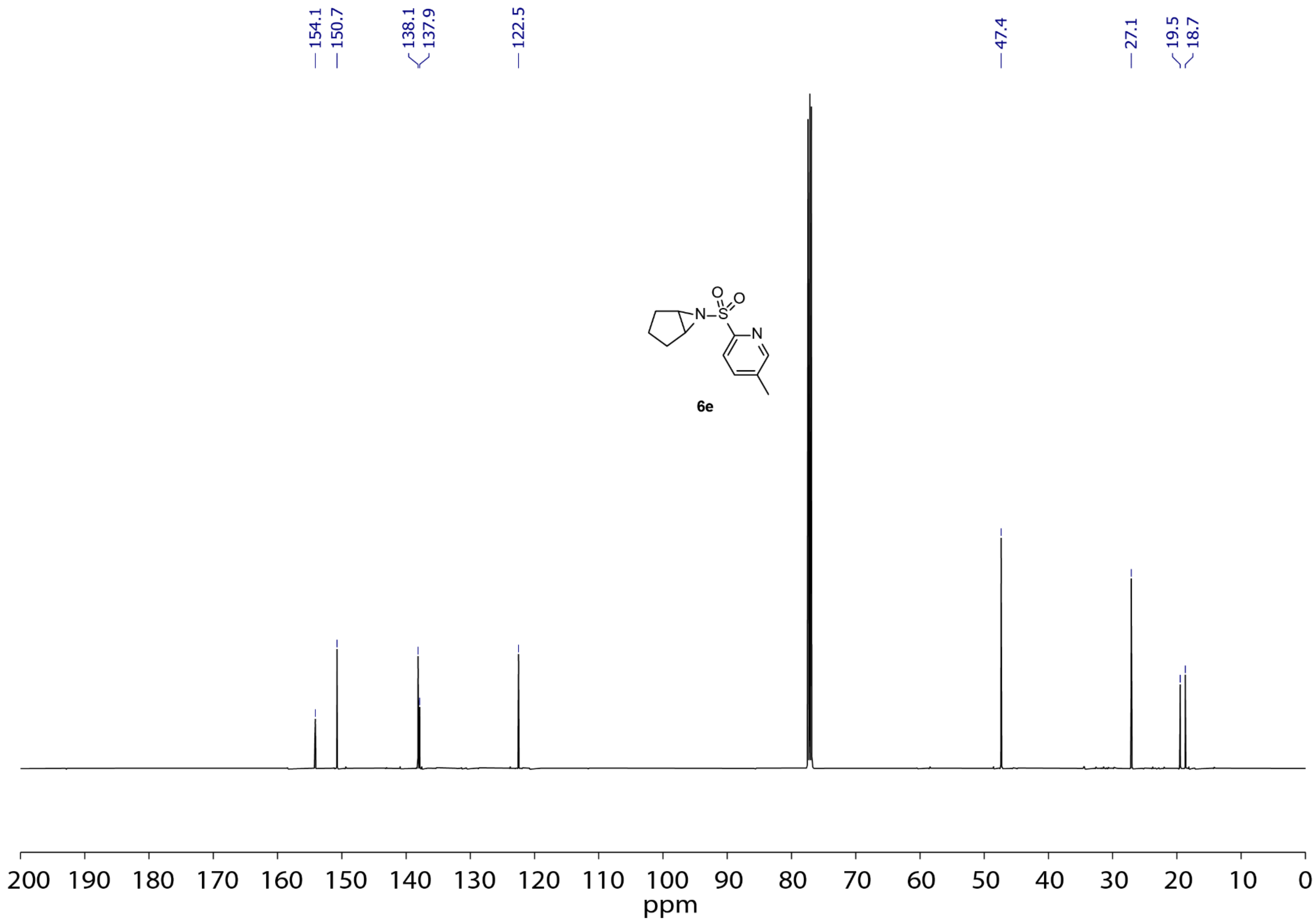

Figure S28. ^{13}C NMR spectrum of 6-((5-methylpyridin-2-yl)sulfonyl)-6-azabicyclo[3.1.0]hexane (**6e**) in $CDCl_3$ (126 MHz) at 23 °C.

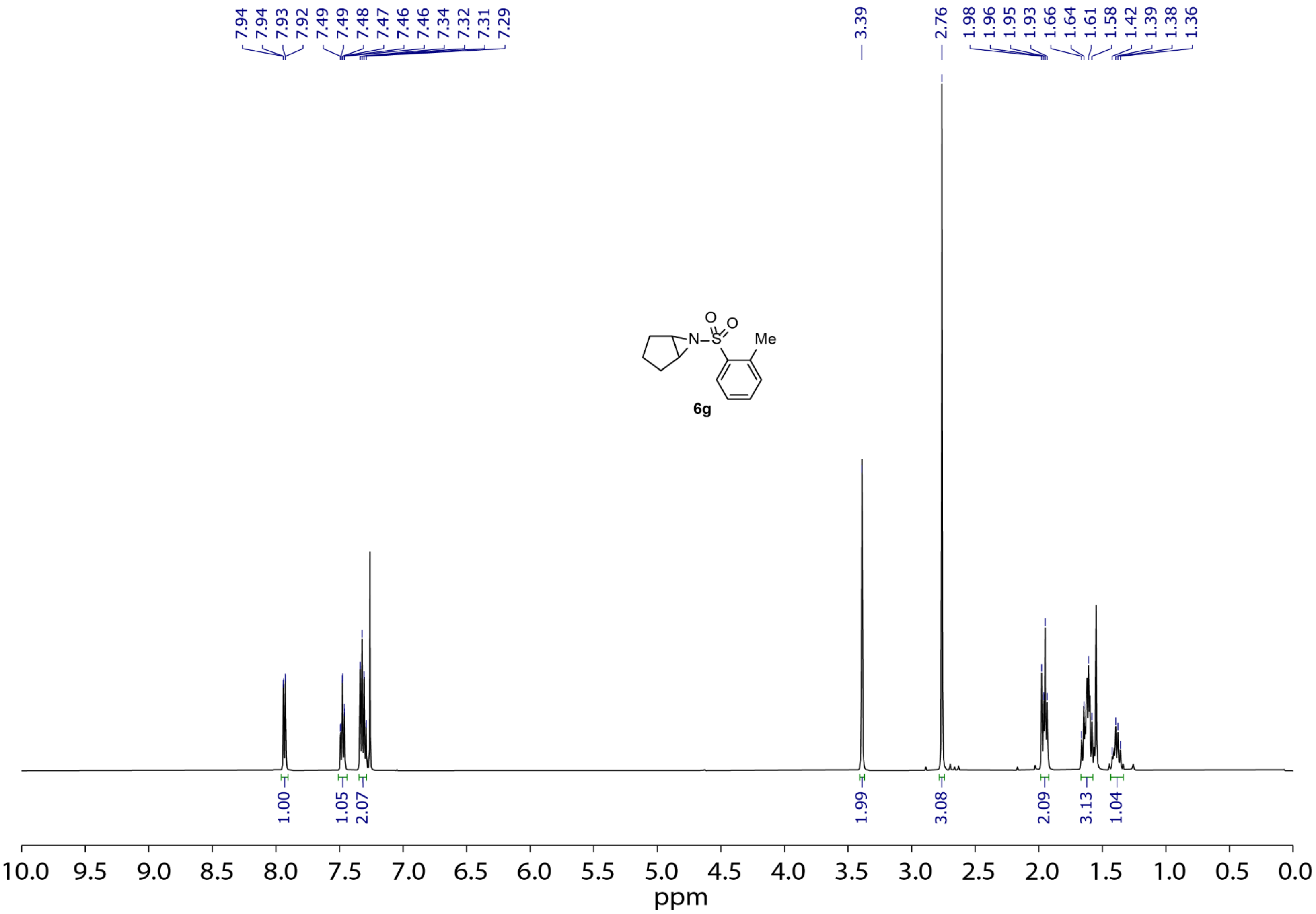

Figure S29. ^{1}H NMR spectrum of 6-(*o*-tolylsulfonyl)-6-azabicyclo[3.1.0]hexane (**6g**) in $CDCl_3$ (400 MHz) at 23 °C.

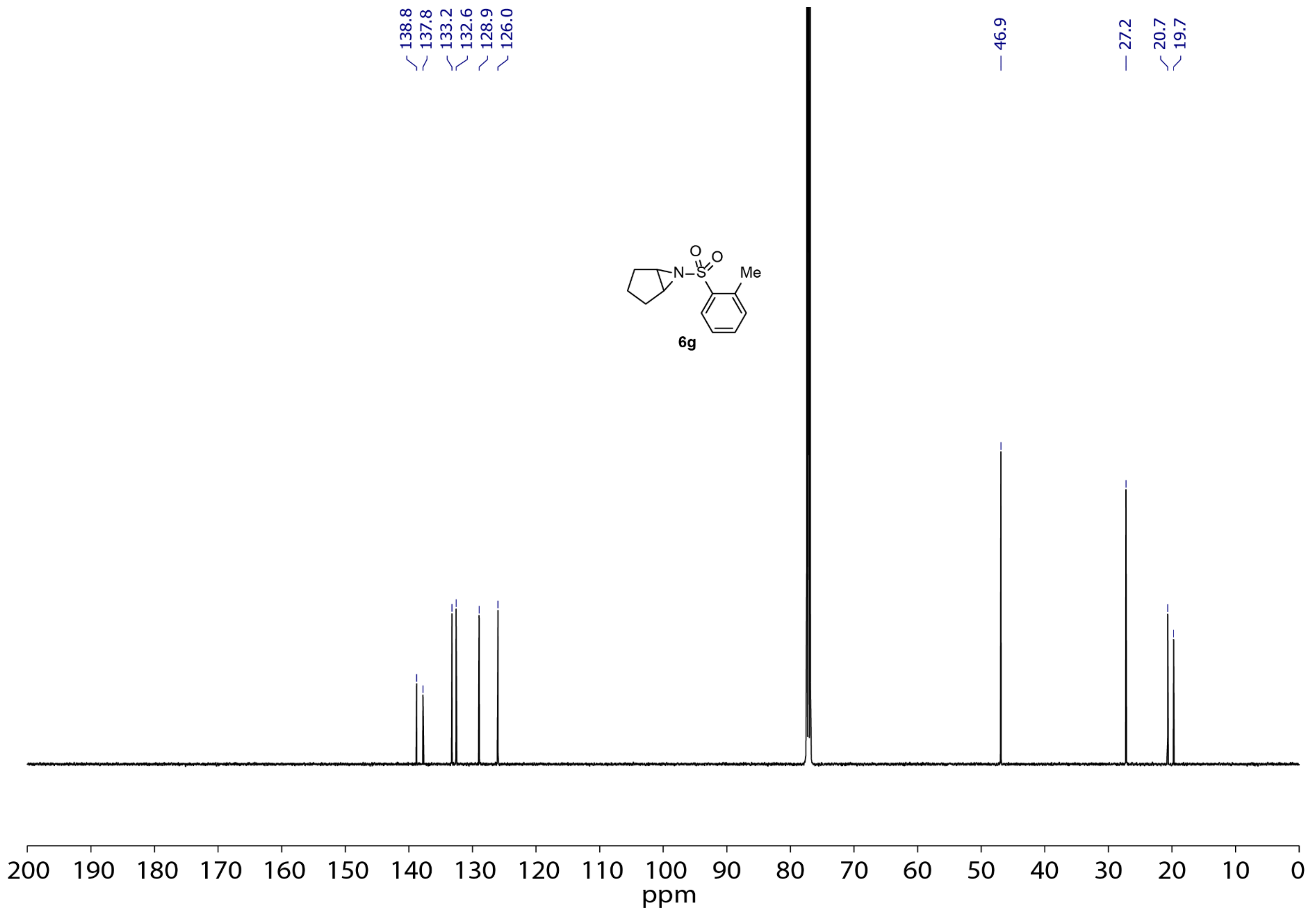

Figure S30. ^{13}C NMR spectrum of 6-(*o*-tolylsulfonyl)-6-azabicyclo[3.1.0]hexane (**6g**) in $CDCl_3$ (126 MHz) at 23 °C.

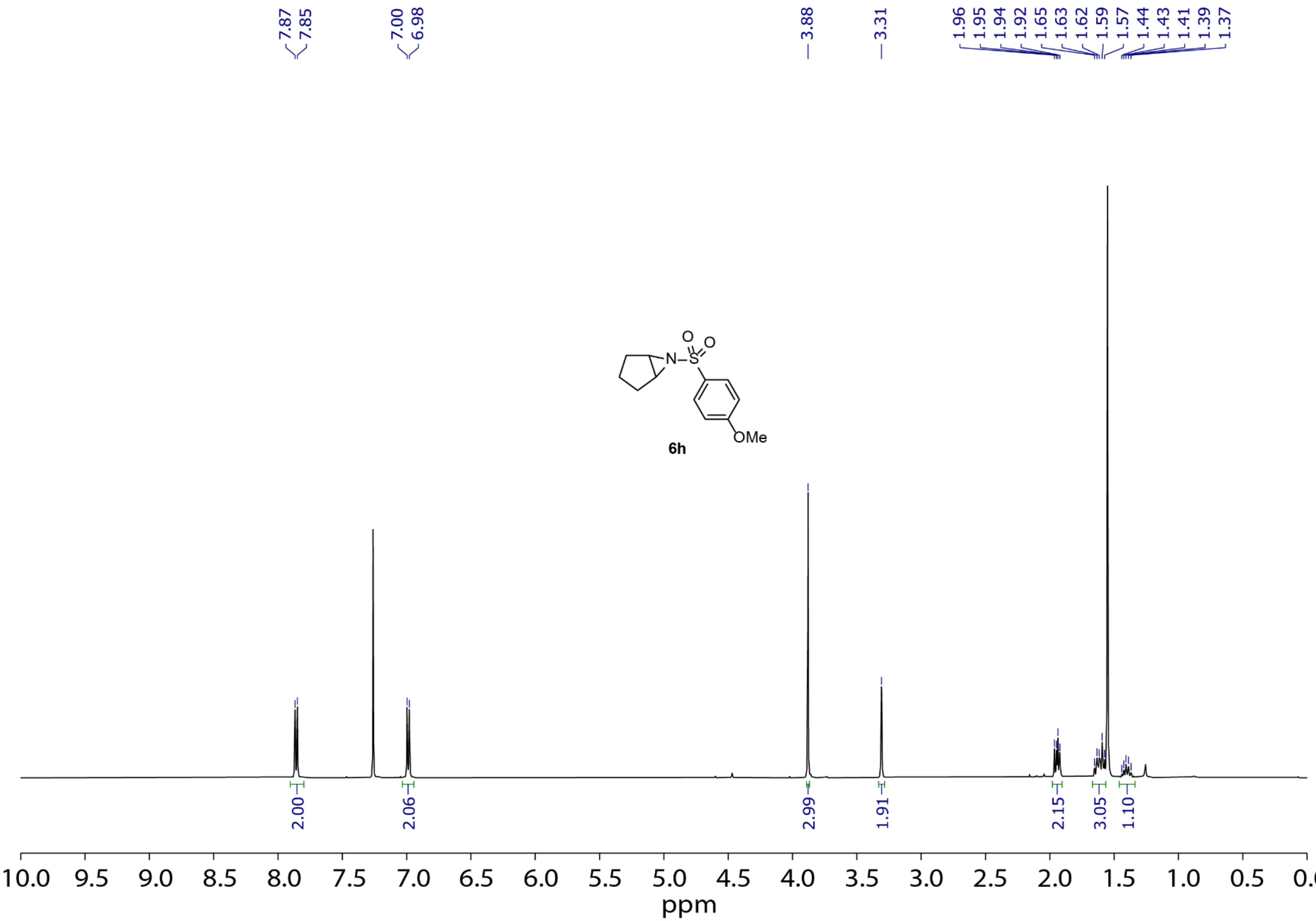

Figure S31. ^{1}H NMR spectrum of 6-((4-methoxyphenyl)sulfonyl)-6-azabicyclo[3.1.0]hexane (**6h**) in $CDCl_3$ (400 MHz) at 23 °C.

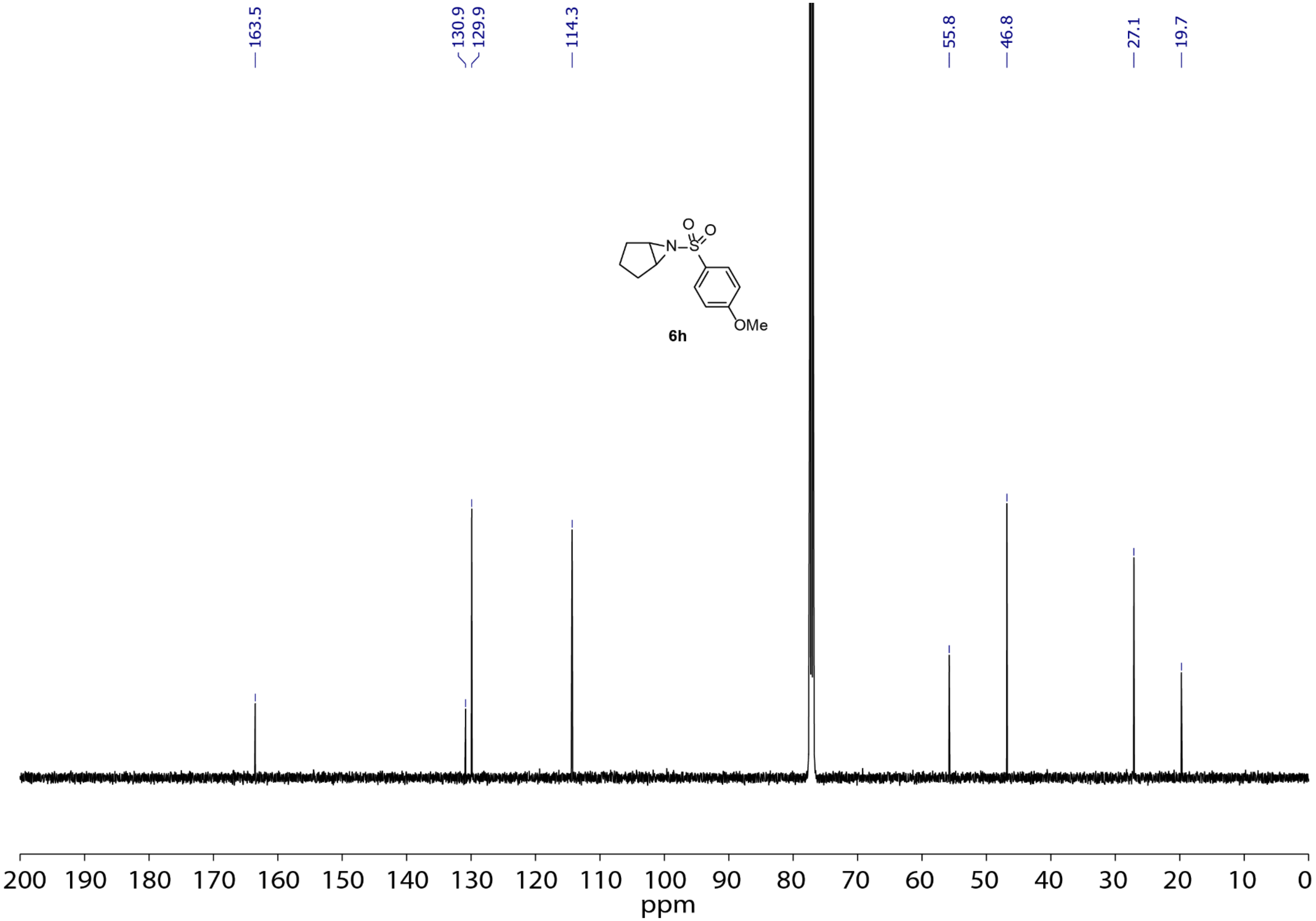

Figure S32. ^{13}C NMR spectrum of 6-((4-methoxyphenyl)sulfonyl)-6-azabicyclo[3.1.0]hexane (**6h**) in $CDCl_3$ (126 MHz) at 23 °C.

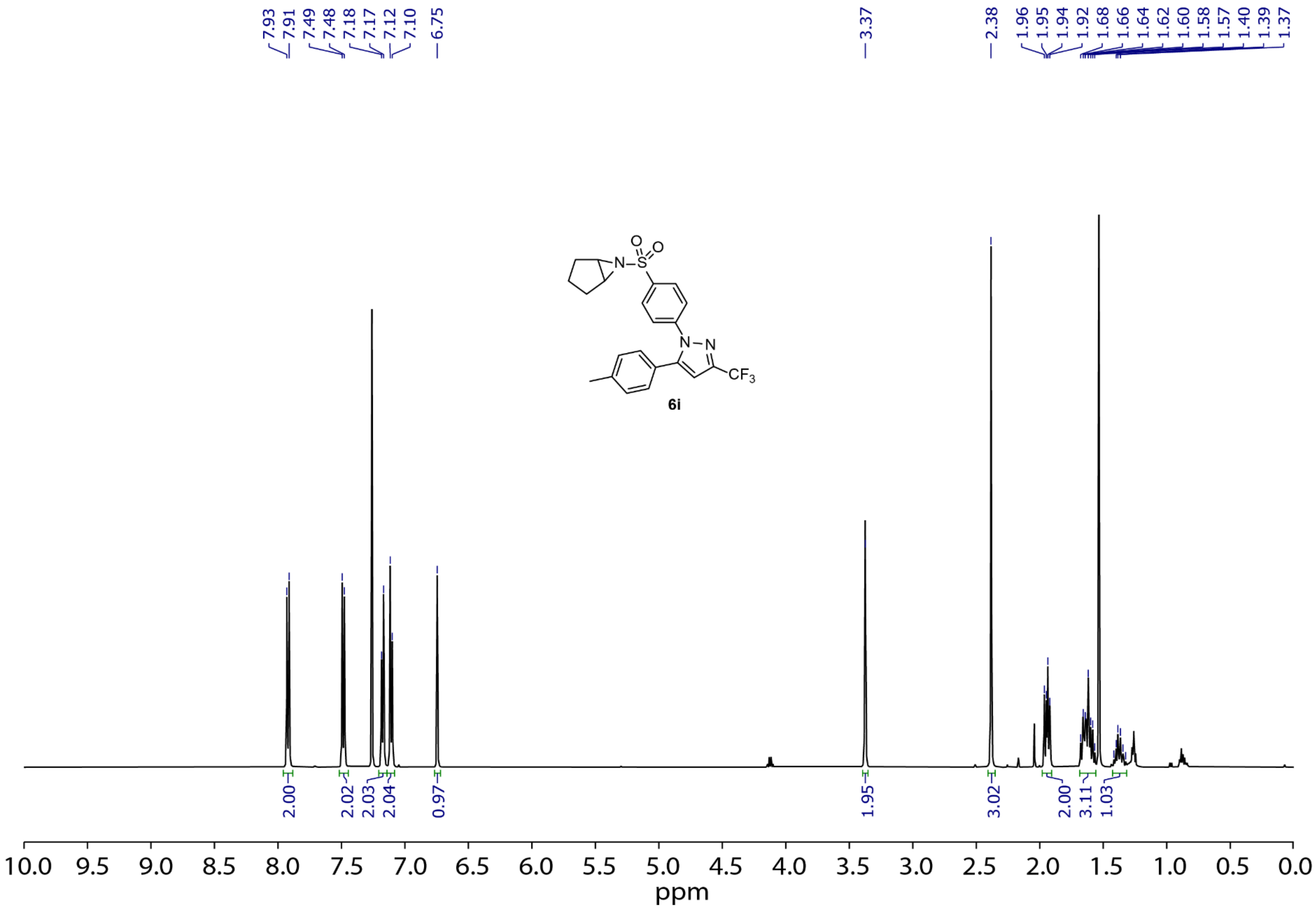

Figure S33. ^{1}H NMR spectrum of 6-((4-(5-(*p*-tolyl)-3-(trifluoromethyl)-1*H*-pyrazol-1-yl)phenyl)sulfonyl)-6-azabicyclo[3.1.0]hexane (**6i**) in $CDCl_3$ (400 MHz) at 23 °C.

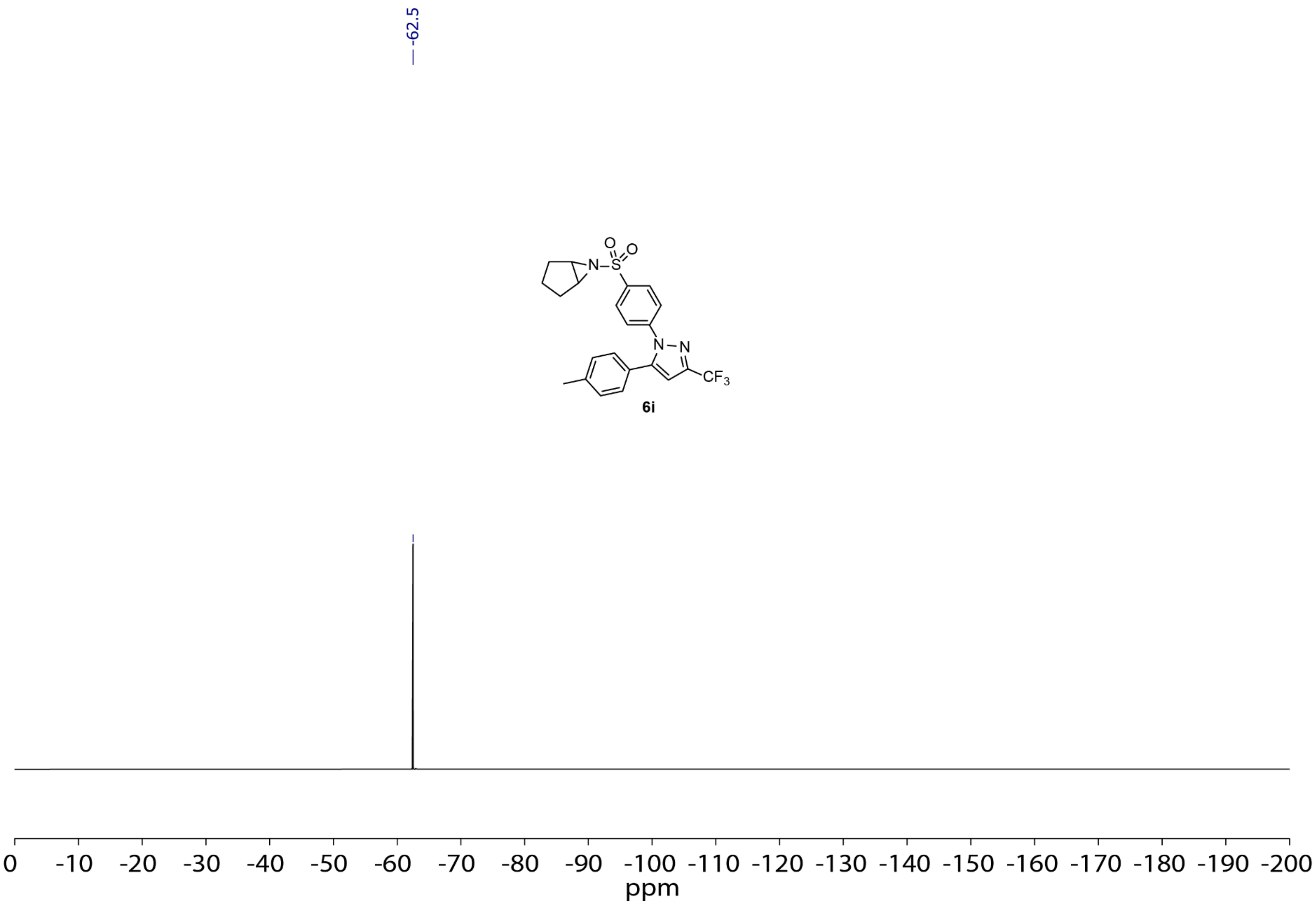

Figure S34. ^{19}F NMR spectrum of 6-((4-(5-(*p*-tolyl)-3-(trifluoromethyl)-1*H*-pyrazol-1-yl)phenyl)sulfonyl)-6-azabicyclo[3.1.0]hexane (**6i**) in $CDCl_3$ (377 MHz) at 23 °C.

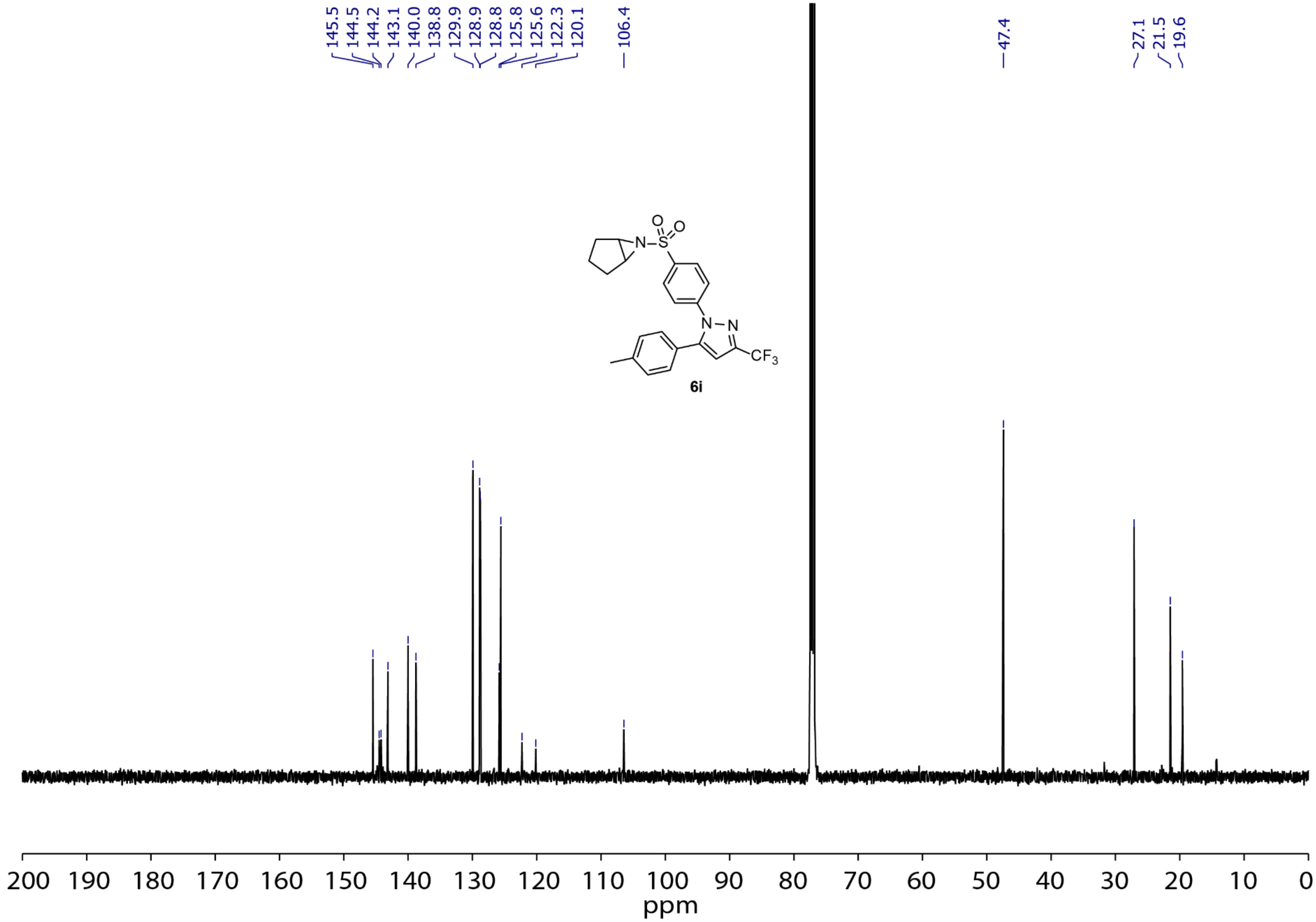

Figure S35. ^{13}C NMR spectrum of 6-((4-(5-(*p*-tolyl)-3-(trifluoromethyl)-1*H*-pyrazol-1-yl)phenyl)sulfonyl)-6-azabicyclo[3.1.0]hexane (**6i**) in $CDCl_3$ (126 MHz) at 23 °C.

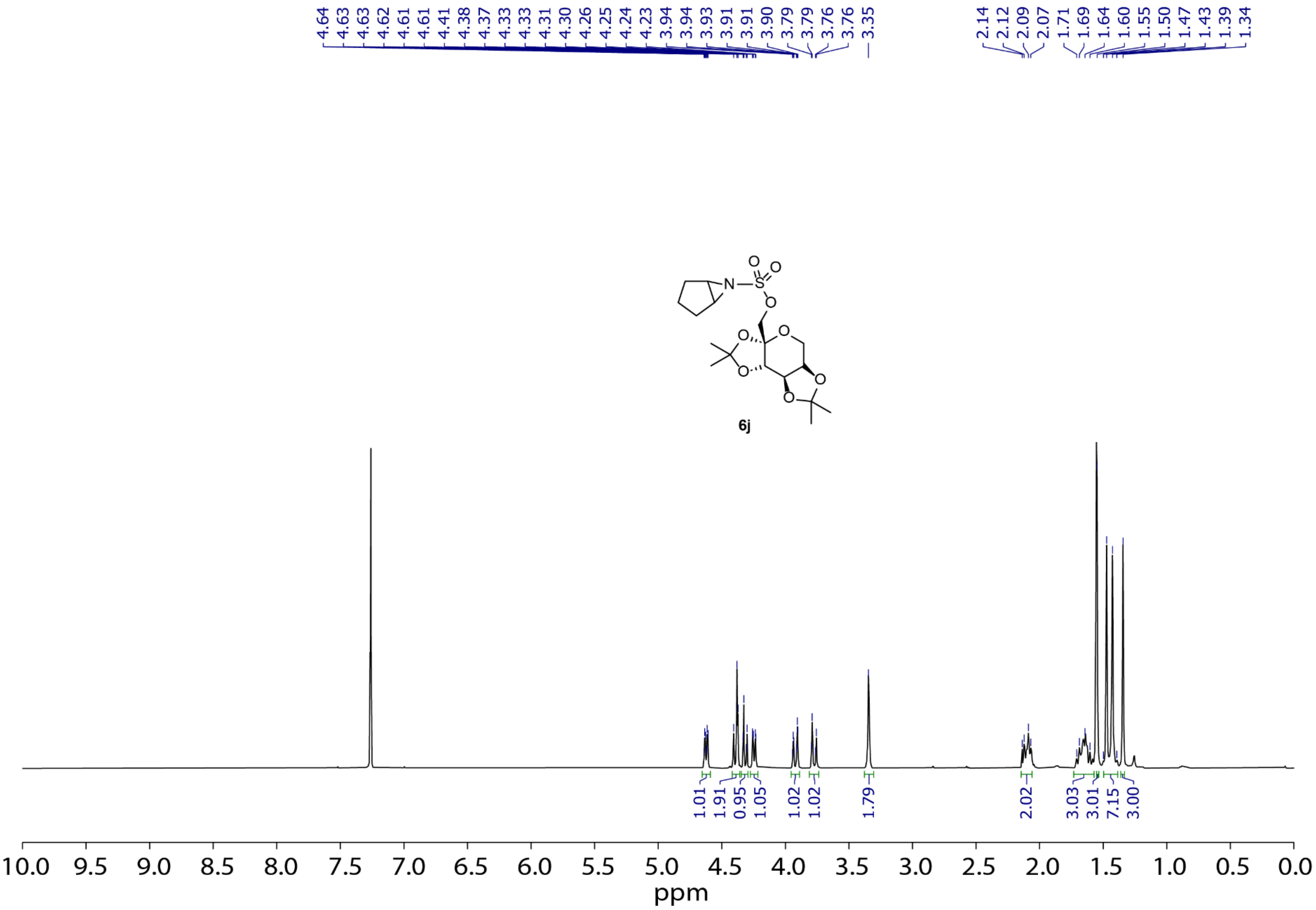

Figure S36. ^{1}H NMR spectrum of ((3a*S*,5a*S*,8a*R*,8b*S*)-2,2,7,7-tetramethyltetrahydro-3a*H*-bis([1,3]dioxolo)[4,5-*b*:4',5'-*d*]pyran-3a-yl)methyl 6-azabicyclo[3.1.0]hexane-6-sulfonate (**6j**) in $CDCl_3$ (400 MHz) at 23 °C.

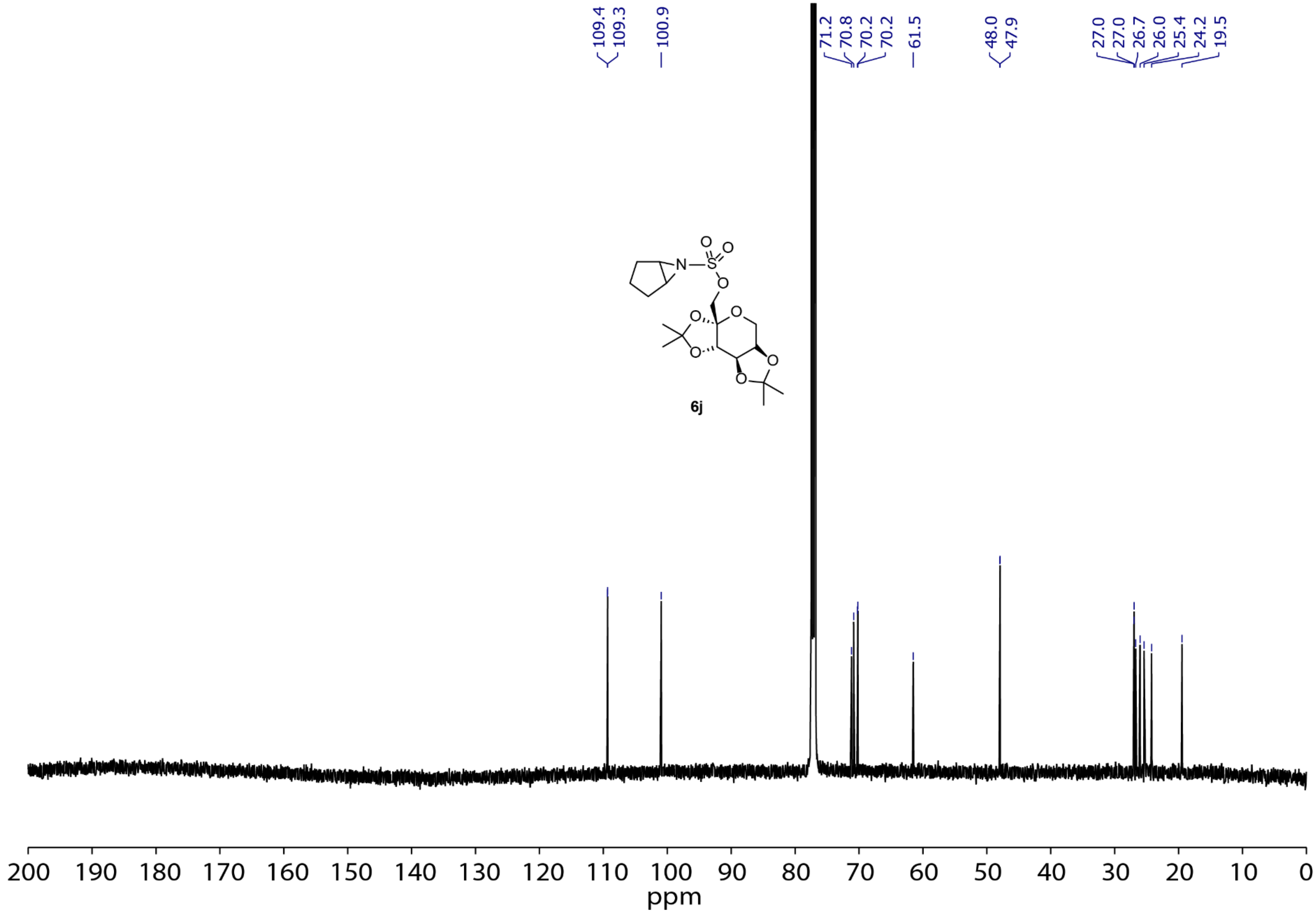

Figure S37. ^{13}C NMR spectrum of ((3a*S*,5a*S*,8a*R*,8b*S*)-2,2,7,7-tetramethyltetrahydro-3a*H*-bis([1,3]dioxolo)[4,5-*b*:4',5'-*d*]pyran-3a-yl)methyl 6-azabicyclo[3.1.0]hexane-6-sulfonate (**6j**) in $CDCl_3$ (126 MHz) at 23 °C.

F. Supplementary References

1. Armarego, W. L. F.; Chai, C. L. L. Chapter 4 - Purification of Organic Chemicals. In *Purification of Laboratory Chemicals (Sixth Edition)*, Armarego, W. L. F.; Chai, C. L. L., Eds. Butterworth-Heinemann: Oxford, 2009; pp 88–444.
2. Pangborn, A. B.; Giardello, M. A.; Grubbs, R. H.; Rosen, R. K.; Timmers, F. J. *Organometallics* **1996,** *15*, 1518–1520.
3. Tan, H.; Thai, P.; Sengupta, U.; Deavenport, I. R.; Kucifer, C. M.; Powers, D. C. Metal-Free Aziridination of Unactivated Olefins via Transient *N*-Pyridinium Iminoiodinanes. *ChemRxiv* **2024**, 10.26434/chemrxiv-2024-s8gcj.
4. Müller, P.; Baud, C.; Jacquier, Y. *Can. J. Chem.* **1998**, *76*, 738–750.
5. Youn, S. W.; Pastine, S. J.; Sames, D. *Org. Lett.* **2004**, *6*, 581–584.
6. Li, M.; Jin, Y.; Chen, Y.; Wu, W.; Jiang, H. *J. Am. Chem. Soc.* **2023**, *145*, 9448–9453.
7. Fulmer, G. R.; Miller, A. J. M.; Sherden, N. H.; Gottlieb, H. E.; Nudelman, A.; Stoltz, B. M.; Bercaw, J. E.; Goldberg, K. I. *Organometallics* **2010**, *29*, 2176–2179.
8. Wang, P.; Luchowska-Stańska, U.; van Basten, B.; Chen, H.; Liu, Z.; Wiejak, J.; Whelan, P.; Morgan, D.; Lochhead, E.; Barker, G.; Rehmann, H.; Yarwood, S. J.; Zhou, J. *J. Med. Chem.* **2020**, *63*, 5159–5184.
9. Hayashi, E.; Yamaguchi, Y.; Kita, Y.; Kamata, K.; Hara, M. *Chem. Commun.* **2020**, *56*, 2095–2098.
10. Pagnozzi, D.; Pala, N.; Biosa, G.; Dallocchio, R.; Dessì, A.; Singh, P. K.; Rogolino, D.; Di Fiore, A.; De Simone, G.; Supuran, C. T.; Sechi, M. *ACS Med. Chem. Lett.* **2022**, *13*, 271–277.
11. Jurberg, I. D.; Nome, R. A.; Crespi, S.; Atvars, T. D. Z.; König, B. *Adv. Synth. Catal.* **2022**, *364*, 4061–4068.
12. van Leest, N. P.; Grooten, L.; van der Vlugt, J. I.; de Bruin, B. *Chem. Eur. J.* **2019**, *25*, 5987–5993.
13. Nakanishi, M.; Salit, A.-F.; Bolm, C. Iron-Catalyzed Aziridination Reactions. *Adv. Synth. Catal.* **2008**, *350*, 1835–1840.
14. Sunagawa, S.; Morisaki, F.; Baba, T.; Tsubouchi, A.; Yoshimura, A.; Miyamoto, K.; Uchiyama, M.; Saito, A. *Org. Lett.* **2022**, *24*, 5230–5234.
15. Yang, S.; Hu, H.; Chen, M. *Org. Lett.* **2023**, *25*, 7968–7973.
16. Chen, M.; Wu, Z. J.; Song, J.; Xu, H. C. *Angew. Chem. Int. Ed.* **2022**, *61*, e202115954.
17. Li, X.; He, S.; Song, Q. *Org. Lett.* **2021**, *23*, 2994–2999.
18. Yoshimura, A.; Nemykin, V. N.; Zhdankin, V. V. *Chem. Eur. J.* **2011**, *17*, 10538–10541.
19. Park, S.; Koo, J.; Kim, W.; Lee, H. G. *Chem. Commun.* **2022**, *58*, 3767–3770.
20. Steiman, T. J.; Liu, J.; Mengiste, A.; Doyle, A. G. *J. Am. Chem. Soc.* **2020**, *142*, 7598–7605.
21. Liu, M. S.; Du, H. W.; Cui, J. F.; Shu, W. *Angew. Chem.* **2022**, *134*, e202209929.
22. Davies, J.; Janssen-Müller, D.; Zimin, D. P.; Day, C. S.; Yanagi, T.; Elfert, J.; Martin, R. *J. Am. Chem. Soc.* **2021**, *143*, 4949–4954.
23. Gross, P.; Im, H.; Laws III, D.; Park, B.; Baik, M.-H.; Blakey, S. B. *J. Am. Chem. Soc.* **2024**, *146*, 1447–1454.
24. Kirby, G.; Grimaud, L.; Vitale, M. R.; Prestat, G.; Berhal, F. *Green Chem.* **2021**, *23*, 9428–9432.
25. Wang, J.; Luo, M. P.; Gu, Y. J.; Liu, Y. Y.; Yin, Q.; Wang, S. G. *Angew. Chem. Int. Ed.* **2024**, *63*, e202400502.

26. Lux, M. C.; Jurczyk, J.; Lam, Y.-h.; Song, Z. J.; Ma, C.; Roque, J. B.; Ham, J. S.; Sciammetta, N.; Adpressa, D.; Sarpong, R. *Org. Lett.* **2020**, *22*, 6578–6583.
27. Li, Y.; He, J.; Khankhoje, V.; Herdtweck, E.; Köhler, K.; Storcheva, O.; Cokoja, M.; Kühn, F. E. *Dalton Trans.* **2011**, *40*, 5746–5754.
28. Verma, K.; Mishra, M.; Maharana, P. K.; Bhattacharyya, H.; Saha, S.; Punniyamurthy, T. *Org. Lett.* **2023**, *25*, 7933–7938.
29. O'Brien, P.; Pilgram, C. D. *Org. Biomol. Chem.* **2003**, *1*, 523–534.
30. Lardy, C.; Guerrier, D.; Chavernac, G.; Collonges, F., Substituted sulfonamides, process of preparation and medicines containing same. U.S. Patent 5,387,709, Feb 15, 1995.
31. Xu, Q.; Appella, D. H. *Org. Lett.* **2008**, *10*, 1497–1500.
